# *In Situ* UV–Vis Spectroscopy Insights into the Role of Hole Scavengers during the Photodeposition of Uniform Gold Nanoparticles on Zinc Oxide

**DOI:** 10.1021/acs.jpcc.6c03740

**Published:** 2026-10-10

**Authors:** Roos L. Grote, Elaina Galvin, Nico van Mil, Ariq Attallah, Emiel J. M. Hensen, Erik C. Garnett, Bert M. Weckhuysen, Eline M. Hutter

**Affiliations:** † Institute for Sustainable and Circular Chemistry, Department of Chemistry, Faculty of Science, Utrecht University, Universiteitsweg 99, 3584 CG Utrecht, The Netherlands; ‡ AMOLF, Science Park 104, 1098 XG Amsterdam, The Netherlands; § Laboratory of Inorganic Materials and Catalysis, Department of Chemical Engineering and Chemistry, Eindhoven University of Technology, 5600 MB Eindhoven, The Netherlands

## Abstract

Photodeposition has emerged as a promising technique to grow metal nanoparticles (NPs) on semiconductors through the reduction of metal ions by photoexcited electrons. It is known that metal NPs become more uniform when holes are subtracted by a hole scavenger. However, mechanistic insights into the effect of hole scavengers on the growth kinetics and resulting material properties and the effect of the type of hole scavenger are lacking. In this work, we use *in situ* UV–vis transmission spectroscopy to investigate the oxygen-deficient photodeposition of Au on ZnO. These measurements reveal that the presence of ethanol as a hole scavenger leads to faster nucleation and substantially accelerates the growth of Au NPs compared to the nonscavenged case. Although the effect of adding a hole scavenger is significant, the type of hole scavenger does not play a significant role in the final Au NP morphology. Therefore, adding a hole scavenger results in a higher density of Au NPs with a well-defined localized surface plasmon resonance (LSPR) band and a homogeneous size distribution of 9 ± 3 nm. By capturing real-time NP growth dynamics, inaccessible to *ex situ* methods, this study shows why a hole scavenger plays a significant role in the kinetics of Au NPs formation and why they lead to better-defined Au NPs with more controlled plasmonic properties.

## Graphical abstract

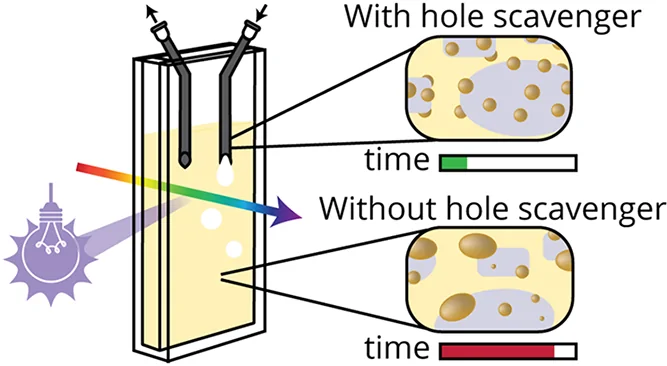

## Introduction

The increasing demand for environmentally friendly chemical processes includes more sustainable preparation techniques. In this context, photodeposition has emerged as a promising technique to grow metal nanoparticles (NPs) on semiconductors, which are widely used in catalysis, sensing, and optoelectronics. Photodeposition offers several benefits compared to conventional preparation methods (*e.g*., impregnation or deposition precipitation), including simplicity, shorter reaction times, lower cost, and lower environmental impact.^1,2^ The ability to control NP morphology (*i.e.*, size, shape, and distribution) is critical for optimizing material performance, yet the mechanistic pathways governing these processes remain poorly understood for many systems.

From previous works on the photodeposition of various (noble) metal NPs on different semiconductor materials, it is known that several parameters have an apparent influence during photodeposition, such as pH, irradiation time, and the use of hole scavengers *i.e.*, compounds that extract photogenerated holes and thereby suppress electron–hole recombination.^3–8^ Multiple studies have indicated the importance of including a hole scavenger, however their exact role and interactions during photodeposition varies between systems.^6^ For example, platinum (Pt) photodeposition on tungsten oxide (WO_3_) in the presence of methanol results in large, clustered Pt NPs up to 55 nm, whereas gold (Au) photodeposition on zinc oxide (ZnO) with methanol as hole scavenger results in uniform Au NP size distributions around 12 nm.^5,9^ While the effects of hole scavengers have been explored in systems, the Au/ZnO system is less studied.^6,9^ ZnO is a widely studied and applied n-type semiconductor with distinctive properties, with a bandgap in the UV region (∼3.3 eV).^10^

Combining ZnO with plasmonic Au NPs expands its absorption to the visible light region.^4,11–13^ That is, Au NPs have the ability to absorb and scatter light efficiently at their localized surface plasmon resonance (LSPR), which is the frequency at which the oscillations of the free electrons of Au (plasmons) are in resonance with the incoming light. This leads to various effects including the ability to concentrate light to the near-field of the NPs, the creation of hot charge carriers, and subsequent heating of the lattice and surrounding environment.^14–18^ Moreover, upon UV illumination of Au/ZnO hybrids, electrons are separated and transferred from ZnO to Au, thereby reducing band-to-band recombination in ZnO.^6,19,20^ Due to both effects, the photoactivity of the Au/ZnO hybrid is higher compared to its separate counterparts. As such, photodeposited Au/ZnO has found applications in sensing,^21^ photocatalytic (degradation) reactions,^4,11–13,22,23^ and water splitting.^24^

A handful of studies on the photodeposition of Au on ZnO employ alcohols as hole scavengers, however none systematically investigate their mechanistic influence. In particular, the effect of hole scavengers on the kinetics and resulting material properties are not well understood. In this work, we investigate how the presence and type of four alcohol-based hole scavengers influence the oxygen-deficient photodeposition of Au on ZnO by combining *in situ* UV–vis transmission spectroscopy, *ex situ* morphological analysis, and electromagnetic simulations. The four hole scavengers chosen were methanol, ethanol, *iso*-propanol, and *tert*-butanol. We monitored the photodeposition reaction with and without a hole scavenger (ethanol) with *in situ* UV–vis transmission spectroscopy to observe the kinetics of the formation of plasmonic Au NPs. These measurements show that the presence of ethanol as a hole scavenger leads to faster nucleation and substantially accelerates the growth of Au NPs compared to the nonscavenged case.

*Ex situ*High-Resolution (Scanning) Transmission Electron Microscopy combined with Energy Dispersive X-ray spectroscopy (HR-(S)­TEM-EDX) results show that without a hole scavenger, a broad Au NP size and shape distribution and fewer nucleation sites are obtained. In contrast, the use of different alcohol-based scavengers results in a uniform spatial distribution of very similar Au NP sizes of 9 ± 3 nm. This leads to a well-defined Au LSPR band, as established with UV–vis spectroscopy in diffuse reflectance (DR) mode, while the optical response of Au NPs prepared without a hole scavenger shows a red-shifted LSPR peak with an additional shoulder at higher energies. Electromagnetic simulations suggest that the high energy LSPR peak relates to the majority of sphere-like Au NPs, which is overshadowed by the large absorption of large, higher-aspect-ratio Au NPs. This work demonstrates how *in situ* UV–vis transmission spectroscopy provides mechanistic and kinetic insights, enabling optimization of photodeposition for tailored Au/ZnO nanocomposite properties in photocatalysis.

## Experimental Methods

### Chemicals

The following materials were used without further purification: ZnO (99,99%, Sigma-Aldrich), hydrogen tetrachloroaurate­(III) trihydrate (HAuCl_4_·3H_2_O, 99.99%, Thermo Scientific Chemicals), methanol (99.85%, VWR Chemicals BDH), ethanol absolute (100%, TechniSolv), *iso*-propanol (98%, VWR), *tert*-butanol (99.0%, Sigma-Aldrich), polytetrafluoroethylene (PFTE, 35 μm, Sigma-Aldrich), and ultrapure water (UPW) equilibrated with CO_2_.

### Au/ZnO synthesis

250 mg of ZnO, an appropriate amount of HAuCl_4_·3H_2_O stock solution (to amount to 0.9 wt % Au on ZnO), *x* ml of a hole scavenger (hole scavenger:precursor = 4800:1 molar ratio), and UPW to obtain 14 mL of added liquids were added to specialized glassware covered with aluminum foil to prevent outward scattering of UV light. The suspension was stirred vigorously and purged with N_2_ (80 mL/min) for 30 min (60 exchanges of the reactor volume) to remove as much O_2_ as possible and to obtain adsorption/desorption equilibrium. The suspension was illuminated with a UV lamp (for emission spectrum see Supporting Information (SI) Figure 1, 33 mW/cm^2^) for 5 min at room temperature while stirring and N_2_ flow were constant. The resulting purple suspension was washed three times with UPW, centrifuged, and the resulting powder was dried at 80 °C. A control experiment with oxygen-rich conditions was done by performing the same experiment, but substituting nitrogen bubbling with compressed air bubbling.

### Characterization Techniques

Transmission Electron Microscopy (TEM) investigations were performed in a Talos L120C microscope operating at 120 kV. High Resolution Scanning TEM (HR-STEM) measurements and elemental mapping were performed in a Talos F200 instrument, operating at 200 kV, using a high-brightness field emission gun (X-FEG) and a Super-X G2 EDX detector. The material was suspended in ethanol and deposited on a Formvar/Copper 200 mesh grid. The sizes of the Au NPs were manually determined using the software ImageJ. Multiple regions on the grid were sampled and >100 Au NPs per batch were measured, combining to at least 238 Au NPs measured per condition. High-resolution TEM images were used for lattice *d*-spacings of selected Au/ZnO particles. Powder Diffraction File 5 (PDF5) cards were used for the phase identification (Au: 00-004-0784, Au_2_O_3_: 00-043-1039, ZnO: 00-036-1451). Inductively coupled plasma - optical emission spectroscopy (ICP-OES) measurements were performed by Mikrolab Kolbe (Oberhausen, Germany). A single determination of the atomic concentrations of element Au was measured on a Spectro Model Spectro Arcos ICP after microwave digestion on a CEM MARS 6. X-Ray Photoelectron Spectroscopy (XPS) spectra were recorded on a Kα X-ray photoelectron spectrometer (Thermo Scientific) equipped with a monochromatic Al Kα X-ray source (*hν* = 1486.68 eV). Samples were mounted on a double-sided carbon tape. During acquisition the pressure in the analysis chamber was maintained below 8 × 10^–8^ mbar.

Binding energies were calibrated against the C 1s signal of adventitious carbon at 284.8 eV. Peak fitting was carried out using a Shirley background and four quasi-Voigt line shapes. For the spin–orbit doublets, the peak area ratios were constrained to 4:3 for Au 4f_7/2_/Au 4f_5/2_ and 2:1 for Zn 3p_3/2_/Zn 3p_1/2_. The full width at half-maximum (FWHM) was constrained to be identical for both components of each doublet, while the spin–orbit splittings were fixed at 3.67 eV for Au 4f and 2.7 eV for Zn 3p. The Au/ZnO powders were diluted in polytetrafluoroethylene (4 wt % sample in PTFE 35 μm) and characterized with *Diffuse Reflectance (DR) UV–vis spectroscopy* with a PerkinElmer Lambda 950S spectrophotometer equipped with an integrating sphere, a photomultiplier tube, and an indium gallium arsenide detector. The integrating sphere was configured to collect only the diffusely reflected light (excluding specular reflectance) at the exit of the integrating sphere with 35 μm PTFE as a white background. The DR data was related to the Kubelka–Munk (KM) function with

1 $$\mathrm{KM}=\frac{K}{S}=\frac{{\left(1-R_{\infty }\right)}^{2}}{2R_{\infty }}\;\mathrm{with}\;R_{\infty }=\%R/100$$

Where %*R* is the percentage of diffusely reflected light of a sample over that of the background), *K* = *KM* absorption coefficient, and *S* = *KM* scattering coefficient. This approach is standard for characterizing the optical properties of powdered samples, where transmission measurements are not feasible due to strong (diffuse) light scattering and limited light transmission. *In situ UV–vis transmission spectroscopy* was measured in a homemade setup on 8 g/L 0.7 wt % Au/ZnO suspensions in a 2 mm path-length cuvette equipped with a magnetic stirrer. For a photo with annotations and a schematic overview of the setup, see Figure S2. This setup measured transmitted photons through the suspension, in contrast to the DR measurements performed on powdered samples. A Leukos STM-2-UV supercontinuum laser (Limoges, France) with a spectral wavelength ranging from 350 to 1800 nm at a 2 kHz repetition rate and a pulse width (FWHM) of <1 ns was coupled into EOS to generate a continuous white light as a probe pulse. Figure S3 shows the response of the white light source (*I*_0_). The beam was passed through a 400 nm long-pass filter, to avoid ZnO excitation with the probe, and focused through the suspension. Transmitted light was passed through a 390 nm long-pass filter and focused on a fiber coupled spectrometer (OceanOptics, flame-t-vis-NIR-es) with an integration time of 100 ms. The cuvette was closed with a parafilm plug. The suspension was stirred and purged with N_2_ (7 mL/min) for 30 min in the dark through an inlet needle under the liquid level and an exhaust needle above the liquid level (300 exchanges of the reaction volume). After the equilibrium period, a UV flashlight from DarkBeam (Figure S1, 33 mW/cm^2^) perpendicular to the white light source was switched on. All measurements were performed under continuous stirring and nitrogen bubbling to maintain oxygen-deficient conditions and ensure sample homogeneity (see Figure S4 for an estimate of associated evaporative solvent loss). The *total UV–vis reflectance spectroscopy* was measured on 8 g/L 0.7 wt % Au/ZnO suspensions a 2 mm path-length cuvette equipped with a magnetic stirrer at the exit of the integrating sphere (of the PerkinElmer Lambda 950S spectrophotometer) with 35 μm PTFE in a 2 mm path-length cuvette as a white background. During N_2_ bubbling and UV illumination the suspension was not stirred. The transmission was excluded as the transmitted light was absorbed by a black background after passing through the cuvette. During these measurements, the specular exclusion port was not removed, thus the collected light included both diffuse and specular reflected light. The *UV–vis absorption spectroscopy* of the Au precursor solutions was measured in transmission mode in a 10 mm path length cuvette in the PerkinElmer Lambda 950S spectrophotometer. The absorbance was calculated with

2 $$A=-\log\left(\frac{I}{I_{0}}\right)$$

With *I* = Intensity transmitted through the sample and *I*_0_ = intensity of blank, in this case water.

### Electromagnetic Simulations

Electromagnetic simulations of single Au NPs on ZnO were carried out in Lumerical using the finite-difference time-domain (FDTD) method. The simulations considered single Au NPs and therefore did not account for interparticle near-field coupling, which may modify the LSPR position depending on particle separation and relative orientation.^25,26^ Based on the particle sizes and interparticle separations relevant to the samples studied here, near-field coupling is expected to have only a minor influence on the simulated spectra, as discussed further in the Supporting Information (SI, page S31). A range of 14 Au NPs were modeled as ellipsoidal spheres where *radius*, *radius 2*, and *radius 3* were input to the sphere geometry and are included in Table 1.

**Table 1:** Overview of the Length, Width, and Aspect Ratio (AR) of the Simulated Au Nanoparticles (NPs) and the Modeling Input Radii^a^

Nanoparticle number	Length (nm)	Width (nm)	Aspect Ratio	Radius (nm)	Radius 2 (nm)	Radius 3 (nm)
Rod 1	3.5	2.7	1.3	1.75	1.35	1.35
Rod 2	33	25.4	1.3	16.5	12.7	12.7
Rod 3	16.5	12.7	1.3	8.25	6.35	6.35
Rod 4	16.5	10.3	1.6	8.25	5.15	5.15
Rod 5	16.5	8.25	2.0	8.25	4.125	4.125
Rod 6	45	28.13	1.6	22.5	14.065	14.065
Rod 7	80	61.5	1.3	40	30.77	30.77
Sphere 1	5	5	1	2.5	2.5	2.5
Sphere 2	10	10	1	5	5	5
Sphere 3	16.5	16.5	1	8.25	8.25	8.25
Sphere 4	20	20	1	10	10	10
Sphere 5	30	30	1	15	15	15
Sphere 6	40	40	1	20	20	20
Sphere 7	50	50	1	25	25	25

a The particle length and AR are based on Au NPs obtained when preparing Au/ZnO without a hole scavenger (Figure S5).

The Au NPs were truncated by the ZnO surface, removing 20% of their height. The truncation of the Au on the ZnO surface was estimated to be 20% based on TEM analysis of a representative Au NP at the edge of a ZnO particle, see Figure S6. A fine mesh box was placed around the particle with a 20% buffer in *x*, *y*, and *z*, where the mesh size was determined by multiplying 0.01 × height of the NP to ensure accurate calculations. An absorption analysis group was used to calculate the absorption cross section of the Au NP on ZnO. The monitor was set with a 10% buffer around the Au NPs so that it was tight to the NPs and within the fine mesh box. Due to the fine mesh required to the simulation region was set to be as small as possible. For rods 1–6 and spheres 1–7, the 3D simulation region was set to be 3000 nm in *x* and *y* and *z*. For rod 7, the simulation region was set to 500 nm in *x*, *y*, and *z*. In all cases the simulation region was confined with eight layers of stretched perfectly matched layers (PMLs) to prevent any artificial reflections incurred from the edges of the simulation domain. A total-field-scattered-field (TFSF) source was used to excite the NPs from the top in the negative z direction. For NPs 1–7, the TFSF source was set to 100 nm in *x* and *y*, and *z* and for NP 8 it was set to 200 nm in *x*, *y*, and *z*. The simulation was run for two polarizations by setting the polarization angle to 0° to excite the longitudinal mode and 90° to excite and transverse mode. An average of the two polarizations was taken and used to compare with the unpolarized excitation in UV–vis spectroscopy experiments.

## Results and Discussion

To investigate the effect of adding a hole scavenger during photodeposition of Au on ZnO, we studied the growth kinetics using *in situ* UV–vis transmission spectroscopy. We selected ethanol as a representative hole scavenger because of its reproducibility. Aqueous suspensions of ZnO and HAuCl_4_ were illuminated with UV-light, generating electrons and holes in the ZnO conduction and valence band, respectively. These photogenerated electrons can reduce Au^3+^ (AuCl_4_^–^) ions to a stable intermediate complex AuCl_2_^–^ (Au^1+^), and subsequently to metal Au NP growth on ZnO (see Figure 1a for a schematic overview).^27–31^ We chose to perform the reaction in oxygen-deficient conditions, as oxygen reduction may compete with Au reduction. An oxygen-rich control experiment resulted in a substantially different DR UV–vis spectrum and a broad size and shape distribution with an average particle size of 18.9 ± 7.1 nm (For DR UV–vis, particle size analysis and STEM-EDX images see Figures S7, S8, and S9, respectively). The holes may either react with water, recombine with electrons, photodegrade ZnO, or transfer to a hole scavenger (for band positions, redox potentials, and reaction equations we refer to Figure S10).

**Figure 1:**
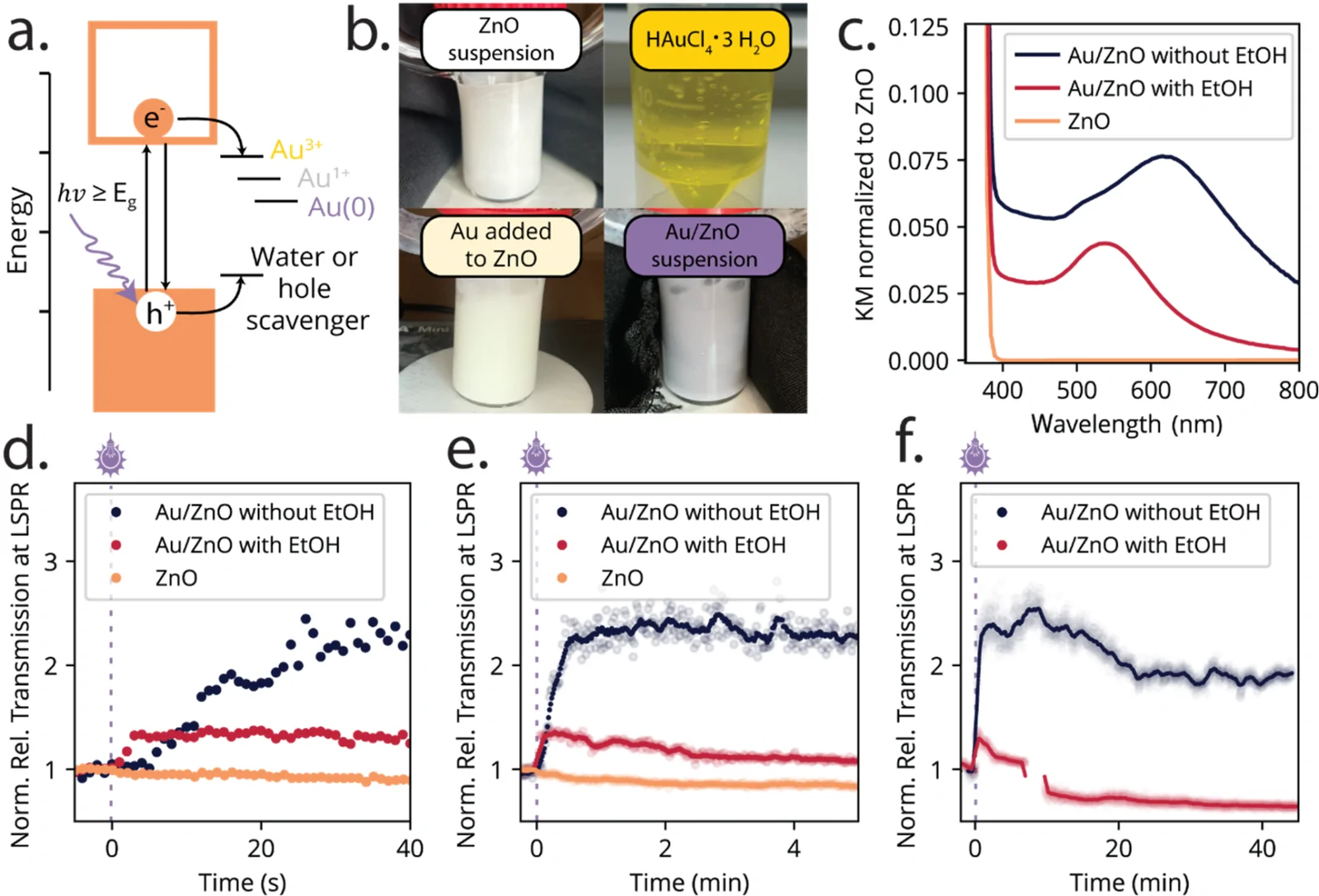
Schematic band diagram of ZnO with the photoreduction process of Au^3+^ to Au(0) and photo oxidation counter reactions (a). Photographs of ZnO suspension, HAuCl_4_·3 H_2_O precursor solution 12.6 g/L, ZnO suspension with Au precursor after 30 min of N bubbling in the dark and Au/ZnO suspension after 5 min of UV irradiation in the ex-situ synthesis glassware (b). Optical responses of ZnO and 0.9 wt % Au/ZnO materials synthesized in the absence and presence of ethanol (c). The KM values are derived from diffuse reflectance (DR) UV–vis spectroscopy measurements and converted into values proportional to absorption over the scattering via the Kubelka–Munk (KM) transformation (eq 1). All KM values are normalized to the absorption peak of ZnO. Transmission at Localized Surface Plasmon Resonance (LSPR) (Au/ZnO without EtOH: 512 nm, Au/ZnO with EtOH: 547 nm, and ZnO: 512 nm. These LSPR wavelengths were identified for each sample from the *ex situ* DR Spectroscopy measurements) divided to transmission at 900 nm and normalized to 1 at *t* = −0.4 s during the first 40 s of UV illumination (d) 5 min of UV illumination (e), and 45 min of UV illumination (f). In 1e, lighter data points are data points at 1 s intervals, whereas darker data points are a running average with a Δ*t* = 10 s. In 1f, lighter data points are data points at 1 s intervals, whereas darker data points are a running average with a Δ*t* = 30 s. For *ex situ* reflectance measurements, related to scattering, we refer to Figures S16–18.

Figure 1b shows the color change from the white ZnO suspension to light yellow when the yellow precursor HAuCl_4_ was added. After 5 min of UV exposure, the suspension turns purple showcasing the conversion of Au^3+^ to Au (0) NPs on the ZnO surface. This color change was quantified with *ex situ* UV–vis spectroscopy in diffuse reflectance (DR) mode by measuring the dried Au/ZnO powders. Figure 1c shows the optical response of bare ZnO powder and the dried Au/ZnO materials prepared in the presence and absence of ethanol. The values derived from DR UV–vis spectroscopy measurements were converted to the Kubelka–Munk (KM) function (proportional to the ratio of the absorption and scattering coefficients, *K*/*S*) (eq 1). While the KM function does not directly correspond to absorbance or absorptance, it allows for qualitative comparison of the plasmonic features of Au NPs on ZnO. All DR UV–vis spectra are normalized to the absorption band of ZnO for consistency. Bare ZnO shows a sharp onset below ∼385 nm, corresponding to its bandgap. In the spectra of Au/ZnO with and without ethanol, an additional absorption feature is observed, originating from the light absorption by Au centered between ∼540–650 nm. This absorption band shows the two overlapping transitions in Au: plasmon excitation, which gives rise to the LSPR peak, and the interband excitation, which appears as a background absorption that increases at higher photon energies.^17,32^ Here, we fitted the LSPR band in the KM signal as a function of energy in eV by subtracting a linear baseline and fitting two or three gaussians on the remaining signal (see Figures S11 and 12). Interestingly, Au photodeposition on ZnO without a hole scavenger produces split LSPR band with a peak at higher energies at 2.42 eV (512 nm) and a larger red-shifted LSPR at 1.99 eV (623 nm). The split band was observed in all syntheses prepared without a hole scavenger (Figure S13). This suggests the coexistence of multiple NP populations or possibly different plasmon modes due to anisotropic particles (explored further in Figure 2). Conversely, the Au LSPR band for samples prepared with ethanol as a hole scavenger are well-defined with an average LSPR peak at 2.27 ± 0.1 eV (547 ± 1.4 nm) for samples prepared with a hole scavenger. These results suggest a more controlled Au NP growth when ethanol is added as a hole scavenger.

**Figure 2:**
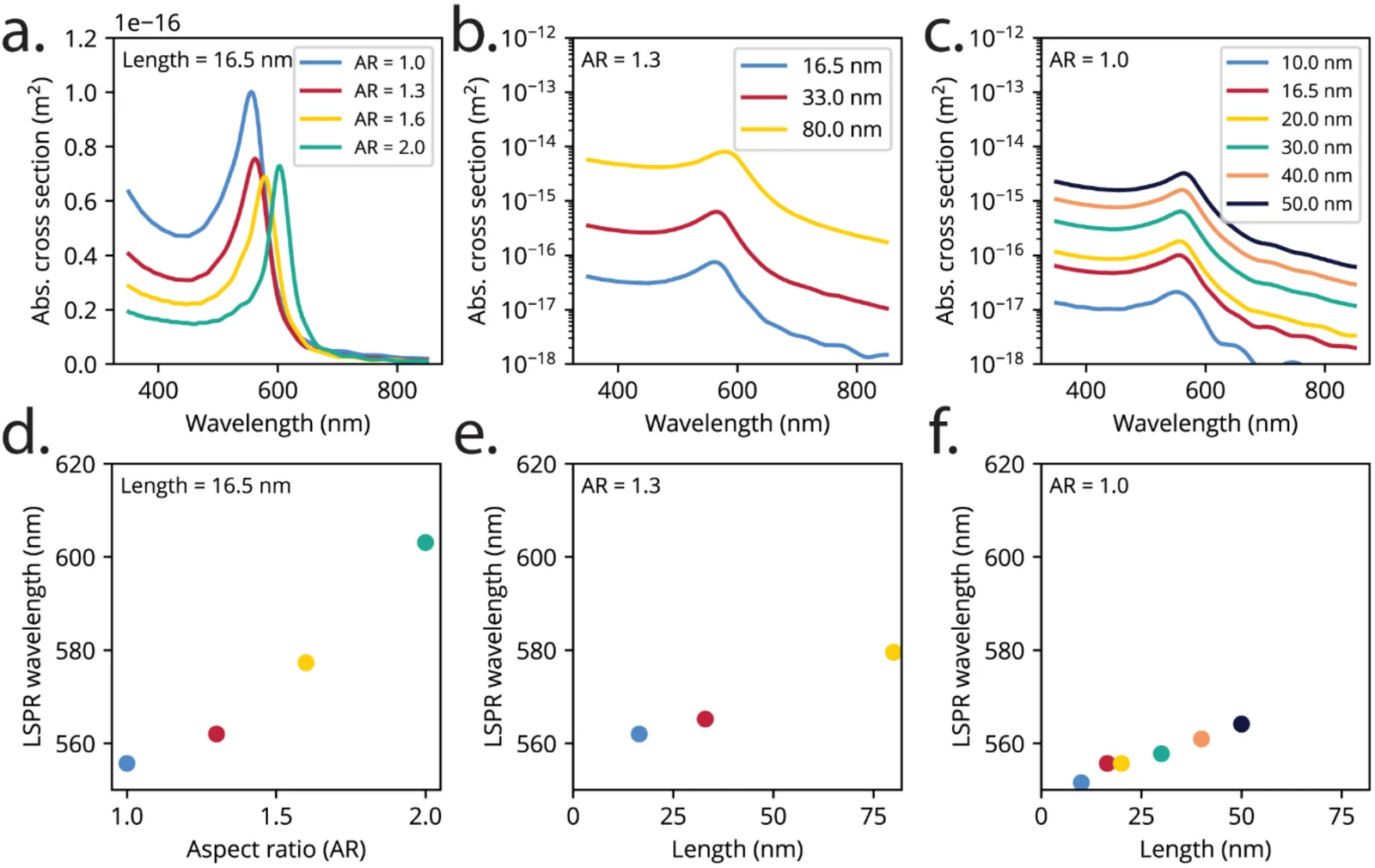
Simulated absorption cross section of Au nanoparticles (NPs) with various aspect ratios (AR) with a set particle length of 16.5 nm (a), spheres (AR = 1.0) with increasing size (b), and rods with AR of 1.3 with increasing size (c). Shift in main localized surface plasmon resonance (LSPR) position of simulated Au NPs with different AR (d), spheres with different sizes (e), and rods with different sizes (f).

To monitor the formation of the LSPR band during the photodeposition of the plasmonic Au NPs, we performed *in situ* UV–vis transmission spectroscopy, while introducing reactants and during the UV illumination (Figure S14 and Figure 1d–f, respectively), by measuring the transmission of a white light laser through the reaction mixture (see Figure S2 for a photo and schematic overview of the setup). To ensure sufficient transmission in these turbid solutions, we maximized the signal reaching the detector while maintaining a sufficiently high reactant concentration to observe changes in the LSPR signal. We used thin cuvettes (2 mm), focusing the white light beam through the cuvette and onto the detector, and low ZnO concentration (8 g/L), leading to transmission values of ∼4%. Through these efforts, we reached significant photon counts (>33% of maximum reachable counts) and improved the signal-to-noise ratio.

Additionally, the changing turbidity of the suspension due to the powder settling, stirring inconsistencies, and bubbling with N_2_ lead to drifts in intensity of the measured spectrum. To correct for these turbidity-related transmission drifts, we normalized the transmitted photon count at the LSPR peak to a region where Au light absorption is negligible (∼900 nm). We follow the LSPR wavelength that was derived from fitting the DR UV–vis spectroscopy measurements *i.e*., 547 nm for the reaction with ethanol and 512 nm for the reaction without a hole scavenger. We normalized to compare the *relative* differences between the photodeposition with and without ethanol and the irradiation of the ZnO suspension. Further control experiments with the same conditions, stirring, bubbling with N_2_, and UV illumination, were done with only water and only Au precursor solution (see Figure S15).

Before the start of the UV illumination, the suspensions were measured with *in situ* UV–vis transmission spectroscopy while introducing the reactants and removing oxygen, here called the equilibrium period. The normalized relative transmission during this equilibrium period decreased over time in all three cases (Figure S14). This decrease in relative transmission could arise either through increased absorption or enhanced reflection/scattering. By measuring the total reflectance of the suspensions at the different stages, *ex situ* using an integrating sphere, the scattering contribution was determined (Figures S16–S18). Changes in ZnO particle size, aggregation state, and suspension homogeneity during the reaction can affect the amount of light scattered out of the optical path. The *ex situ* reflectance measurements on the ZnO suspensions show that homogenization of the suspension from larger, unevenly dispersed ZnO clumps/aggregates to a more opaque and uniformly dispersed suspension causes a wavelength-dependent increase in reflectance above 385 nm (= the onset of the bandgap energy) (Figure S19). The wavelength-dependence is caused by Rayleigh and Mie scattering of the broad size distribution of ZnO (Figure S20). This same wavelength-dependent increase was observed in cases containing gold as well (Figures S16–S18). After UV irradiation, the spectral shape changes to show a distinctive dip related to the Au NP’s LSPR (Figures S16–S18). Since the LSPR is not visible in the measurements directly after the equilibrium step, we can confirm that the decrease in relative transmission during the equilibrium period is related to scattering changes due to homogenization of the ZnO rather than absorption due to Au NP formation. This scattering-related shift observed in the *ex situ* reflectance measurements and the decrease in normalized transmission in the *in situ* measurements reach a plateau within the 30 min equilibrium period. Therefore, we conclude that the wavelength-dependent scattering due to homogenization had stabilized before the start of the UV illumination.

After the equilibrium was reached, the UV light was switched on (at time = 0 s). Notably, upon UV illumination the relative transmission *increases* exponentially in the reactions where both ZnO and Au precursor are present, whereas the relative transmission in the ZnO suspension remains constant (Figure 1d). The increase in relative transmission occurs for 3 s when a hole scavenger is added compared to 35 s when no scavenger is added. On top of that, the slope of the increase is much steeper with hole scavenger. Additionally, the relative transmission in the ethanol case increases with ∼33% relative to directly before UV exposure, compared to ∼150% when no hole scavenger is added. This increase in transmission, including the shorter and steeper increase in the hole scavenged reaction, was observed when the experiment was duplicated (Figure S21). We also observe an increase in relative transmission at higher and lower wavelengths (750 and 480 nm, Figure S22).

The increase in relative transmission occurs either because the reflectance or absorptance decreases or due to photoluminescence (PL) of ZnO. PL of ZnO as a contributing factor can be excluded since PL would also *increase* the relative transmission when the ZnO suspension is illuminated. The relative transmission of the illuminated ZnO, however, remains constant in our measurements. Based on the same observation, photo dissolution of ZnO can also be ruled out as a cause. The increase in relative transmission cannot be attributed to a reversal of the process that occurred during the equilibrium period, *i.e*., the aggregation of ZnO particles, as such a reversal would also manifest in the ZnO control. Therefore, the increase in relative transmission must be caused by a decrease in absorptance, reflectance or a combination of both due to Au-ZnO interactions.

At 480 nm, the increase is partly related to the reduction of the AuCl_4_^–^ precursor to metastable intermediate AuCl_2_^–^.^27–31^ AuCl_4_^–^ exhibits a strong light absorption in the UV region (200–400 nm), with a tail extending to ∼500 nm (Figure S23) resulting in its yellow color. AuCl_2_^–^ absorbs further in the UV and is transparent in the visible range. The reduction of yellow AuCl_4_^–^ to transparent AuCl_2_^–^ would cause a decrease in light absorption and thus an increase in relative transmission. The steeper increase in light absorption at 480 nm in the presence of ethanol confirms that the hole scavenger accelerates the AuCl_4_^–^ to AuCl_2_^–^ reduction (Figure S24). The increase in relative transmission at 750 nm, where Au species absorb very little, indicate that this increase in relative transmission must be also related to changes in scattering. However, the control experiment with only ZnO shows no change in transmission upon UV illumination at all wavelengths (Figure S22). Therefore, the scattering changes that are observed at 750 nm must be exclusively related to Au-ZnO interactions. At 512 and 547 nm, the increase may be related to both absorption and scattering decreasing. These findings confirm that the increase in relative transmission is related to both absorption and reflection decreases due to Au-ZnO interactions. A deconvolution of the absorption and scattering contributions to the *in situ* transmission signal would ideally require time-resolved diffuse reflectance spectroscopy in an integrating sphere combined with transmission data. Future work could benefit from including reflected light due to scattering during the *in situ* UV–vis transmission measurements.

The initial exponential growth in relative transmission transitions to a *reduction* in transmission after 3 s with a hole scavenger and 35 s without a hole scavenger. We relate this second process to the growth of the LSPR band *i.e.*, the reduction to Au(0), leading to absorption at the LSPR wavelength (thereby reducing transmission). The later onset of this decrease can be explained by the fact that the small Au NPs (<3 nm) do not yet show plasmon excitation characteristics.^33^ The onset of this change occurs much faster when ethanol was added, showing the much faster particles growth and/or nucleation in the presence of a hole scavenger (Figure 1d). The relative transmission remains constant after 35 s without a hole scavenger added, whereas the relative transmission decreases by 21% from 3 s to 5 min in the hole scavenged case (Figure 1e). Additionally, the total reflectance was measured *ex situ* after equilibrium but before UV illumination and 5 min after UV illumination (see Figures S16–S18). In both reactions, with and without hole scavenger, the reflectance decreases by ∼32%. Since the reflectance decreases by the same amount over 5 min, but the relative transmission decreases significantly more with the addition of a hole scavenger, the share of absorption must increase substantially when ethanol is added compared to when it is not. Over the course of 45 min, the relative transmission decreases by ∼56% *with* hole scavenger compared to ∼20% *without* (Figure 1f). These results show that the Au NP nucleation and growth is faster with the addition of a hole scavenger compared to when no hole scavenger is added.

The KM function spectra (Figure 1c) show a single LSPR band at 547 nm for the Au NPs grown with ethanol as hole scavenger, compared to two peaks for the nonscavenged case. Electromagnetic simulations were employed to evaluate the effects of Au NP size and aspect ratio (AR) *i.e.*, the length/width of the Au NP, on the LSPR of Au NPs on ZnO and to disentangle the possible causes for an LSPR band with two peaks. Several causes could lead to a split band, as the position of the LSPR peak is dependent on the Au NP size, shape or surrounding. Near-field plasmonic coupling between neighboring Au nanoparticles can also red or blue shift the LSPR, depending on the orientation of the coupling.^26^ Another explanation could be that the additional peak corresponds to distinct plasmon modes (transversal and longitudinal) in anisotropic Au NPs with higher ARs. We simulated the absorption cross section of seven Au rods and seven Au spheres truncated on ZnO with increasing length or AR, as carried out with the Lumerical software. The truncation of the Au NP on the ZnO surface was estimated to be 20% based on TEM analysis of a representative Au NP at the edge of a ZnO particle in our Au/ZnO sample prepared without a hole scavenger, see Figure S6. The chosen dimensions of the simulated NPs were based on the size distribution of the Au NPs prepared without a hole scavenger (Figure S5). The influence of the AR value on the LSPR absorption was investigated by simulating Au NPs with a length of 16.5 nm, where the AR was increased from 1.0 to 1.3, 1.6, and 2.0 (Figure 2a). Increasing the AR value from 1.0 to 2.0 leads to a significant redshift of the LSPR peak (Figure 2d). The increase in AR produces a sharper, more defined band with a smaller full width at half-maximum. Additionally, as the AR value decreases, the simulated absorption spectra develop a higher tail at shorter wavelengths. For an AR value of 2.0, the LSPR band has a weak shoulder at a lower wavelength. This feature results from the separation of the longitudinal (oscillation along the long axis of the NP) and transverse (oscillation along the radial direction) modes in the Au NP as the particle becomes more anisotropic. Figure S25 shows the calculations of the simulated Au NPs under both polarizations, revealing the transversal and longitudinal modes in the Au NPs. The influence of particle size of rods was evaluated by simulating three Au rods with increasing length (*i.e*., 16.5, 33, and 80 nm) while the AR remained at a constant value of 1.3 (the mean of experimental particle size distribution) (Figure 2b). A large increase in absorption cross section, note the *y*-axis is in logarithmic scale, is observed when the size of rods with AR = 1.3 increases from 16.5 to 33 and 80 nm, causing a smaller redshift of the LSPR peak position (Figure 2e). A large increase in simulated absorption cross section is also observed when the particle size of spheres increases from 5 to 50 nm (Figure 2c). The red-shift caused by the size increasing of spheres­(Figure 2f) is, however, much less pronounced compared to the shift caused by the aspect ratio (Figure 2d). These results show that an increase in particle size (<50 nm), mainly influences the amplitude of the absorption cross section whereas the peak shift is mainly caused by increasing AR. Furthermore, the additional peak at ∼512 nm observed in the scavenger-free samples is unlikely to arise from the transverse modal contribution in the more anisotropic particles with AR approaching 2.0, as the transverse mode is weak compared to the longitudinal mode. The trends in these simulation results help to interpret the DR UV–vis spectra of the Au/ZnO samples under study.

We performed transmission electron microscopy (TEM) to gain insight into the morphology (*i.e*., size, shape, and size distribution) of the photodeposited Au NPs on ZnO: structural features that directly determine the optical response of the resulting Au/ZnO materials. These images were used to corroborate whether the double peak in DR UV–vis could relate to the morphological heterogeneity as found through our electromagnetic simulations. Figure 3 shows TEM images of the Au/ZnO hybrids prepared with ethanol as a hole scavenger (Figure 3a) and without a hole scavenger (Figure 3b). Since Au has a higher atomic number (*Z* = 79) than zinc (*Z* = 30), Au has more contrast than ZnO in the TEM images.^34^ However, transmission of electrons also depends on the thickness of a crystal and the diffraction conditions, *e.g.*, different crystal orientations.^34^ Due to the latter two interactions, the multisized ZnO NPs have a range of contrast (Figure S26 for the TEM images of ZnO and Figure S20 for size distribution). In the TEM image of Au/ZnO prepared with a hole scavenger (Figure 1a), the Au NPs are easily distinguishable from ZnO. The Au NPs appear as slightly elongated black particles on larger ZnO crystals. In the Au/ZnO sample prepared without a hole scavenger (Figure 3b), the Au NPs are not easily distinguishable from the ZnO particles due to a similar contrast of the big Au NPs and ZnO particles. STEM-EDX analysis allows to differentiate Au NPs from the ZnO crystals, as shown in the elemental map of Au, shown in Figure 3c (additional images are provided in Figures S27 and 28).

**Figure 3:**
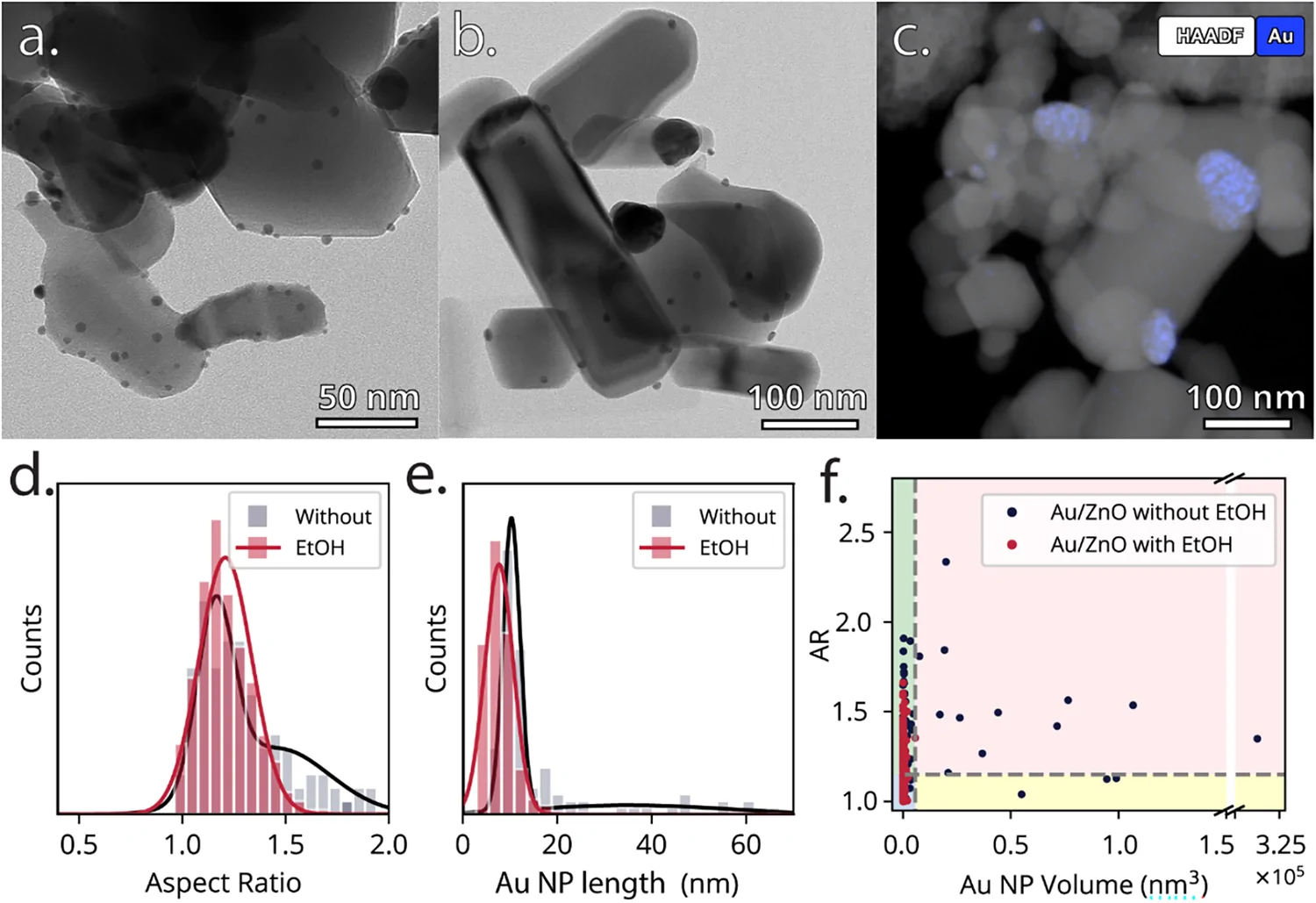
Transmission electron microscopy (TEM) images of Au/ZnO samples synthesized with ethanol (a) as hole scavenger and without a hole scavenger (b). Scanning transmission electron microscopy-energy dispersive X-ray (STEM-EDX) image of Au/ZnO sample synthesized without a hole scavenger (c). Mean aspect ratio (AR, length/width) (d) and mean length of the Au nanoparticles (NPs) (e). The sizes of the Au NPs were manually determined using the software ImageJ. Multiple locations in the grid were imaged. Only images with a magnification of 150k and higher were used for statistics. At least 108 Au NPs were measured in the images of Au/ZnO prepared without a hole scavenger and 230 Au NPs across 3 Au/ZnO batches prepared with ethanol were used. Au NP volume as a function of their aspect ratio (AR) for samples prepared without a hole scavenger and with ethanol as a hole scavenger (f). By approximating the rod-shaped particle as the sum of a sphere and a cylinder, the volume of each Au NP was calculated. Small spheres are categorized as Au NPs with a length < 30 nm and AR < 1.15 (light blue area), small rods with a length < 30 nm and AR > 1.15 (green area), large Au rods with a length > 30 nm and AR > 1.15 (pink area) and large Au spheres with length > 30 nm and AR < 1.15 (yellow area).

XPS was used to probe the oxidation state of the Au NPs for all preparation conditions (Figure S29 and Table S1). For all samples, the used fit reproduced the data with a Root Mean Square Deviation (RMSE) of <1.1%, indicating that any Au^3+^ or Au^1+^ contributions are at most trace-levels. This shows that the Au NPs are fully reduced. To confirm this finding, high-resolution TEM was performed on selected Au NPs of each preparation method (Figure S30 and Table S2). The measured lattice *d*-spacings match the expected values for metallic Au, confirming that the Au NPs are metallic in nature.

Another observation when comparing samples with and without a hole scavenger is the density of Au NPs on the surface of the ZnO particles. The average number of Au NPs on a ZnO particle is ∼32 when Au/ZnO is prepared with a hole scavenger, compared to ∼2 without. The low density of NPs when the reaction is performed without a hole scavenger is in line with previous literature.^35^ Nucleation and growth require photogenerated electrons to migrate to the ZnO surface; consequently, the nucleation and growth rate are influenced by both the number of photoexcited electrons and the rate of their migration. Hole scavengers suppress electron–hole recombination by extracting the holes. The gold weight loading of the samples prepared with and without ethanol were 0.9 wt % on ZnO as measured with ICP-OES (Table S3). Therefore, the higher number of nucleation sites but equal weight loading in the presence of a hole scavenger confirm that a larger amount of photogenerated electrons is available for Au-ion reduction when a hole scavenger is added.^3^

Figure 3d shows the AR, which provides insights into the anisotropy NPs. In the TEM images, it can be noted that the shapes of the Au NPs range from smaller spherical to slightly elongated to larger nonuniform particles. Plotting the AR for each condition confirms that the Au NPs range from spherical to elongated shapes. Size and AR distribution histograms for the Au NPs, including Gaussian fitting where possible, can be found in the SI (Figures S31–S33). Quantitative analysis of particle morphology reveals a bimodal distribution in the AR for the Au NPs without a hole scavenger, with a mean at 1.2 and 1.5. Compared to a single distribution for the Au NPs obtained via syntheses with a hole scavenger (AR ∼ 1.2 ± 0.2). On top of that, the mean particle length (Figure 3e) shows a clear and statistically significant difference. Due to the spatial resolution limitations of the Talos L120C TEM (120 kV) instrument, Au NPs below ∼2–3 nm may be underrepresented in the data set. It is, however, apparent that the Au/ZnO sample prepared *without* a hole scavenger results in the deposition of a large variety of Au NP sizes. A large population of smaller particles (μ = 10 nm) is observed, as well as a large spread in a few larger particles (μ = 53.1 nm and σ = 21.8 nm). The Au NP lengths range from ∼5.4 to ∼98.8 nm, and additional large nonuniform shapes were observed. The NP size distribution plot does not show a clear bimodal distribution in the nonscavenged synthesis, which is what we would expect based on the DR UV–vis spectroscopy and optical simulations. We attribute this to the relatively small population (108 NPs) compared to the bulk DR UV–vis measurement that probes ∼10^10^ more NPs. We do observe a significant dispersion in particle sizes and AR, which indicate that photodeposition without a hole scavenger leads to poor control over nucleation and growth during photodeposition without a hole scavenger, consistent with earlier literature reports on the photodeposition of Au on ZnO without a hole scavenger.^35^ This is further confirmed by plotting the width as a function of the length of the Au NP, through which we can investigate the Au NP shape growth *i.e.*, how the shape changes with size (Figure S34). Without the addition of a hole scavenger, the smaller particles (<30 nm length) lie close to the average aspect ratio, but the larger particles deviate more from the mean in both directions, indicating the lack of control as the NP grow larger than 30 nm. On the other hand, we find monodisperse Au NPs sizes of 7.5 ± 3.0 nm by 6.5 ± 2.6 nm for the Au/ZnO samples prepared *with* ethanol as a hole scavenger. The growth of the NPs is much more controlled, as there is little deviation from the mean AR.

The volume of each Au NP was calculated and plotted as a function of the AR (Figure 3f and for number and volume fractions see Table S4). We categorized four species: small spheres (length < 30 nm, AR < 1.15), small rods (length < 30 nm, AR < 1.15), large spheres (length > 30 nm, AR < 1.15), and large rods (length > 30 nm, AR > 1.15). In the Au/ZnO sample prepared without a hole scavenger, all four species are observed. Where there are a lot of small spheres (number fraction = 26. 9%) and a few large spheres (number fraction = 2.8%) leading to a volume fraction of 1.8 and 23.0%, respectively. Small and large rods make up 4.9 and 70.3 vol %, respectively. Au/ZnO prepared with lower weight loadings have a similar distribution, albeit with a large fraction of small particles indicating that a higher weight loading results in a clearer bimodal distribution of small versus large particles (Table S4). On the other hand, the Au NPs in the sample prepared with ethanol only lie in the small spheres or rods particles category (43.8 and 56.2 vol %, respectively). As seen in the simulated absorption spectra, increasing size in spheres mainly increases the absorption cross section where an increase in AR in rods can cause a large shift in LSPR wavelength. Both the large-rod and large-sphere populations carry substantial volume in the nonscavenged sample; each would contribute significantly to the overall absorption spectrum. To assess the contribution of each population, ensemble absorption spectra were reconstructed directly from the TEM-derived particle distribution through size distribution weighting (for calculations see page S29 and Figure S35). The reconstruction shows that the sphere-like and rod-like particle populations contribute at distinct spectral regions, where the sphere-like contribution is blue-shifted relative to the rod-like contribution (Figure S35e,f). The sample prepared without a hole scavenger contains a larger relative contribution from sphere-like particles, increasing the short-wavelength contribution to the reconstructed spectrum. On top of that, the peak difference in rod-like particles and sphere-like particles is bigger in the case without ethanol (Δ = 0.06) compared to with ethanol (Δ = 0.04), as the largest rods have a higher AR (2.3) compared to Au NPs prepared with ethanol (1.7) (Figure S35d). The peak splitting in DR UV–vis is therefore attributed to the presence of two distinct particle shape populations, with the greater abundance of large, high-aspect-ratio rods in the Au/ZnO sample prepared without a hole scavenger resulting in a more pronounced spectral separation.

However, the TEM-derived reconstruction does not reproduce the two experimentally resolved peaks at ∼512 and 623 nm. In the simulations, the separation between the sphere-like and rod-like resonances is smaller, and the magnitude of the sphere-like contribution is insufficient to produce a distinct second maximum. Both reconstructed spectra are also red-shifted relative to the corresponding experimental spectra. This reconstruction therefore supports the association of the shorter-wavelength contribution to the more sphere-like particle population but indicates that particle size and aspect ratio alone are insufficient to quantitatively reproduce the experimental bimodal DR UV–vis spectrum.

A possible reason could be that the simulated absorption cross sections were isolated-particle approximations. In areas with denser Au NP populations, near-field coupling can occur, which could cause spectral shifts as well. To assess the possible influence of this effect, the interparticle separations normalized to the mean equivalent diameter (D) of the two neighboring particles for samples prepared with and without ethanol were quantitatively analyzed (see page S31 for calculations). In the present samples, the median *g*/*D* values (1.47 with EtOH and 2.81 without EtOH) indicate that the majority of particle pairs lie outside the range in which strong coupling-induced spectral shifts are typically observed.^25^ Although near-field coupling is expected to be more relevant for the EtOH sample due to the smaller absolute and normalized interparticle gaps, the broad distributions of particle separations, sizes, and relative orientations are expected to result in a heterogeneous distribution of coupling interactions rather than a uniform spectral shift. Additional effects not included in the isolated-particle model may contribute to the experimental peak positions and separation, including uncertainty in the local refractive index of ZnO, differences in particle truncation onto the ZnO support, variations in the local dielectric environment, near-field interactions between neighboring particles and the finite statistical sampling of the particle population by TEM.

The combination of the ensemble measurements (UV–vis), trends in the electromagnetic simulations and local TEM image inspection (few nm^2^) collectively indicate that photodeposition without a hole scavenger leads to poor control over nucleation and growth. And on the other hand, the addition of a hole scavenger during the photodeposition of Au on ZnO increases the number of photogenerated electrons available for Au reduction, leading to mono disperse, slightly elongated Au NPs on the ZnO surface.

To investigate whether the observed effects are specific to ethanol or represent a general phenomenon for alcohol-based hole scavengers, we compared four different hole scavengers that are often used during photodeposition of Au on ZnO. An overview of published literature on the photodeposition of Au on ZnO can be found in the Supporting Information (Table S5), showing the uses of various hole scavengers and reaction conditions and the resulting particle size and wavelength at the LSPR. We selected four alcohols with increasing carbon number, as larger alcohols oxidize faster and consequently facilitate Au reduction faster.^8^ To ensure reproducible results, each reaction was performed at least two or three times.

In Figure 4a–d, TEM images of the Au NPs on ZnO prepared with methanol (a), ethanol (b), *iso-*propanol (c), or *tert*-butanol (d) are shown (more TEM images can be found in Figures S36–S39). In all cases, the Au NPs are distinguishable as slightly elongated black particles distributed on larger ZnO crystals. Particle sizes, shapes, and the distribution across the ZnO crystals appear similar despite the difference in molecular structure of the hole scavenger. To quantify these morphological similarities, the length (long axis) and width (diameter) were measured of ∼100–120 Au NPs per Au/ZnO sample across multiple regions on the grid, which combines to >230 Au NPs per hole scavenger since each experiment was performed multiple times (Figure 4e). We find monodisperse Au NPs sizes of 8.0 ± 3 nm (MeOH), 7.5 ± 3 nm (EtOH), 8.7 ± 3 nm (*i*-PrOH), and 9.9 ± 3 nm (*t*-BuOH). The small standard deviations confirm the uniformity of particle dimensions across all four alcohols. The AR is 1.2 ± 0.10 nm for Au NPs prepared with methanol, *iso*-propanol, and *tert*-butanol and 1.2 ± 0.15 nm for ethanol (Figure 4f). Additionally, lattice *d*-spacings and XPS analysis show that the preparation technique does not influence the final oxidation state of the Au (Figures S29 and S30, and Tables S1 and S2). The narrow and overlapping shape distributions confirm that the choice of hole scavenger does not significantly influence the Au NP shape. Size and AR distribution histograms for the Au NPs per condition, including Gaussian fitting where possible, can be found in the SI (Figures S40–S42). The batch-to-batch variation can be found in Table S6. The growth in all samples shows a linear growth relationship, which means that the Au NPs show a roughly constant AR across the population (Figure 4g, also see Figures S40–S42d). Similar slopes reveal a consistent correlation for all alcohols, reinforcing the idea that particle growth follows a similar mechanism regardless of the hole scavenger used. The similarity of morphology is further reflected in the DR UV–vis spectra (Figure 4h), which shows similarity in the Au LSPR bands. The DR UV–vis spectroscopy results of the individual experiments per hole scavenger are shown in Figure S43. The LSPR band of Au (see Figure S44), show almost identical shape and peaks between the Au/ZnO samples prepared under the same conditions. Fitting the Au LSPR band revealed nearly identical full widths at half-maximum (FWHM) (Figure 4i) and peak energies (Figure 4j) across the four alcohols, which indicates similar optical properties regardless of the choice of hole scavenger. Specifically, the LSPR peak energies were measured at 2.24 ± 0 eV (554 ± 0 nm) for methanol, 2.27 ± 0.01 eV (547 ± 1 nm) for ethanol, 2.27 ± 0.02 eV (547 ± 4 nm) for *iso*-propanol and 2.30 ± 0.01 eV (539 ± 2 nm) for *tert*-butanol. On top of that, the Au weight loading on ZnO was complete after 5 min of UV illumination for all cases, yielding 0.9 wt % Au/ZnO (Table S3). The minimal variation in all these values emphasizes the high reproducibility of the synthesis method and the uniformity of the resulting Au/ZnO hybrids.

**Figure 4:**
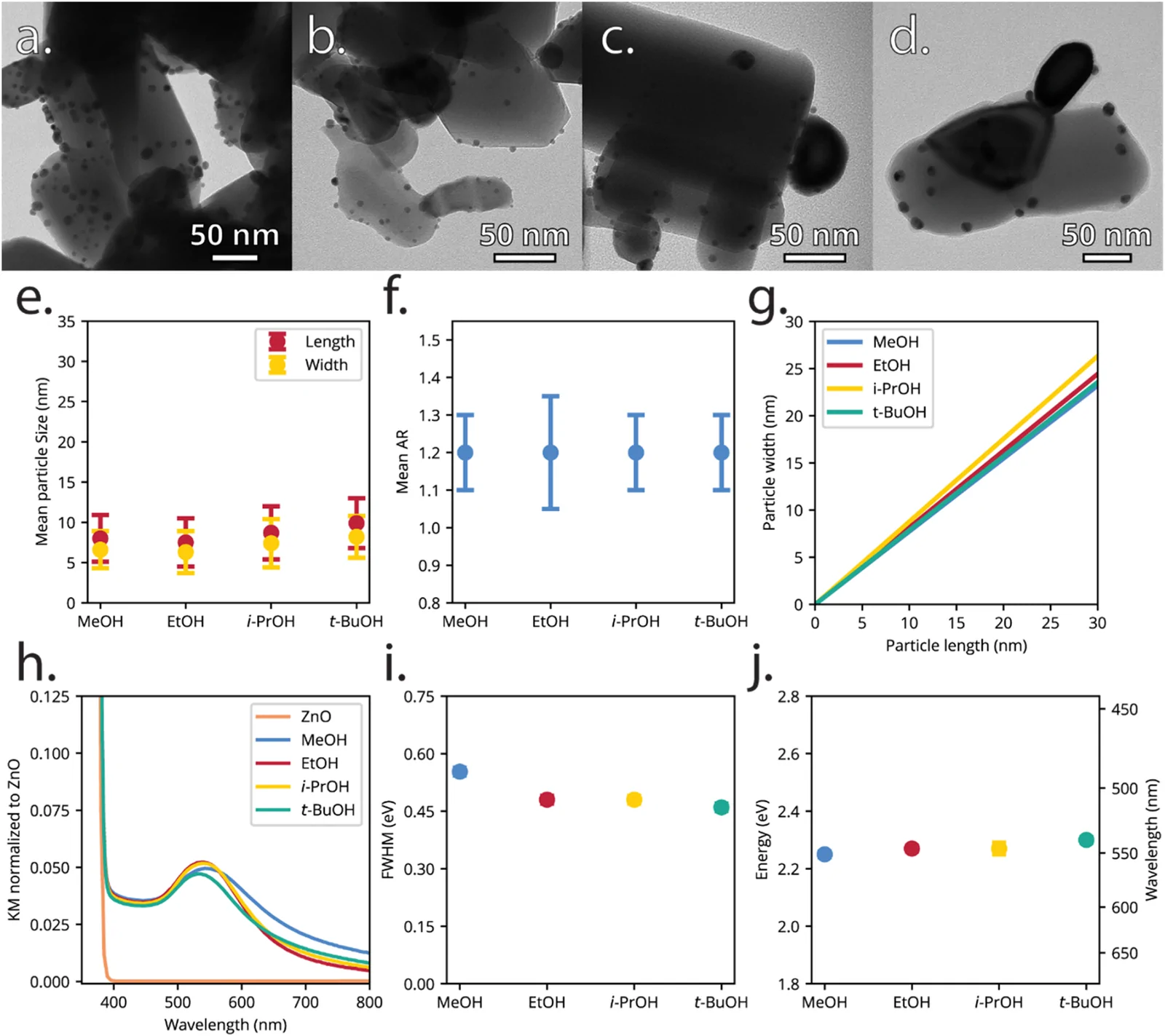
Transmission electron microscopy (TEM) images of Au/ZnO samples synthesized with methanol (a), ethanol (b), iso-propanol (c), and *tert*-butanol (d) as hole scavenger. Mean length and width of the Au nanoparticles (NPs) (e), mean aspect ratio (AR, length/width) (f), and the linear fit of the Au NP width as a function of length (g). Optical responses of Au/ZnO materials synthesized in the presence of the four hole scavengers. The values are derived from diffuse reflectance (DR) UV–vis spectroscopy measurements and converted into values proportional to absorption coefficient over the scattering coefficient via the Kubelka–Munk (KM) transformation and normalized to the bandgap absorption of ZnO (h). Full width at half-maximum (FWHM) (i) and energy at the peak (j) of the Au localized surface plasmon resonance (LSPR) band without normalization to ZnO per alcohol.

The oxidation potential, adsorption behavior, and reaction rate of methanol, ethanol, *iso*-propanol, and *tert*-butanol differ. However, the similarities in final morphology and optical response may be explained based on their oxidation rate versus their adsorption rate. Burek *et al.* found that larger alcohols are oxidized faster *i.e.*, their alcohol oxidation rate decreases in the following order *t*-butanol (*k*/*k*_MeOH_ = 303%) > *i*-propanol (*k*/*k*_MeOH_ = 182%) ≈ ethanol (*k*/*k*_MeOH_ = 214%) > methanol (*k*/*k*_MeOH_ = 100%) in the TiO_2_ catalyzed H_2_O_2_ formation.^8^ However, the amount of adsorption of the hole scavenger on the surface is also paramount to fast scavenging.^8^ Nagao and Morimoto found that alcohol molecules with a larger number of carbon atoms have decreased adsorption in the order of MeOH > EtOH > 1-PrOH on ZnO.^36^ Burek *et al.* relates this decreasing adsorption to the steric effects of the alcohols, where an increasing hydrocarbon chain length negatively affects their adsorption on hydrophilic surfaces (*e.g*., TiO_2_ and ZnO). This combination of adsorption limits and oxidation kinetics resulted in an initial reaction rate decreasing in the order *t*-butanol > 2-propanol = ethanol > methanol, but then due to adsorption limitations the order was reversed.^8^ The observed similarities in Au/ZnO morphology and optical response between the four alcohols can therefore be attributed to the balancing effects of oxidation kinetics and adsorption limitations over the course of the reaction. A systematic *in situ* kinetic comparison of all four alcohols remains an interesting direction for future work.

Our findings are summarized in the following scheme (Figure 5). These results show that in the presence of a hole scavenger, the photogenerated electrons cannot recombine with the already scavenged holes. The efficient charge separation generates a high density of photogenerated electrons. This leads to a fast reduction of Au^3+^ to Au^1+^, and subsequent reduction to Au(0) NPs on the ZnO surface. The high density of photogenerated electrons causes numerous nucleation sites leading to the formation of many small Au NPs with a uniform size and shape. In contrast, in the absence of a hole scavenger, fewer charge carriers are available, resulting in fewer nucleation sites. Subsequent electron transfer then favors the growth of a limited number of larger Au NPs. The limited number of photogenerated electrons results in an uncontrolled particle size and shape distribution.

**Figure 5:**
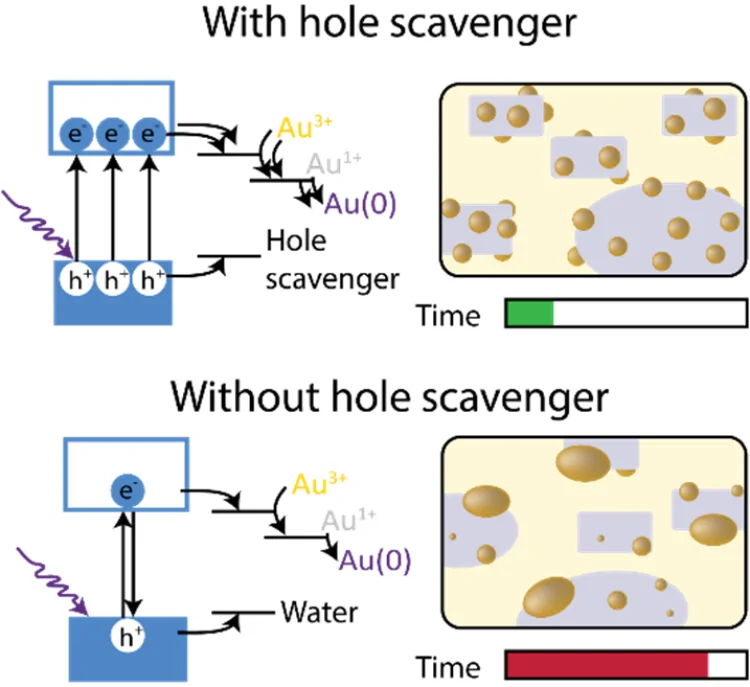
Schematic summary of the role of alcohol-based hole scavengers, such as ethanol, in Au nanoparticles (NPs) formation on a ZnO support.

## Conclusion

Although alcohol-based hole scavengers are widely used in the photodeposition of Au nanoparticles (NPs) on a ZnO support, a systematic evaluation of their influence on the growth kinetics and material properties is lacking. In this work, we investigated how the presence and nature of four alcohol-based hole scavengers influence the oxygen-deficient photodeposition of Au on ZnO by combining *in situ* UV–vis transmission spectroscopy, *ex situ* morphological characterization, and electromagnetic simulations. Our results demonstrate that the synthesis is highly reproducible, as confirmed by X-ray photoelectron spectroscopy, transmission electron microscopy and DR UV–vis spectroscopy. Unique *in situ* transmission UV–vis spectroscopy measurements revealed several processes that influenced the transmission through the suspension. Upon UV illumination, an exponential increase in relative transmission was observed that we relate to scattering of Au-ZnO interactions and the reduction of AuCl_4_^–^ to AuCl_2_^–^, followed by a process we relate to Au NPs formation. Importantly, the presence of ethanol as a hole scavenger leads to a faster nucleation and growth of Au NPs. In contrast, photodeposition in the absence of a hole scavenger resulted in fewer nucleation sites and a broad distribution of particle sizes and shapes. The corresponding DR UV–vis spectrum exhibited a split LSPR band with a primary peak at ∼623 nm and a shoulder at ∼512 nm. Electromagnetic reconstruction simulations confirm that sphere-like and rod-like contributions have distinct spectral regions, where rod-like particles are red-shifted relative to the sphere-like particle response. The peak splitting in DR UV–vis is therefore attributed to the presence of two distinct particle shape populations, with the greater abundance of large, high-aspect-ratio rods in the Au/ZnO sample prepared without a hole scavenger resulting in a more pronounced spectral separation. In contrast, the addition of ethanol as a hole scavenger significantly increased the number of nucleation sites, resulting in a monodisperse distribution of NP sizes and shapes. Notably, the use of any of the alcohol-based scavenger investigated, regardless of its chemical identity, yielded similar final Au NP sizes and distributions, with a consistent LSPR maximum at ∼547 nm. This study demonstrates the unique value of *in situ* UV–vis transmission spectroscopy in uncovering real-time dynamics that traditional *ex situ* methods cannot capture. As such, this methodology offers a widely applicable strategy for mechanistic studies of metal photodeposition on semiconductor supports beyond the specific Au/ZnO system studied here.

## Supplementary Material



## Data Availability

Data are available via https://doi.org/10.24416/UU01-SOZ4C7.

## Notes

Additional experimental details, materials, and characterization data for Au/ZnO, including UV–vis emission and absorbance spectra, TEM and STEM-EDX images, Au nanoparticle size distributions, Kubelka–Munk analyses, *in situ* UV–vis transmission spectroscopy, electromagnetic simulations, and band position diagrams (PDF)

## References

[1] Zhan F., Qi J., Li R., Zhao H., Liu Y., La P. (2025). Synthesis,
Photocatalytic Applications and Future Prospects of Noble Metal-modified
Semiconductors Fabricated via Photodeposition. Phys. Chem. Chem. Phys..

[2] Wenderich K., Mul G. (2016). Methods, Mechanism, and Applications of Photodeposition in Photocatalysis:
A Review. Chem. Rev..

[3] Chen G., Li R., Huang L. (2023). Advances in
Photochemical Deposition for Controllable
Synthesis of Heterogeneous Catalysts. Nanoscale.

[4] Carabineiro S. A. C., MacHado B. F., Bacsa R. R., Serp P., Draić G., Faria J. L., Figueiredo J. L. (2010). Catalytic
Performance of Au/ZnO Nanocatalysts
for CO Oxidation. J. Catal..

[5] Yan Y., Kobayashi H., Tada H., Soejima T. (2025). Site-Selective and
High-Density Gold Nanoparticle Photodeposition on the Edges of ZnO
Nanowires. J. Phys. Chem. Lett..

[6] Lu Q., Cui Q., Fang W., Li X., Zeng X., Shangguan W. (2024). *In-Situ* Photodeposition
of Gold Nanoparticles with Exposed High-Activity
Crystal Facets under Different Sacrificial Agents. Langmuir.

[7] Schneider J., Bahnemann D. W. (2013). Undesired Role of Sacrificial Reagents
in Photocatalysis. J. Phys. Chem. Lett..

[8] Burek B. O., Timm J., Bahnemann D. W., Bloh J. Z. (2019). Kinetic Effects
and Oxidation Pathways of Sacrificial Electron Donors on the Example
of the Photocatalytic Reduction of Molecular Oxygen to Hydrogen Peroxide
over Illuminated Titanium Dioxide. Catal. Today.

[9] Wenderich K., Han K., Mul G. (2018). The Effect
of Methanol on the Photodeposition of Pt
Nanoparticles on Tungsten Oxide. Part. Part.
Syst. Charact..

[10] Hamid S. B. A., Teh S. J., Lai C. W. (2017). Photocatalytic Water Oxidation on
ZnO: A Review. Catalysts.

[11] Yu H., Ming H., Zhang H., Li H., Pan K., Liu Y., Wang F., Gong J., Kang Z. (2012). Au/ZnO Nanocomposites:
Facile Fabrication and Enhanced Photocatalytic Activity for Degradation
of Benzene. Mater. Chem. Phys..

[12] Andrade G. R. S., Nascimento C. C., Silva Júnior E. C., Mendes D. T. S. L., Gimenez I. F. (2017). ZnO/Au Nanocatalysts for Enhanced
Decolorization of an Azo Dye Under Solar, UV-A and Dark Conditions. J. Alloys Compd..

[13] Castelló
Lux K., Hot J., Fau P., Bertron A., Kahn M. L., Ringot E., Fajerwerg K. (2023). Nano-gold Decorated ZnO: An Alternative
Photocatalyst Promising for NO_x_ Degradation. Chem. Eng. Sci..

[14] Yang B., Li C., Wang Z., Dai Q. (2022). Thermoplasmonics
in Solar Energy
Conversion: Materials, Nanostructured Designs, and Applications. Adv. Mater..

[15] Baffou G., Quidant R. (2013). Thermo-plasmonics: Using Metallic Nanostructures as
Nano-sources of Heat. Laser Photonics Rev..

[16] Baffou G., Quidant R., Girard C. (2009). Heat Generation
in Plasmonic Nanostructures:
Influence of Morphology. Appl. Phys. Lett..

[17] Mascaretti L., Naldoni A. (2020). Hot Electron and Thermal Effects in Plasmonic Photocatalysis. J. Appl. Phys..

[18] Han X. X., Rodriguez R. S., Haynes C. L., Ozaki Y., Zhao B. (2021). Surface-enhanced
Raman Spectroscopy. Nat. Rev. Methods Primers.

[19] Chanaewa A., Schmitt J., Meyns M., Volkmann M., Klinke C., Von Hauff E. (2015). Charge Redistribution and Extraction
in Photocatalytically
Synthesized Au–ZnO Nanohybrids. J. Phys.
Chem. C.

[20] Kavitha R., Kumar S. G. (2019). A Review on Plasmonic Au-ZnO Heterojunction Photocatalysts:
Preparation, Modifications and Related Charge Carrier Dynamics. Mater. Sci. Semicond. Process..

[21] Dong H.-D., Zhao J.-P., Peng M.-X., Zhang Y.-H., Xu P.-Y. (2023). Au-modified
Spindle ZnO for High Efficiency H_2_ Sensors. Vacuum.

[22] Kim H.-M., Hong Jeong D., Lee H.-Y., Park J.-H., Lee S.-K. (2019). Improved
Stability of Gold Nanoparticles on the Optical Fiber and Their Application
to Refractive Index Sensor Based on Localized Surface Plasmon Resonance. Optics Laser Technol..

[23] He W., Kim H. K., Wamer W. G., Melka D., Callahan J. H., Yin J. J. (2014). Photogenerated Charge
Carriers and Reactive Oxygen
Species in ZnO/Au Hybrid Nanostructures with Enhanced Photocatalytic
and Antibacterial Activity. J. Am. Chem. Soc..

[24] Wu M., Chen W. J., Shen Y. H., Huang F. Z., Li C. H., Li S. K. (2014). *In-Situ* Growth of Matchlike ZnO/Au Plasmonic Heterostructure
for Enhanced Photoelectrochemical Water Splitting. ACS Appl. Mater. Interfaces.

[25] Jain P. K., Huang W., El-Sayed M. A. (2007). On the
Universal Scaling Behavior
of the Distance Decay of Plasmon Coupling in Metal Nanoparticle Pairs:
A Plasmon Ruler Equation. Nano Lett..

[26] Rechberger W., Hohenau A., Leitner A., Krenn J. R., Lamprecht B., Aussenegg F. R. (2003). Optical
properties of two interacting gold nanoparticles. Opt. Commun..

[27] Fernando J. F. S., Shortell M. P., Noble C. J., Harmer J. R., Jaatinen E. A., Waclawik E. R. (2016). Controlling Au Photodeposition on
Large ZnO Nanoparticles. ACS Appl. Mater. Interfaces.

[28] McGilvray K. L., Granger J., Correia M., Banks J. T., Scaiano J. C. (2011). Opportunistic
Use of Tetrachloroaurate Photolysis in the Generation of Reductive
Species for the Production of Gold Nanostructures. Phys. Chem. Chem. Phys..

[29] Yoon S., Kim C., Lee B., Lee J. H. (2019). From a Precursor to an Etchant: Spontaneous
Inversion of the Role of Au­(III) chloride for One-pot Synthesis of
Smooth and Spherical Gold Nanoparticles. Nanoscale
Adv..

[30] Tsuji M., Miyamae N., Nishio M., Hikino S., Ishigami N. (2007). Shape Selective
Oxidative Etching and Growth of Single-Twin Plate-Like and Multiple-Twin
Decahedral and Icosahedral Gold Nanocrystals in the Presence of Au
Seeds under Microwave Heating. Bull. Chem. Soc.
Jpn..

[31] Gole A., Murphy C. J. (2004). Seed-Mediated Synthesis of Gold Nanorods: Role of the
Size and Nature of the Seed. Chem. Mater..

[32] Derkachova A., Kolwas K., Demchenko I. (2016). Dielectric
Function for Gold in Plasmonics
Applications: Size Dependence of Plasmon Resonance Frequencies and
Damping Rates for Nanospheres. Plasmonics.

[33] Hildebrandt J., Taubert A., Thunemann A. F. (2024). Synthesis
and Characterization of
Ultra-Small Gold Nanoparticles in the Ionic Liquid 1-Ethyl-3-methylimidazolium
Dicyanamide, [Emim]­[DCA]. ChemistryOpen.

[34] Williams, D. B. ; Carter, C. B. Transmission Electron Microscopy. In A Textbook for Materials Science, 2nd ed.; Springer E-Books; Springer, 2009 10.1007/978-0-387-76501-3.

[35] Cure J., Assi H., Cocq K., Marìn L., Fajerwerg K., Fau P., Bêche E., Chabal Y. J., Estève A., Rossi C. (2018). Controlled Growth and
Grafting of High-Density Au Nanoparticles on Zinc Oxide Thin Films
by Photo-Deposition. Langmuir.

[36] Nagao M., Morimoto T. (1980). Adsorption of Alcohols
on Zinc Oxide Surfaces. J. Phys. Chem. A.

