# Supplementary material for: In Situ UV–Vis Spectroscopy Insights into the Role of Hole Scavengers during the Photodeposition of Uniform Gold Nanoparticles on Zinc Oxide
Source: J Phys Chem C Nanomater Interfaces. 2026 Oct 10;130(42):14678–90. doi: 10.1021/acs.jpcc.6c03740 (PMC13700086; doi:10.1021/acs.jpcc.6c03740)
Supplement: Supplementary file 1 [file jp6c03740_si_001.pdf]

Supporting information to

In-Situ UV-Vis Spectroscopy Insights into the Role of Hole Scavengers during the Photodeposition of Uniform Gold Nanoparticles on Zinc Oxide

Roos L. Grote[1], Elaina Galvin[2], Nico van Mil[1], Ariq Attallah[3], Emiel J. M. Hensen[3], Erik C. Garnett[2], Bert M. Weckhuysen[1,*], Eline M. Hutter[1,*]

1. Institute for Sustainable and Circular Chemistry, Department of Chemistry, Faculty of Science, Utrecht University, Universiteitsweg 99, 3584 CG Utrecht, The Netherlands
2. AMOLF, Science Park 104, 1098 XG Amsterdam, The Netherlands
3. Laboratory of Inorganic Materials and Catalysis, Department of Chemical Engineering and Chemistry, Eindhoven University of Technology, Eindhoven, The Netherlands

List of Figures

Figure S1: Emission spectrum of the used UV source from DarkBeam with an irradiance of 33 mW/cm^2, as measured with a fiber coupled OceanOptics FLAME-T-VIS-NIR-ES instrument. 6
Figure S2: A photo of the *in-situ* UV-Vis transmission spectroscopy set-up showing the white light beam focused on a 2 mm pathlength cuvette that is stirred and purged with nitrogen, while illuminated with UV light (left). A schematic overview of the *in-situ* UV-Vis transmission spectroscopy set-up (right). 6
Figure S3: Emission spectrum of the white light source used for the *in-situ* UV-Vis spectroscopy experiments, as measured with a fiber coupled OceanOptics FLAME-T-VIS-NIR-ES instrument. 6
Figure S4: 8 g/L 0.7wt% Au/ZnO suspensions in a 2 mm path-length cuvette equipped with a magnetic stirrer before *in-situ* UV-Vis transmission spectroscopy (left), after 45 min reaction without hole scavenger added (middle), and with ethanol added as a hole scavenger (right).7
Figure S5: The aspect ratio (AR) as a function of the length of the Au nanoparticles (NPs) for non-hole scavenged Au/ZnO, including the sizes and AR that were chosen for electromagnetic simulations....................7
Figure S6: Transmission electron microscopy (TEM) image of Au/ZnO prepared without a hole scavenger. In-set shows the gold truncation of 20 % on to ZnO surface that was used as an estimation for electromagnetic simulations. 8
Figure S7: Kubelka-Munk (KM) function zoomed in on Au absorption band fitted with a linear baseline and two Gaussians for a control experiment with ethanol as a hole scavenger and flushing compressed air instead of nitrogen during photodeposition of Au on ZnO....................8
Figure S8: Aspect ratio, length, and width distributions, scattering plot of width as a function of length of Au/ZnO samples prepared with **oxygen** (compressed air) instead of N_2 purging.9
Figure S9: Au/ZnO prepared with oxygen during photodeposition taken with scanning transmission eletron-microscopy – energy dispersive X-ray (STEM-EDX) analysis (a),

elemental maps of Zn and Au (b), elemental map of Au (c), and Bright-field transmission electron microscopy (TEM) image (d). 9
Figure S10: Band positions of Au (Fermi-level of bulk Au is 5.1 eV) [1, 2], ZnO valence and conduction band edges[3] and various oxidation potentials of water oxidation and hole scavenger oxidations.[4] 10
Figure S11: Kubelka-Munk (KM) function zoomed in on Au absorption band fitted with a linear baseline and two Gaussians for each synthesis with ethanol as a hole scavenger during photodeposition of Au on ZnO. 12
Figure S12: Kubelka-Munk (KM) function zoomed in on Au absorption band fitted with a linear baseline and three Gaussians for each synthesis without a hole scavenger during photodeposition of Au on ZnO (synthesis 1 and 2 were prepared with 0.009 mmol $HAuCl_4$ which yielded 0.6 wt.% Au/ZnO and synthesis 3 was prepared with 0.012 mmol, which yielded 0.9 wt.% Au/ZnO). 13
Figure S13: Kubelka-Munk (KM) function of three Au/ZnO photodeposition without the addition of a hole scavenger with different weight loadings of gold (left) and a zoom of the Au band (right). Synthesis 1 and 2 were prepared with 0.009 mmol $HAuCl_4$ which yielded 0.6 wt.% Au/ZnO and synthesis 3 was prepared with 0.012 mmol, which yielded 0.9 wt.% Au/ZnO). 14
Figure S14: Normalized (to end of equilibrium (t = -0.4 s)) relative transmission at Localized Surface Plasmon Resonance (LSPR) (510 nm for Au/ZnO without ethanol, 547 nm for Au/ZnO with ethanol, and 510 for ZnO suspension) during the sequential stages of the equilibrium period, starting with the ZnO suspension, followed by the addition of Au precursor (yellow dashed line), ethanol (blue dashed line) and the start of N_2 bubbling (pink dashed line). 14
Figure S15: Relative transmission (intensity at 510 ± 10 nm over intensity at 900 ± 10 nm) during *in-situ* UV-Vis spectroscopy measurements of miliQ during UV exposure (left). Continued measurement through addition of Au precursor ($HAuCl_4.3H_2O$) at yellow dashed line, and UV exposure (purple dashed line) (right). 15
Figure S16: Total reflectance measured *ex-situ* at the exit of the integrating sphere without a back reflector at different steps within the photodeposition of Au on ZnO without a hole scavenger. Stirring was applied during the measurements. 15
Figure S17: Total reflectance measured ex-situ at the exit of the integrating sphere without a back reflector at different steps within the photodeposition of Au on ZnO with ethanol as a hole scavenger. Stirring was applied during the measurements. 16
Figure S18: Total reflectance measured *ex-situ* at the exit of the integrating sphere without a back reflector at different steps within the blank reaction with the ZnO suspension. Stirring was applied during the measurements. 16
Figure S19: Effect of stirring-induced homogenization on the total reflectance of a ZnO suspension. Seven consecutive total reflectance spectra of a ZnO suspension, recorded upon the onset of stirring. Photographs of the cuvette taken (left) within the first few seconds of stirring, showing larger, unevenly dispersed ZnO clumps/aggregates, and (right) after the equilibrium period, showing a visibly more opaque and uniformly dispersed suspension. 17
Figure S20: Particle size distribution of ZnO particles. 17
Figure S21: Normalized relative transmission at Localized Surface Plasmon Resonance (LSPR) during photo-deposition of Au on ZnO with and without ethanol as hole scavenger or the illumination of a ZnO suspension. In this experiment He was used instead of N_2 to remove O_2 from the suspension. This may not be the best gas to remove O_2 as it is very light, therefore later experiments were done with N_2 to imitate *ex-situ* syntheses. 17
Figure S22: Normalized relative transmission at different wavelengths during photodeposition of Au on ZnO with (left) and without ethanol (middle) as hole scavenger,

and illumination of a ZnO suspension (right). Lighter data points are all data points, whereas darker data points are a running average with a Δt = 10 s..18
Figure S23: Absorbance of Au precursor solution measured in 10 mm pathlength cuvette in transmission mode. 18
Figure S24: Normalized relative transmission at 480 nm during photodeposition of Au on ZnO with and without ethanol as hole scavenger, and illumination of a ZnO suspension. 18
Figure S25: Electromagnetic simulations with different polarization to excite the longitunidal and transverse mode for each Au nanoparticle (NP) (length, width, aspect ratio (AR) and the modeling input radii of the simulated Au NPs can be found in Table 1 in the main text). 19
Figure S26: Bright-field transmission electron Microscopy (TEM) images of bare ZnO. 20
Figure S27: Dark-field scanning transmission electron microscopy–energy dispersive X-ray (STEM-EDX) analysis images of Au/ZnO prepared without a hole scavenger **0.6 wt.% Au**; gold elemental map displayed in blue. 20
Figure S28: Dark-field scanning transmission electron microscopy–energy dispersive X-ray (STEM-EDX) analysis images of Au/ZnO prepared without a hole scavenger **0.9 wt.% Au**; gold elemental map displayed in blue. 20
Figure S29: X-ray photoelectron spectroscopy (XPS) data of the Au 4f binding energy region for Au/ZnO samples prepared with different hole scavengers added, without a hole scavenger present, and with oxygen. The Au $4f_{7/2}$, Au $4f_{5/2}$, Zn $3p_{3/2}$, and Zn $3p_{1/2}$ were fitted with four pseudo-Voigt profiles, using fixed spin-orbit splitting, fixed Full-Width at Half Maximum (FWHM) and fixed area ratios. The Root Mean Square Error (RMSE) of the total fit are displayed for each preparation condition. 21
Figure S30: High resolution-transmission electron microscopy (HR-TEM)/fast Fourier transform (FFT)-derived lattice d-spacings for Au/ZnO samples prepared with different conditions. Prepared with methanol (a, b), ethanol (c), *iso*-propanol (d), *tert*-butanol (e, f) as a hole scavenger, without a hole scavenger (g, h), and with oxygen bubbling during photodeposition (i, j). An overview of the d-spacings can be found in Table S2...................23
Figure S31: Aspect ratio, length, and width distributions, scattering plot of width as a function of length of Au/ZnO samples prepared with **ethanol** as hole scavenger..............................25
Figure S32: Aspect ratio, length, and width distributions, scattering plot of width as a function of length of Au/ZnO prepared **without** a hole scavenger with **0.6 wt.% Au/ZnO**.................25
Figure S33: Aspect ratio, length, and width distributions, scattering plot of width as a function of length of Au/ZnO prepared **without** a hole scavenger with **0.9 wt.% Au/ZnO**.................26
Figure S34: The relationship between Au nanoparticle (NP) width and length and their linear fit for Au/ZnO prepared with and without ethanol...26
Figure S35: Transmission electron microscopy (TEM)-weighted reconstruction of the simulated Au-ZnO nanoparticle absorption spectra from the experimentally determined particle distributions. TEM-derived particle length and width are shown for samples prepared (a) without EtOH and (b) with EtOH. Each measured particle is assigned to the closest simulated geometry based on aspect ratio and particle size. Colored circles represent experimentally measured particles, while crosses indicate the corresponding simulated particle geometries. (c) Normalized total TEM-weighted reconstructed absorption spectra for samples prepared with and without EtOH. (d) Normalized total reconstructed spectra together with the corresponding sphere-like and rod-like contributions. Each sample is normalized to the maximum of its total reconstructed spectrum, retaining the relative contributions of the sphere-like and rod-like populations. (e) Absolute sphere-like contribution to the reconstructed absorption, retaining the simulated absorption cross-sections and experimentally determined population weighting. (f) Absolute total TEM-weighted reconstructed absorption spectra. The sphere-like contribution is substantially larger for the sample prepared without EtOH, consistent with the greater abundance and optical contribution of sphere-like particles in this sample. The sphere-like contribution peaks

at 563 nm without EtOH and 553 nm with EtOH, while the total reconstructed spectra peak at 571 and 562 nm, respectively. 28
Figure S36: Transmission electron microscopy (TEM) images of Au/ZnO prepared with **methanol** as hole scavenger. 32
Figure S37: Transmission electron microscopy (TEM) images of Au/ZnO prepared with **ethanol** as hole scavenger. 32
Figure S38: Transmission electron microscopy (TEM) images of Au/ZnO prepared with ***iso*-propanol** as hole scavenger. 33
Figure S39: Transmission Electron Microscopy (TEM) images of Au/ZnO prepared with ***tert*-butanol** as hole scavenger. 33
Figure S40: Aspect ratio, length, and width distributions, scattering plot of width as a function of length of Au/ZnO samples prepared with **methanol** as hole scavenger. 33
Figure S41: Aspect ratio, length, and width distributions, scattering plot of width as a function of length of Au/ZnO samples prepared with ***iso*-propanol** as hole scavenger. 34
Figure S42: Aspect ratio, length, and width distributions, scattering plot of width as a function of length of Au/ZnO samples prepared with ***tert*-butanol** as hole scavenger. 34
Figure S43: Kubelka-Munk (K-M) function applied to Diffuse Reflectance (DR) UV-Vis spectroscopy results normalized to the ZnO absorption band of dried Au/ZnO samples photo-deposited with a) methanol, b) ethanol, c) *iso*-propanol, d) *tert*-butanol, and e) without a hole scavenger. 35
Figure S44: Kubelka-Munk (KM) function applied to Diffuse Reflectance (DR) UV-Vis spectroscopy results normalized to the Au absorption band of dried Au/ZnO samples photo-deposited with a) methanol, b) ethanol, c) iso-propanol, d) tert-butanol, and e) without a hole scavenger. 36

List of Tables

Table S1: Binding energies (BE) of the fitted Au 4f and Zn 3p components (as shown in Figure S29). 22
Table S2: High resolution-transmission electron microscopy (HR-TEM)/fast Fourier transform (FFT)-derived lattice d-spacings for Au/ZnO samples with different hole scavengers added, without a hole scavenger present, and with oxygen. Compared to reference values from PDF5 cards for Au (00-004-0784), A_2O_3 (00-043-1039), and ZnO (Wurtzite, 00-036-1451). 24
Table S3: Gold weight loading photodeposited on ZnO with different hole scavengers added, without a hole scavenger present, and with oxygen. 25
Table S4: Gold nanoparticle (Au NP) number and volume fractions for samples prepared with and without a hole scavenger (0.6 wt.% Au and 0.9 wt.% Au). By approximating the rod-shaped particle as the sum of a sphere and a cylinder. Small spheres are categorized as Au NP with a length < 30nm and AR < 1.15, small rods with length < 30 nm and AR > 1.15, large Au rods with a length > 30 nm and AR > 1.15 and large Au spheres with length > 30 nm and AR < 1.15. 26
Table S5: Overview of the particle sizes and localized surface plasmon resonance (LSPR) of photo-deposited Au/ZnO in the open literature, including the conditions, light source, irradiation time, used solvent and hole scavenger as well as the research domain and application of the materials made. NA = Not available. PD = Photodeposition. 30
Table S6: Batch-to-batch reproducibility of Au nanoparticle dimensions for each preparation condition (with different hole scavengers added, without a hole scavenger present, and with oxygen conditions), determined by transmission electron microscopy (TEM). For each hole scavenger, the reaction was performed in 2–3 independent batches. Values are reported as mean ± standard deviation per batch. N denotes the number of particles analyzed. The combined statistics for each hole scavenger (bold rows), correspond to the combined dataset shown in Figure 4e-g and histograms shown in Figure S29-30. 35

List of equations

S. 1.	ZnO bandgap excitation	$ZnO + h\nu \rightarrow h^+ + e^-$
S. 2.	Gold precursor reduction	$AuCl_4^- + 3\ e^- \rightarrow Au(0) + 4\ Cl^-$
S. 3.	Hydrogen formation	$2\ H^+ + 2\ e^- \rightarrow H_2$
S. 4.	Oxygen reduction	$O_2 + 4\ e^- + 4\ H^+ \rightarrow 2\ H_2O$
S. 5.	Oxygen evolution reaction	$2\ H_2O(l) + 4\ h^+ \rightarrow O_2(g) + 4\ H^+\ (aq) + 4e^-$
S. 6.	Methanol oxidation	$CH_3OH_{(ads)} + H_2O \rightarrow CO_2 + 6\ H^+ + 6\ e^-$
S. 7.	Ethanol oxidation	$C_2H_5OH_{(ads)} + 3\ H_2O \rightarrow 2\ CO_2 + 12\ H^+ + 12\ e^-$
S. 8.	*Iso*-propanol oxidation	$C_3H_7OH_{(ads)} + 5\ H_2O \rightarrow 3\ CO_2 + 18\ H^+ + 19\ e^-$
S. 9.	*Tert*-butanol oxidation	$C_4H_9OH_{(ads)} + 7\ H_2O \rightarrow 4\ CO_2 + 24\ H^+ + 24\ e^-$
S. 10.	Photo-corrosion of ZnO	$ZnO\ (s) + 2\ h^+ + H_2O \rightarrow Zn^{2+}(aq) + ½\ O_2 + 2H^+$
S. 11.	Photolysis of Gold step 1	$AuCl_4^- + h\nu \rightarrow AuCl_3^- + Cl\ \cdot$
S. 12.	Photolysis of Gold step 2	$2\ AuCl_3^- \rightarrow AuCl_4^- + AuCl_2^-$
S. 13.	Photolysis of Gold step 3	$2\ AuCl_2^- \rightarrow Au(0) + AuCl_4^-$
S. 14.	Absorption weighted contribution	$A_{ensemble}(\lambda) = \sum_i w_i A_i(\lambda)$
S. 15.	Equivalent diameter	$D_{\mathrm{eq}} = \sqrt{LW}$
S. 16.	Mean equivalent diameter	$D = \frac{D_{\mathrm{eq},1}+D_{\mathrm{eq},2}}{2} = \frac{\sqrt{L_1W_1}+\sqrt{L_2W_2}}{2}$
S. 17.	Normalized interparticle separation	$\frac{g}{D} = \frac{2g}{\sqrt{L_1W_1}+\sqrt{L_2W_2}}$

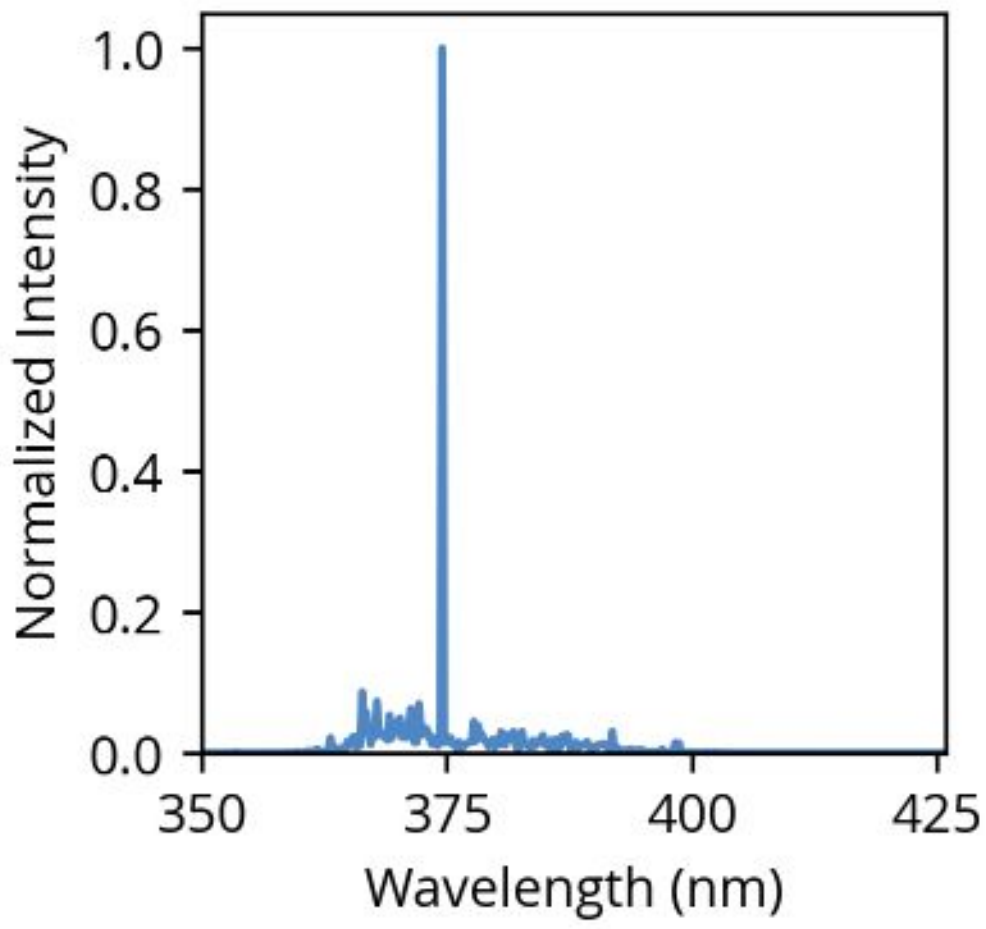

Figure S1: Emission spectrum of the used UV source from DarkBeam with an irradiance of 33 mW/cm^2, as measured with a fiber coupled OceanOptics FLAME-T-VIS-NIR-ES instrument.

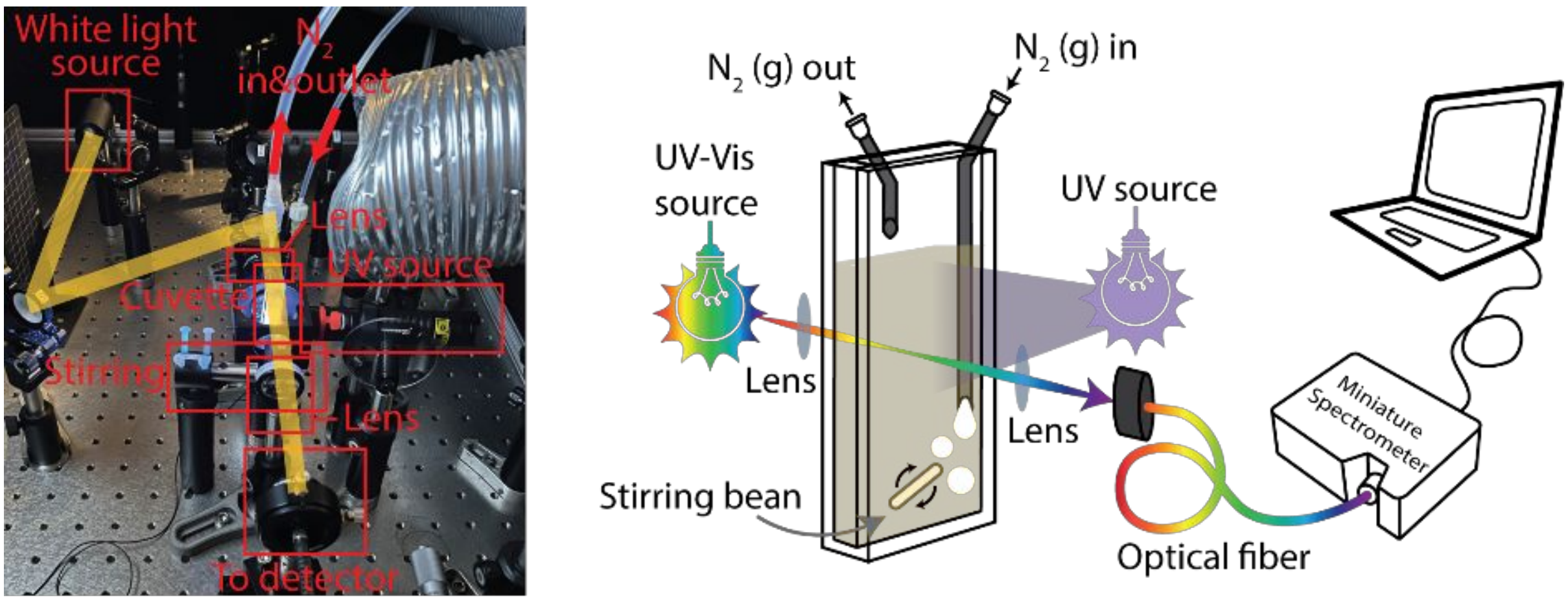

Figure S2: A photo of the *in-situ* UV-Vis transmission spectroscopy set-up showing the white light beam focused on a 2 mm pathlength cuvette that is stirred and purged with nitrogen, while illuminated with UV light (left). A schematic overview of the *in-situ* UV-Vis transmission spectroscopy set-up (right).

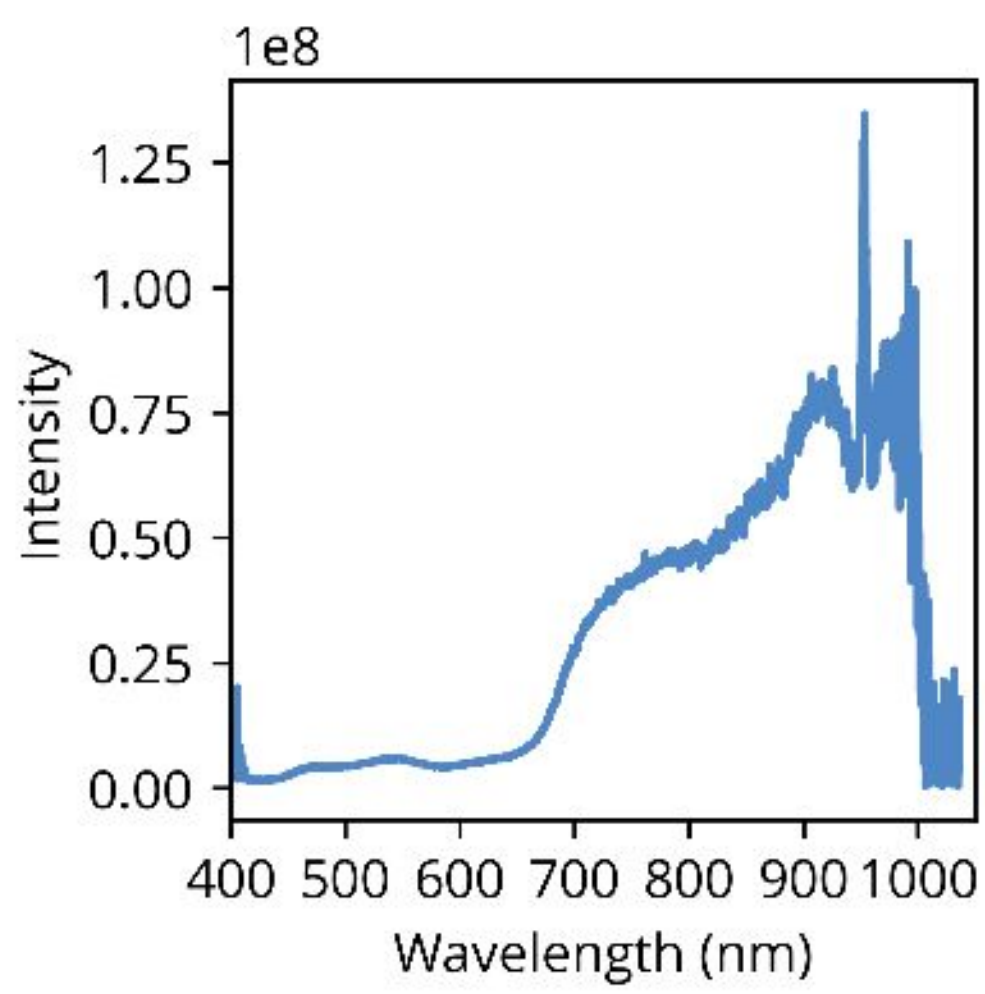

Figure S3: Emission spectrum of the white light source used for the *in-situ* UV-Vis spectroscopy experiments, as measured with a fiber coupled OceanOptics FLAME-T-VIS-NIR-ES instrument.

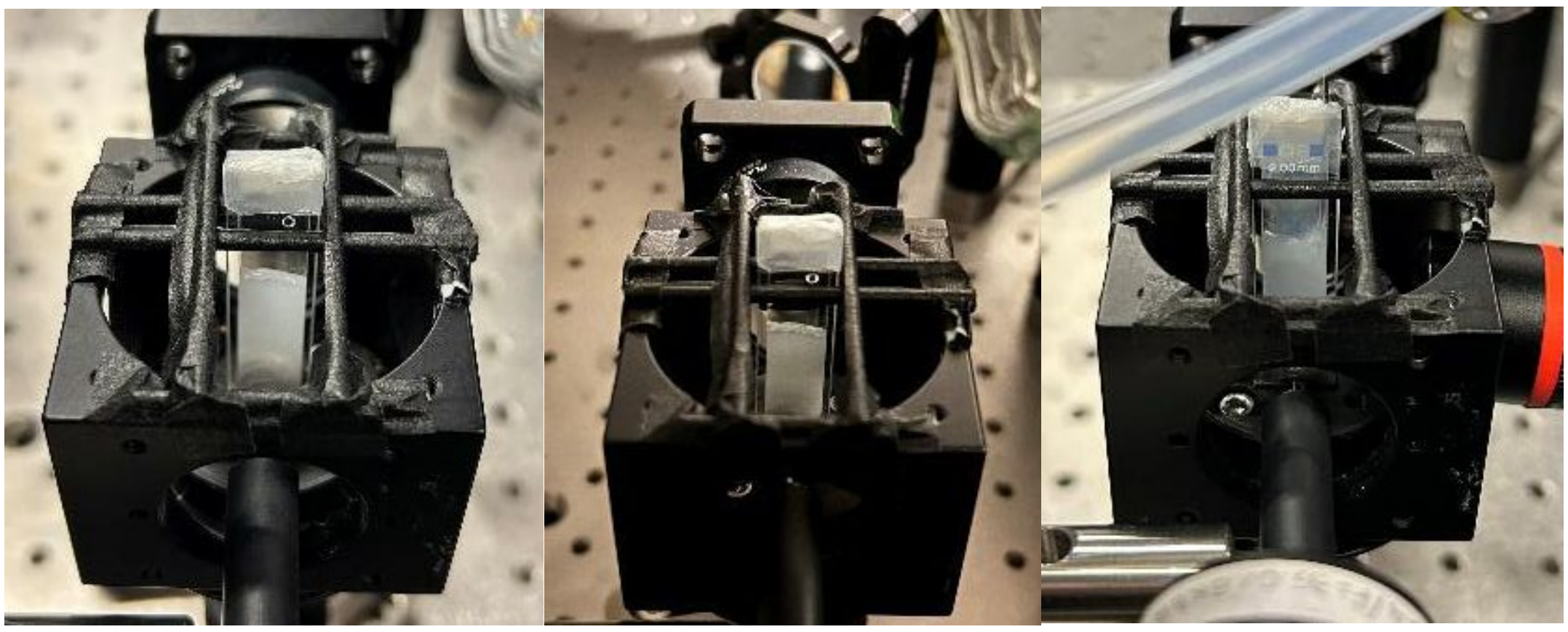

Figure S4: 8 g/L 0.7wt% Au/ZnO suspensions in a 2 mm path-length cuvette equipped with a magnetic stirrer before *in-situ* UV-Vis transmission spectroscopy (left), after 45 min reaction without hole scavenger added (middle), and with ethanol added as a hole scavenger (right).

To estimate evaporative loss during the longest reaction studied (45 min), the change in liquid level was determined from photographs taken before and after the reaction, yielding an estimated volume loss of ~40 µL from an initial 500 µL (~ 8 %). Given the large molar excess of hole scavenger used (~4800:1 relative to Au), even if this entire volume loss is conservatively attributed to alcohol evaporation, the hole scavenger would remain in ~4400-fold molar excess relative to Au throughout the reaction, indicating that evaporative loss does not meaningfully affect the hole-scavenging capacity of the system.

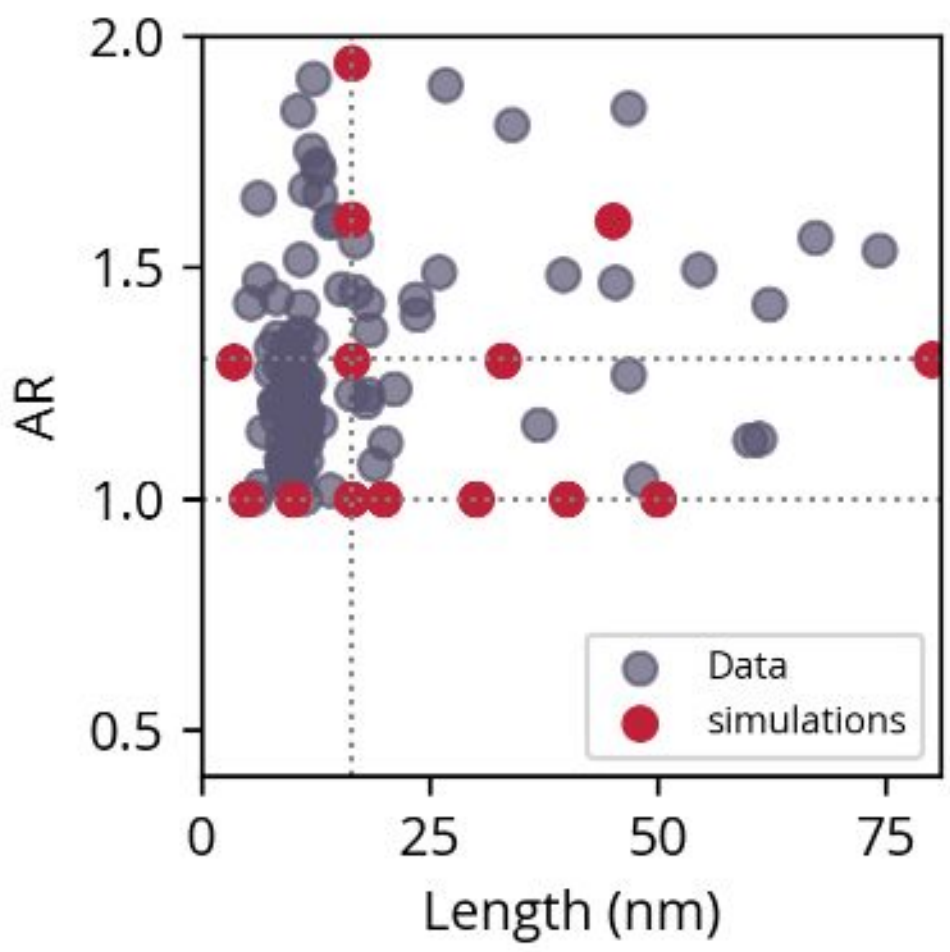

Figure S5: The aspect ratio (AR) as a function of the length of the Au nanoparticles (NPs) for non-hole scavenged Au/ZnO, including the sizes and AR that were chosen for electromagnetic simulations.

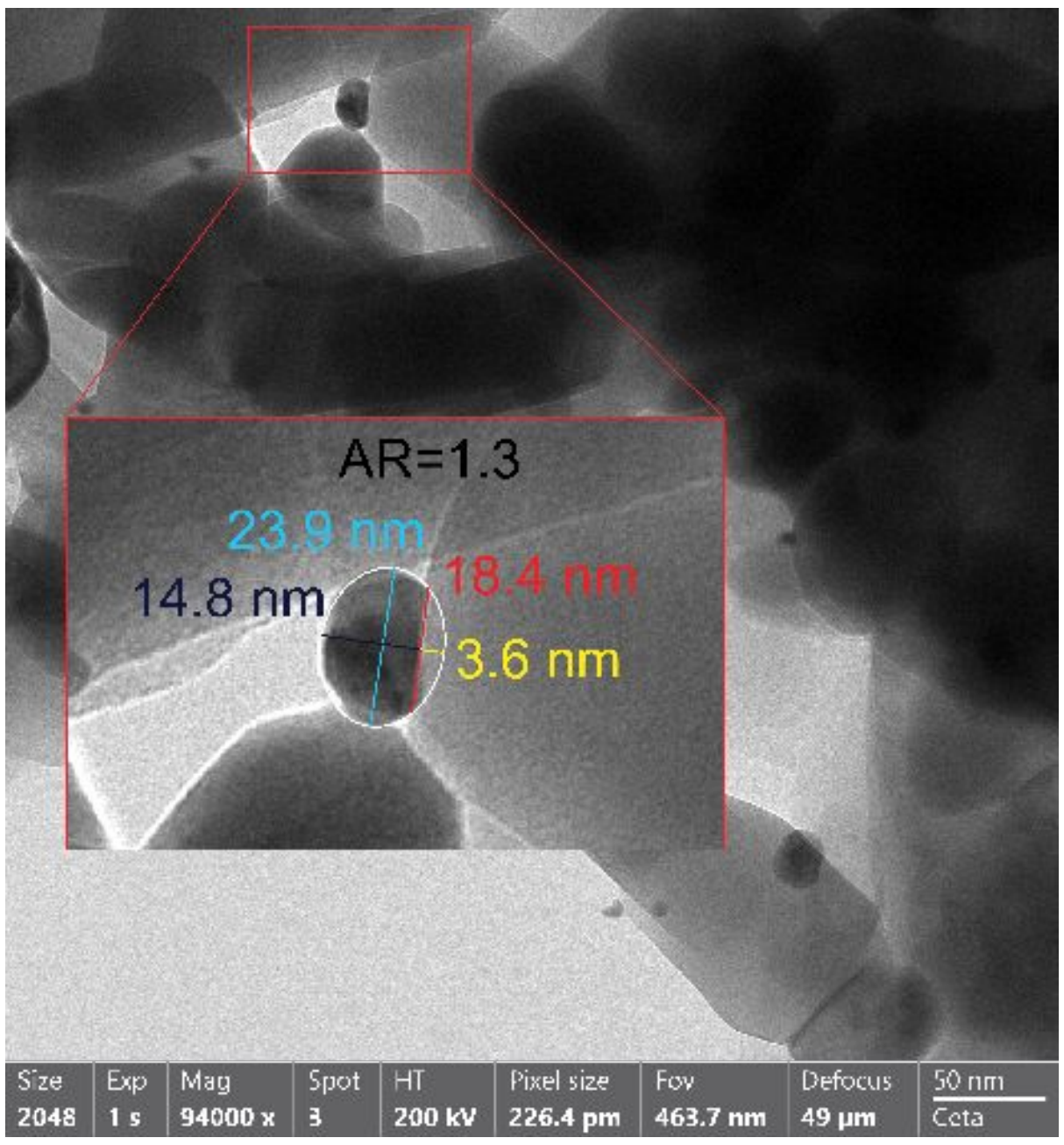

Figure S6: Transmission electron microscopy (TEM) image of Au/ZnO prepared without a hole scavenger. In-set shows the gold truncation of 20 % on to ZnO surface that was used as an estimation for electromagnetic simulations.

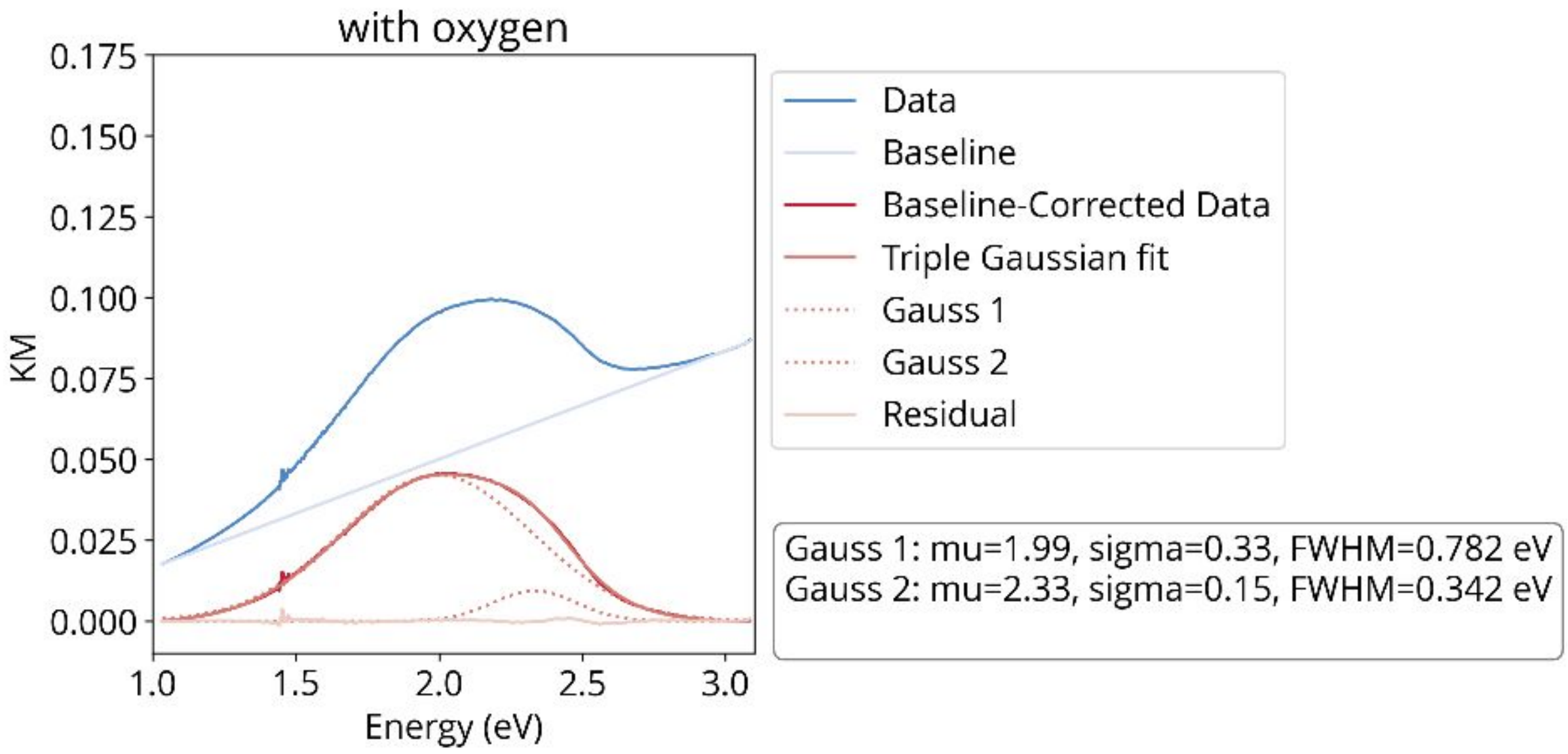

Figure S7: Kubelka-Munk (KM) function zoomed in on Au absorption band fitted with a linear baseline and two Gaussians for a control experiment with ethanol as a hole scavenger and flushing compressed air instead of nitrogen during photodeposition of Au on ZnO.

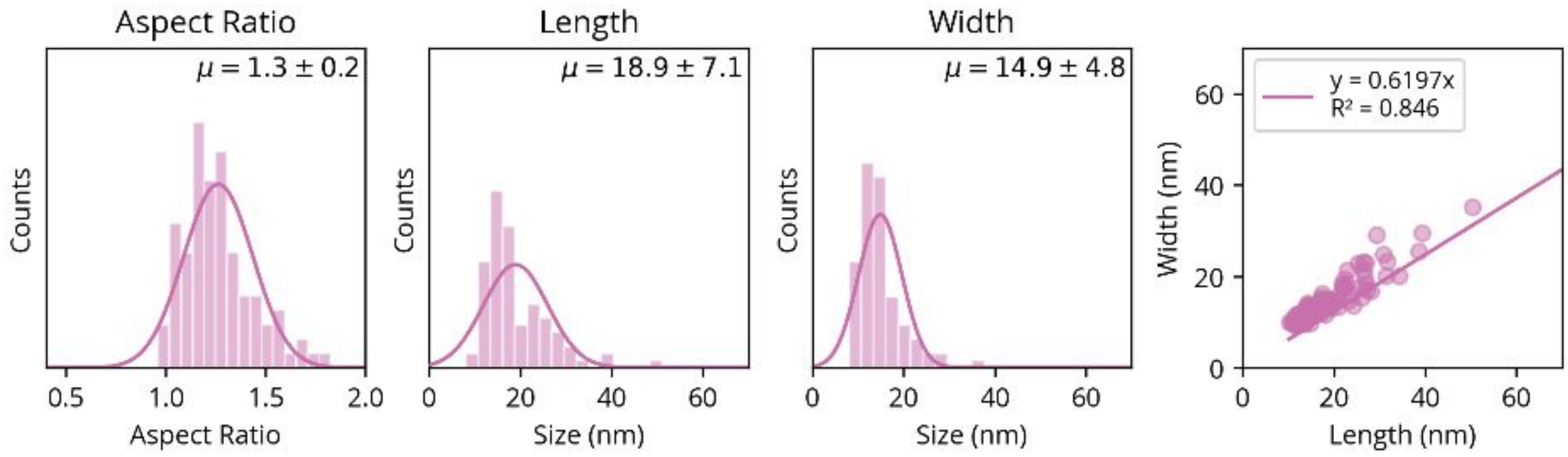

Figure S8: Aspect ratio, length, and width distributions, scattering plot of width as a function of length of Au/ZnO samples prepared with **oxygen** (compressed air) instead of N_2 purging.

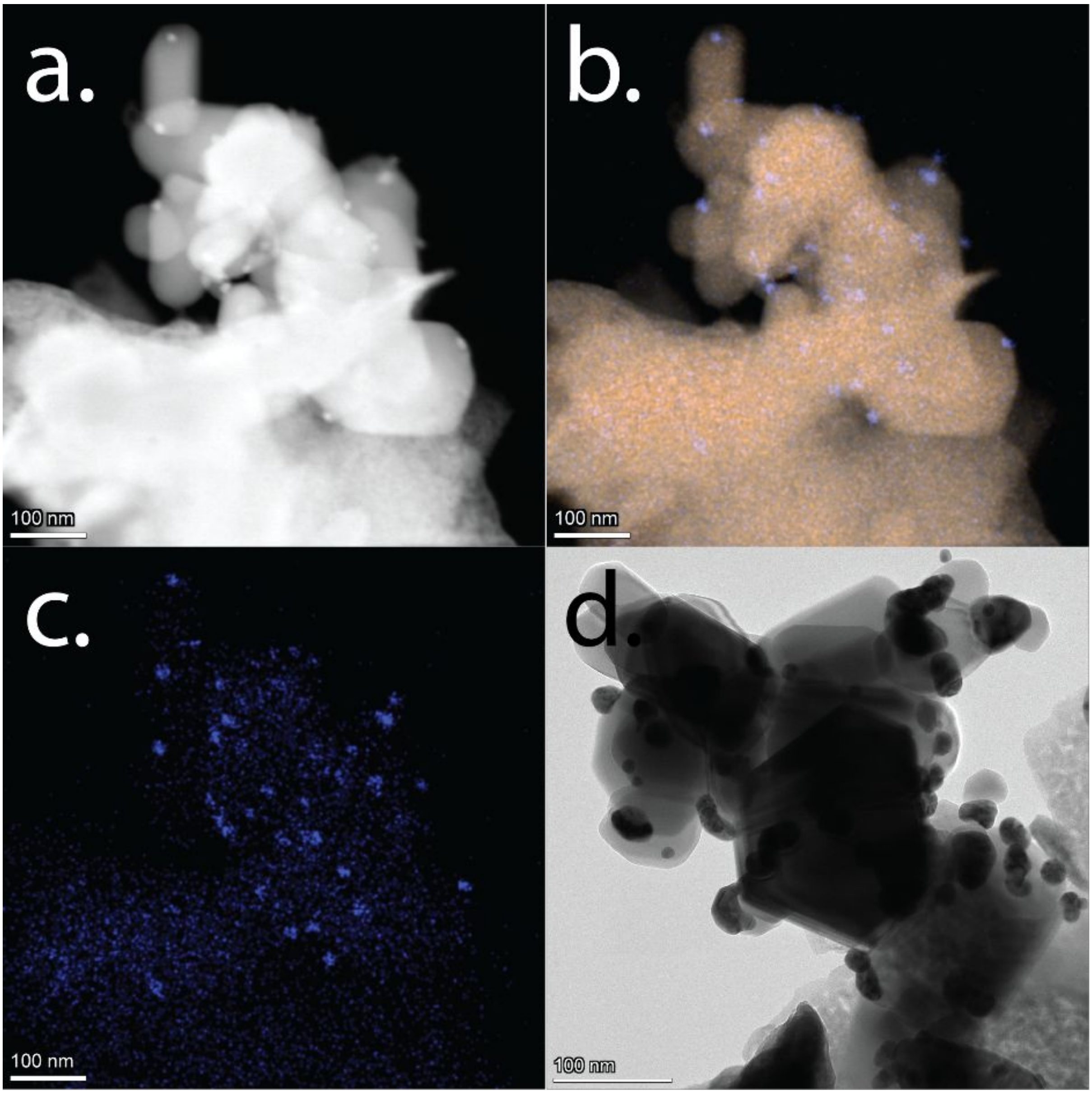

Figure S9: Au/ZnO prepared with oxygen during photodeposition taken with scanning transmission eletron-microscopy – energy dispersive X-ray (STEM-EDX) analysis (a), elemental maps of Zn and Au (b), elemental map of Au (c), and Bright-field transmission electron microscopy (TEM) image (d).

Reactions schemes

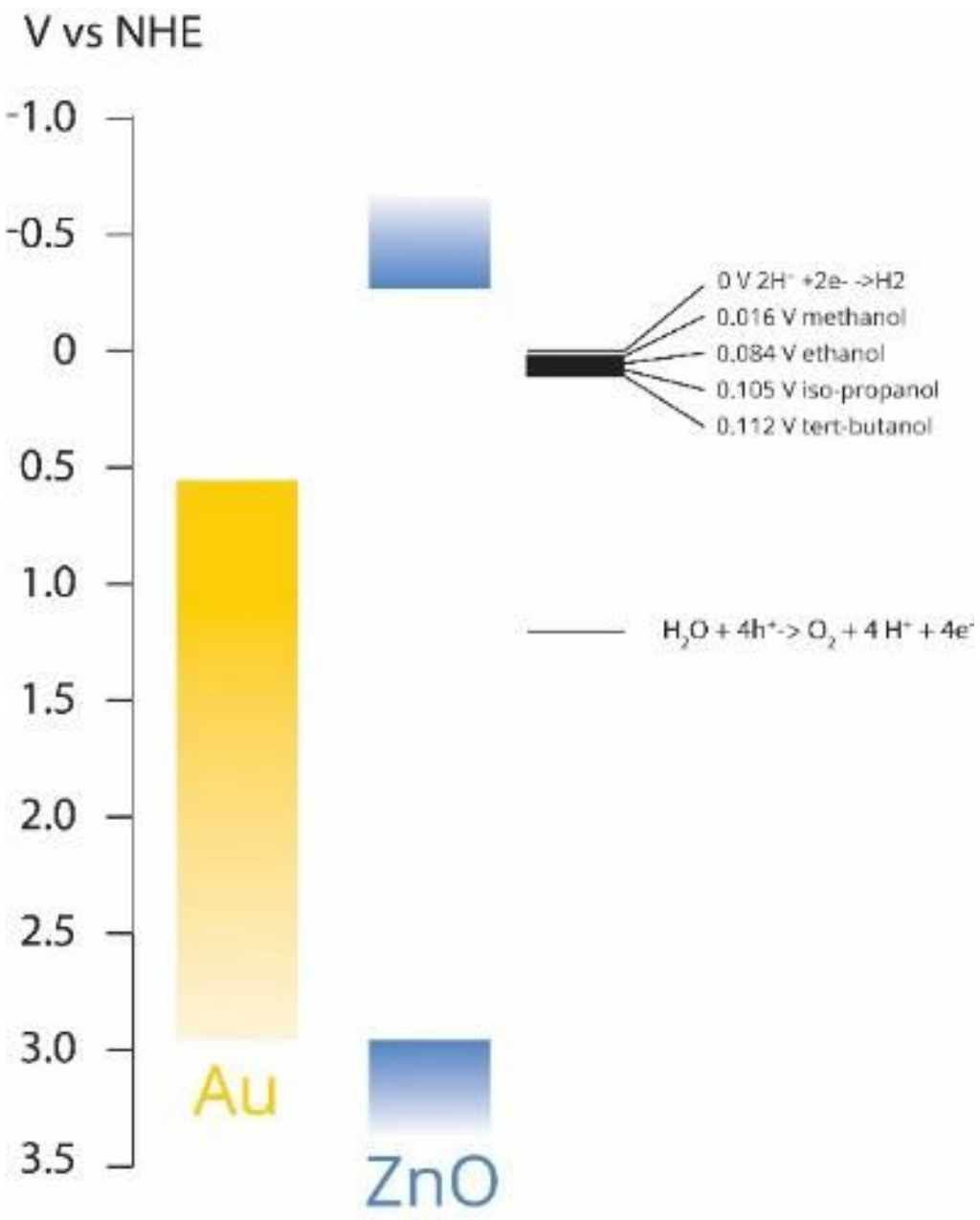

Figure S10: Band positions of Au (Fermi-level of bulk Au is 5.1 eV) [1, 2], ZnO valence and conduction band edges[3] and various oxidation potentials of water oxidation and hole scavenger oxidations.[4]

Upon illumination with enough energy to overcome the ZnO bandgap (3.25 eV), electrons within the ZnO valence band can be photoexcited to the conduction band, creating electron-hole pairs (Eq. S.1).

$ZnO + hv \rightarrow h^+ + e^-$ Eq. S.1

Subsequently, various reduction (Eqs. S.2-4) and oxidation (Eqs. S.5-9) reactions can take place simultaneously. The reduction reactions are 1) the reduction of gold chloride ions in the solution to Au NPs on the ZnO surface (Eq. S.2)[5], 2) hydrogen formation (Eq. S.3)[4], 3) and oxygen reduction (Eq. S.4).[5, 6]

$AuCl_4^- + 3\ e^- \rightarrow Au(0) + 4\ Cl^-$ Eq. S.2

$2\ H^+ + 2\ e^- \rightarrow H_2$ Eq. S.3

$O_2 + 4\ e^- + 4\ H^+ \rightarrow 2\ H_2O$ Eq. S.4

Without a hole scavenger the hole either recombines with the photoexcited electron or oxidizes water (oxygen evolution reaction, OER) in the following reaction[7]:

$2\ H_2O(l) + 4\ h^+ \rightarrow O_2(g) + 4\ H^+\ (aq) + 4e^-$ Eq. S.5

Alcohol oxidation half reactions for the different hole scavengers used in this study[8]

$CH_3OH_{(ads)} + H_2O \rightarrow CO_2 + 6\ H^+ + 6\ e^-$ Eq. S.6

$C_2H_5OH_{(ads)} + 3\ H_2O \rightarrow 2\ CO_2 + 12\ H^+ + 12\ e^-$ Eq. S.7

$C_3H_7OH_{(ads)} + 5\ H_2O \rightarrow 3\ CO_2 + 18\ H^+ + 19\ e^-$ Eq. S.8

$C_4H_9OH_{(ads)} + 7\ H_2O \rightarrow 4\ CO_2 + 24\ H^+ + 24\ e^-$ Eq. S.9

Upon illumination, ZnO can deteriorate via the photo-corrosion mechanism: [9, 10]

$ZnO\ (s) + 2\ h^+ + H_2O \rightarrow Zn^{2+}(aq) + \frac{1}{2}\ O_2 + 2H^+$ Eq. S.10

The gold precursor can also undergo photolysis:[11, 12]

$AuCl_4^- + h\nu \rightarrow AuCl_3^- + Cl\cdot$ Eq. S. 11

$2\ AuCl_3^- \rightarrow AuCl_4^- + AuCl_2^-$ Eq. S. 12

$2\ AuCl_2^- \rightarrow Au(0) + AuCl_4^-$ Eq. S. 13

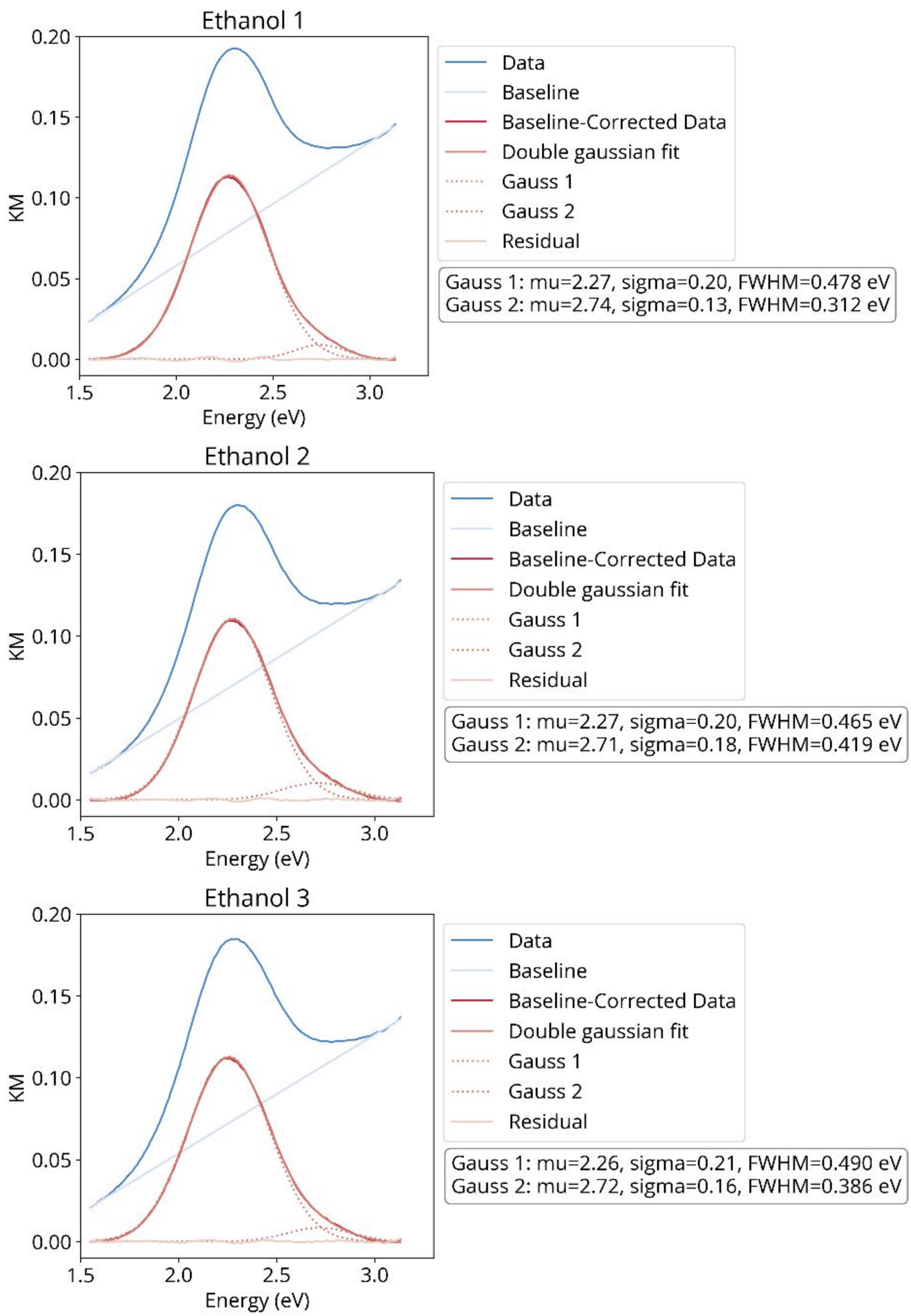

Figure S11: Kubelka-Munk (KM) function zoomed in on Au absorption band fitted with a linear baseline and two Gaussians for each synthesis with ethanol as a hole scavenger during photodeposition of Au on ZnO.

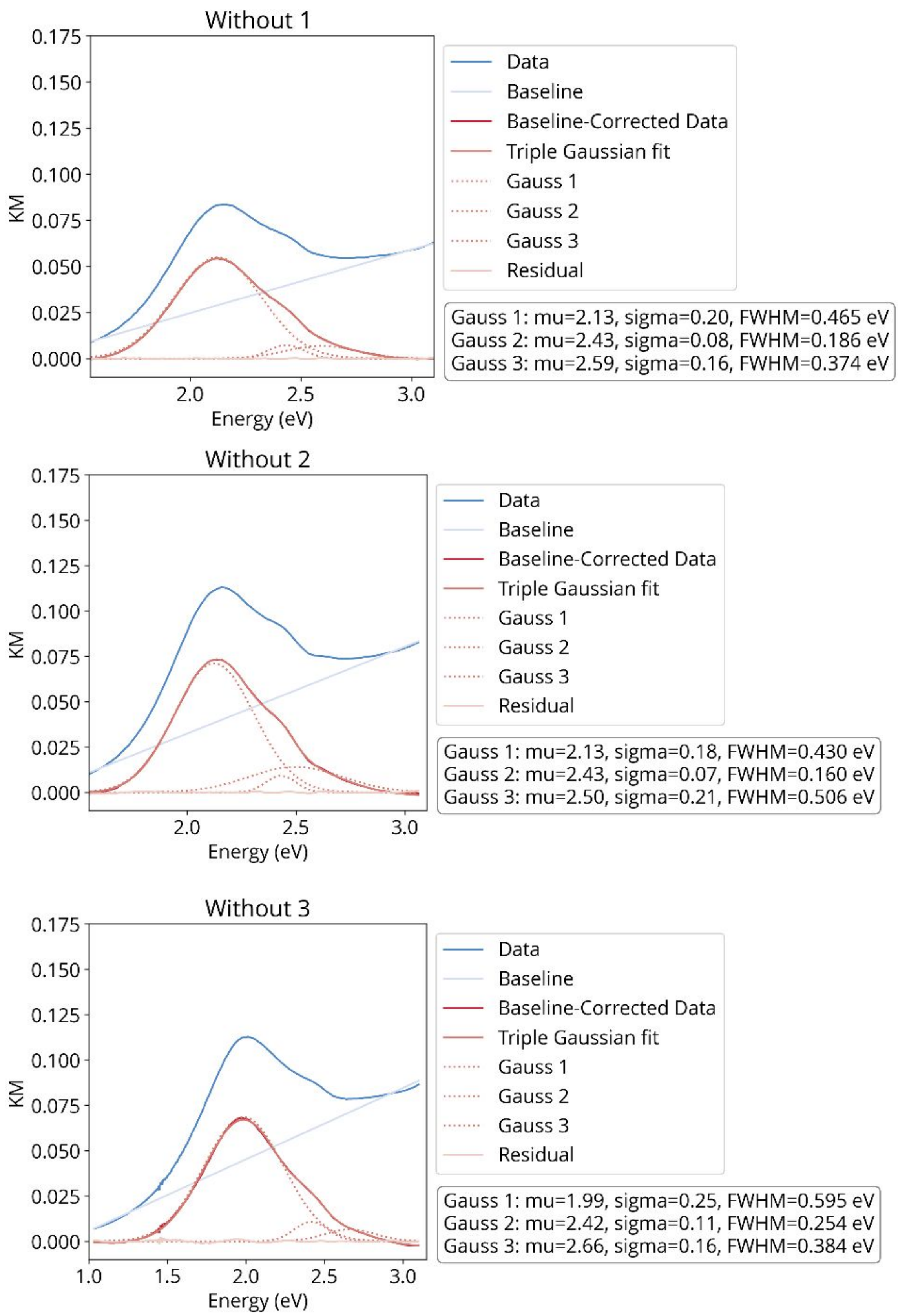

Figure S12: Kubelka-Munk (KM) function zoomed in on Au absorption band fitted with a linear baseline and three Gaussians for each synthesis without a hole scavenger during photodeposition of Au on ZnO (synthesis 1 and 2 were prepared with 0.009 mmol $HAuCl_4$ which yielded 0.6 wt.% Au/ZnO and synthesis 3 was prepared with 0.012 mmol, which yielded 0.9 wt.% Au/ZnO).

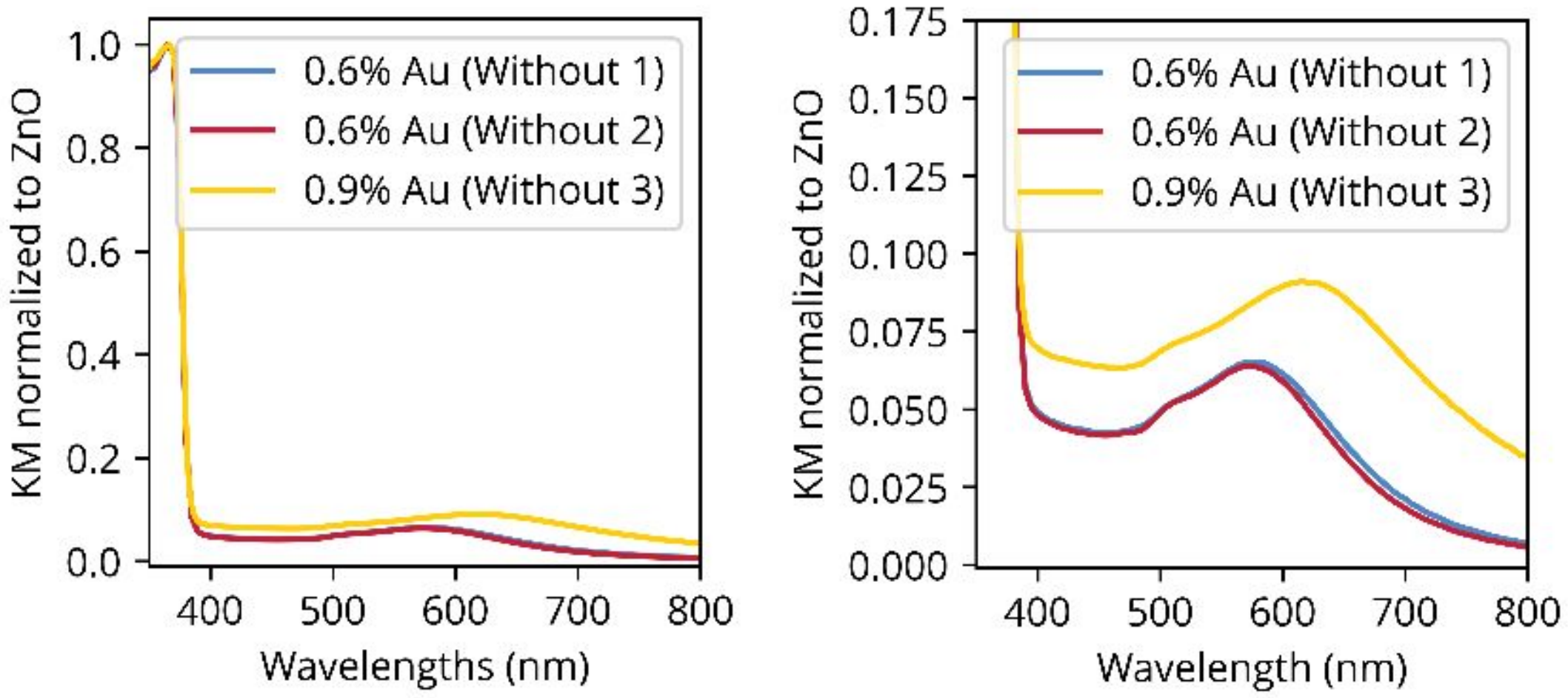

Figure S13: Kubelka-Munk (KM) function of three Au/ZnO photodeposition without the addition of a hole scavenger with different weight loadings of gold (left) and a zoom of the Au band (right). Synthesis 1 and 2 were prepared with 0.009 mmol $HAuCl_4$ which yielded 0.6 wt.% Au/ZnO and synthesis 3 was prepared with 0.012 mmol, which yielded 0.9 wt.% Au/ZnO).

In-situ UV-Vis transmission spectroscopy during equilibrium phase

The process was started by measuring the transmitted photons of a focused white light laser through a continuously stirred ZnO suspension. Then, the Au precursor was added to the suspension, followed by ethanol as a hole scavenger (where applicable). N_2-bubbling was initiated while maintaining continuous stirring and measuring for 30 min to ensure an oxygen-poor environment to avoid competing processes and to obtain an adsorption/desorption equilibrium. After this equilibrium period, the photodeposition is started by UV illumination. This sequence was done for the reaction with and without a hole scavenger, as well as for a ZnO only control.

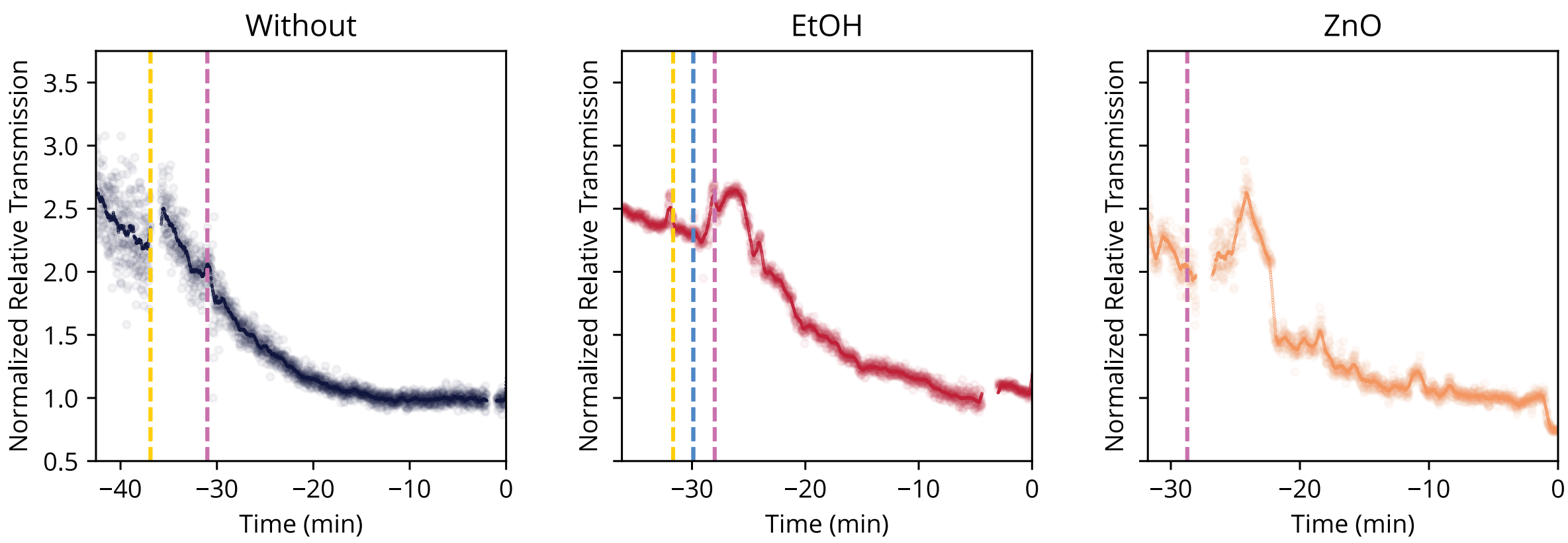

Figure S14: Normalized (to end of equilibrium (t = -0.4 s)) relative transmission at Localized Surface Plasmon Resonance (LSPR) (510 nm for Au/ZnO without ethanol, 547 nm for Au/ZnO with ethanol, and 510 for ZnO suspension) during the sequential stages of the equilibrium period, starting with the ZnO suspension, followed by the addition of Au precursor (yellow dashed line), ethanol (blue dashed line) and the start of N_2 bubbling (pink dashed line).

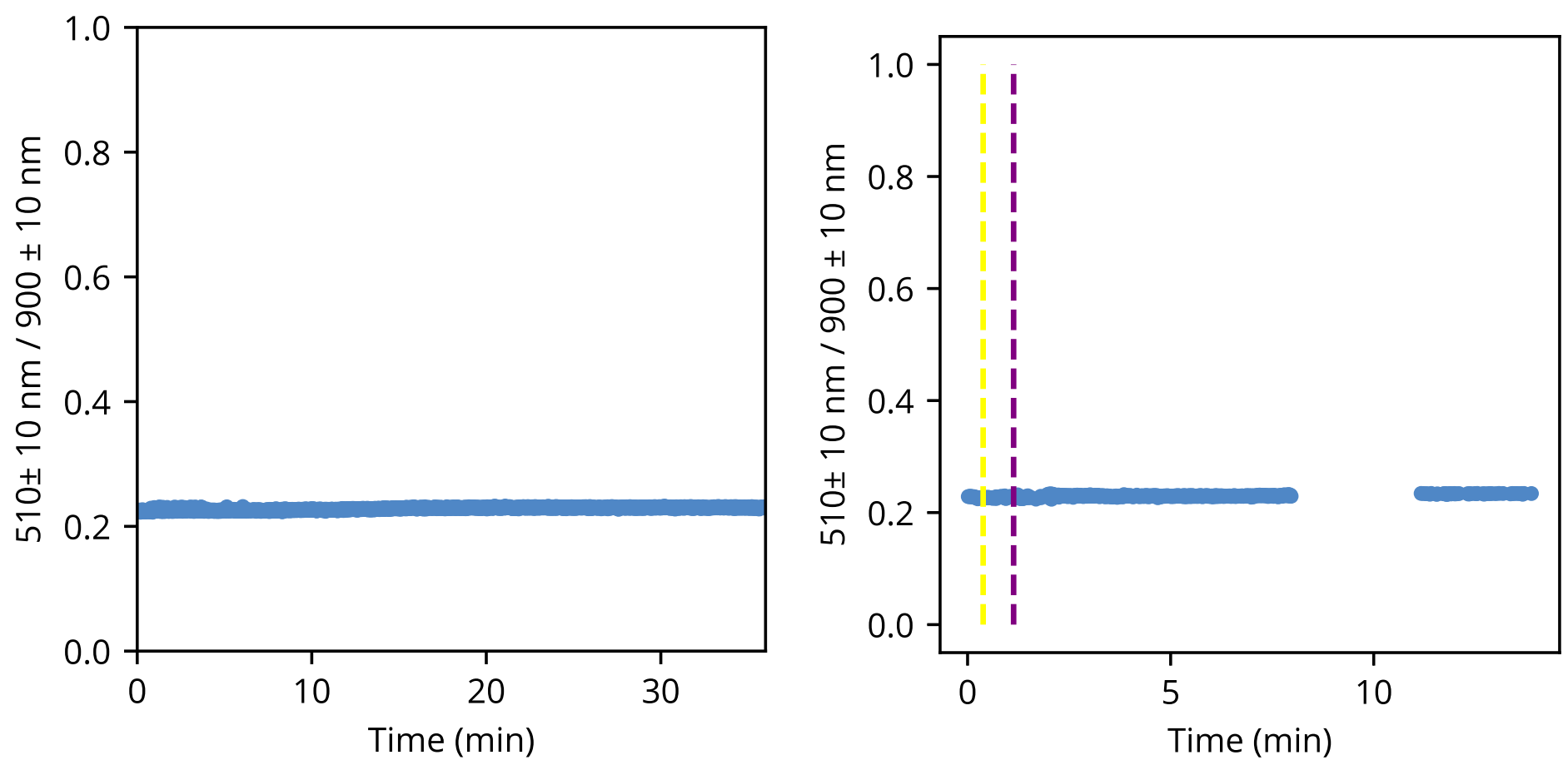

Figure S15: Relative transmission (intensity at 510 ± 10 nm over intensity at 900 ± 10 nm) during *in-situ* UV-Vis spectroscopy measurements of miliQ during UV exposure (left). Continued measurement through addition of Au precursor ($HAuCl_4.3H_2O$) at yellow dashed line, and UV exposure (purple dashed line) (right).

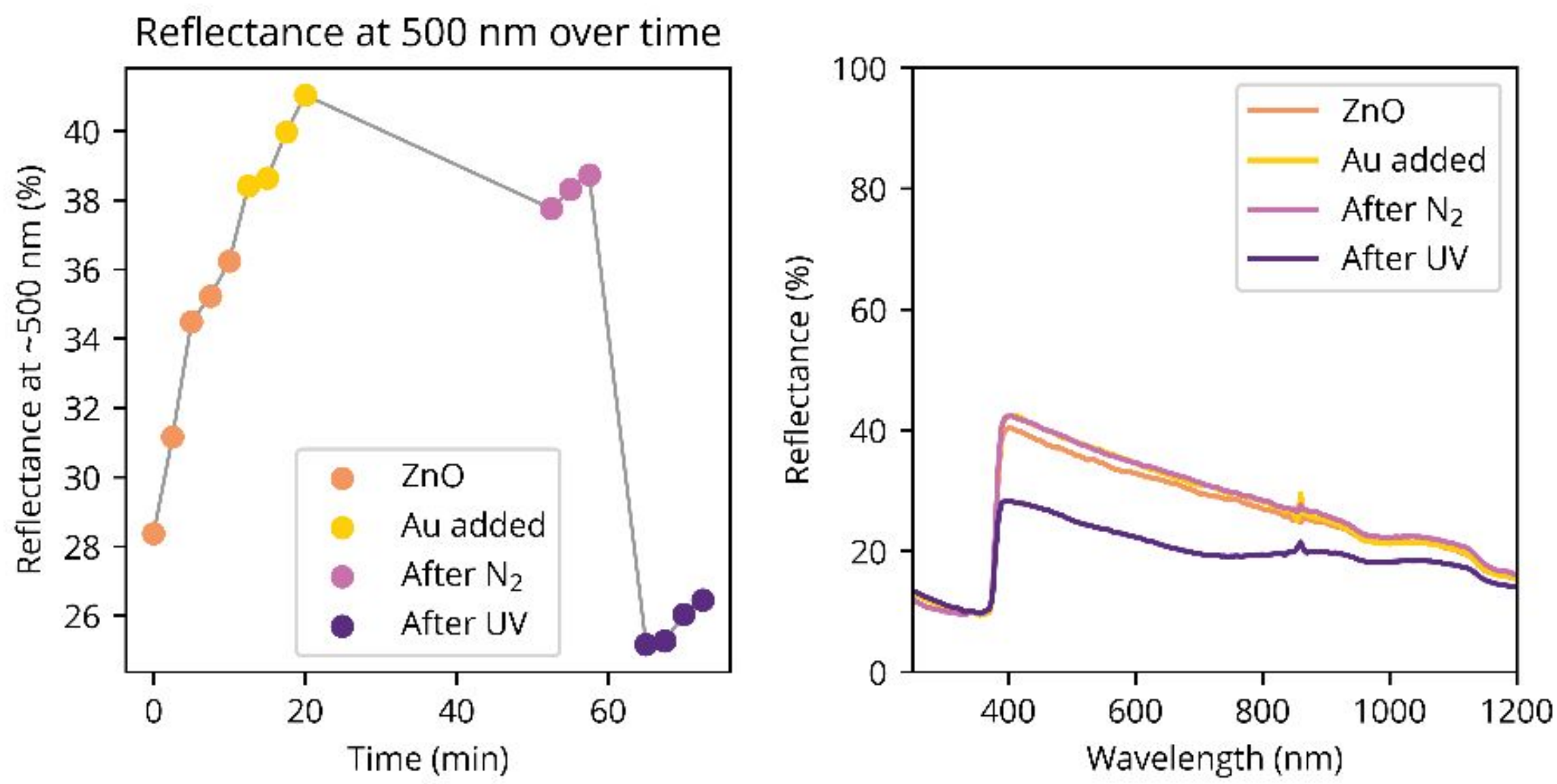

Figure S16: Total reflectance measured *ex-situ* at the exit of the integrating sphere without a back reflector at different steps within the photodeposition of Au on ZnO without a hole scavenger. Stirring was applied during the measurements.

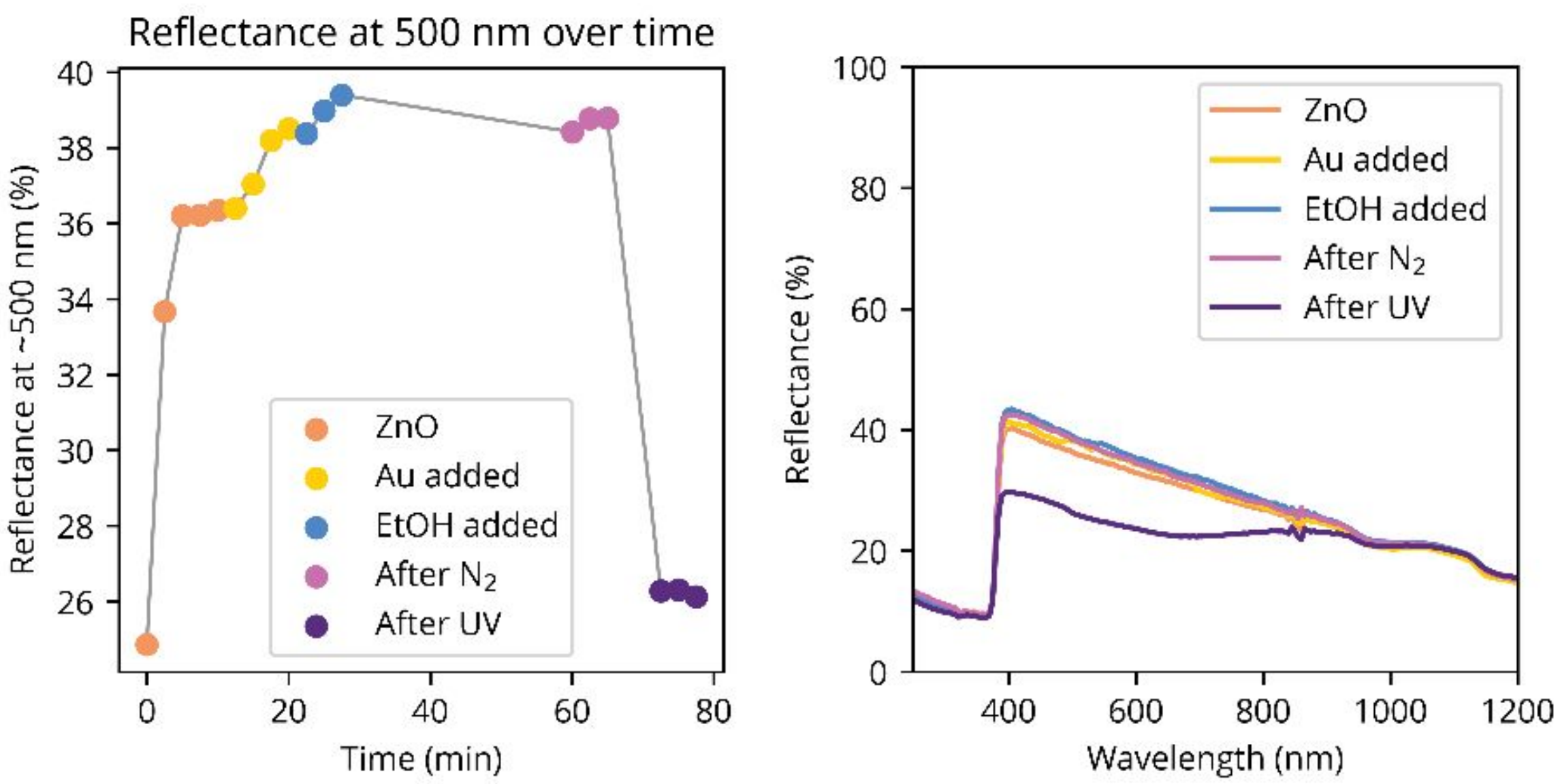

Figure S17: Total reflectance measured ex-situ at the exit of the integrating sphere without a back reflector at different steps within the photodeposition of Au on ZnO with ethanol as a hole scavenger. Stirring was applied during the measurements.

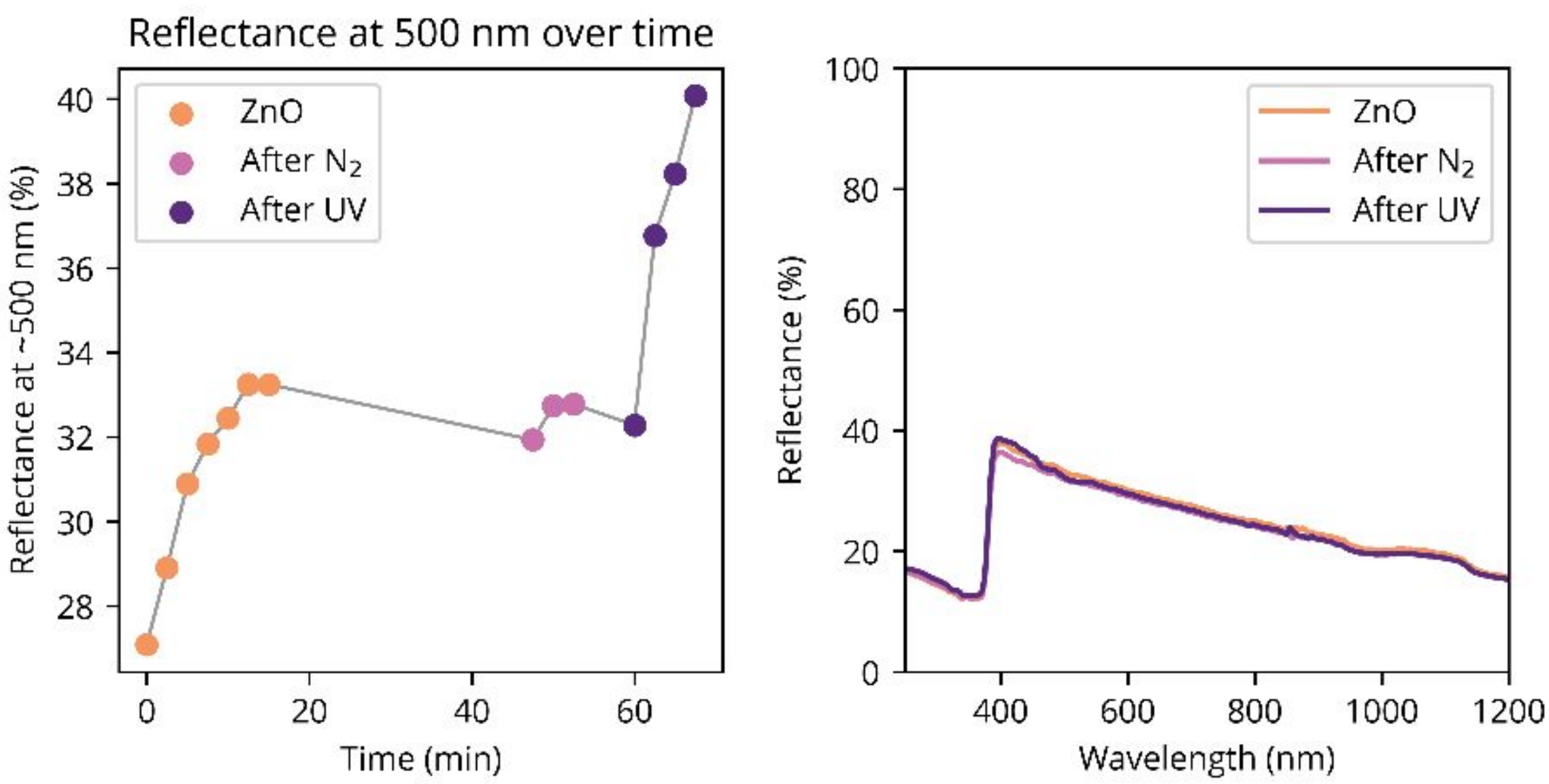

Figure S18: Total reflectance measured *ex-situ* at the exit of the integrating sphere without a back reflector at different steps within the blank reaction with the ZnO suspension. Stirring was applied during the measurements.

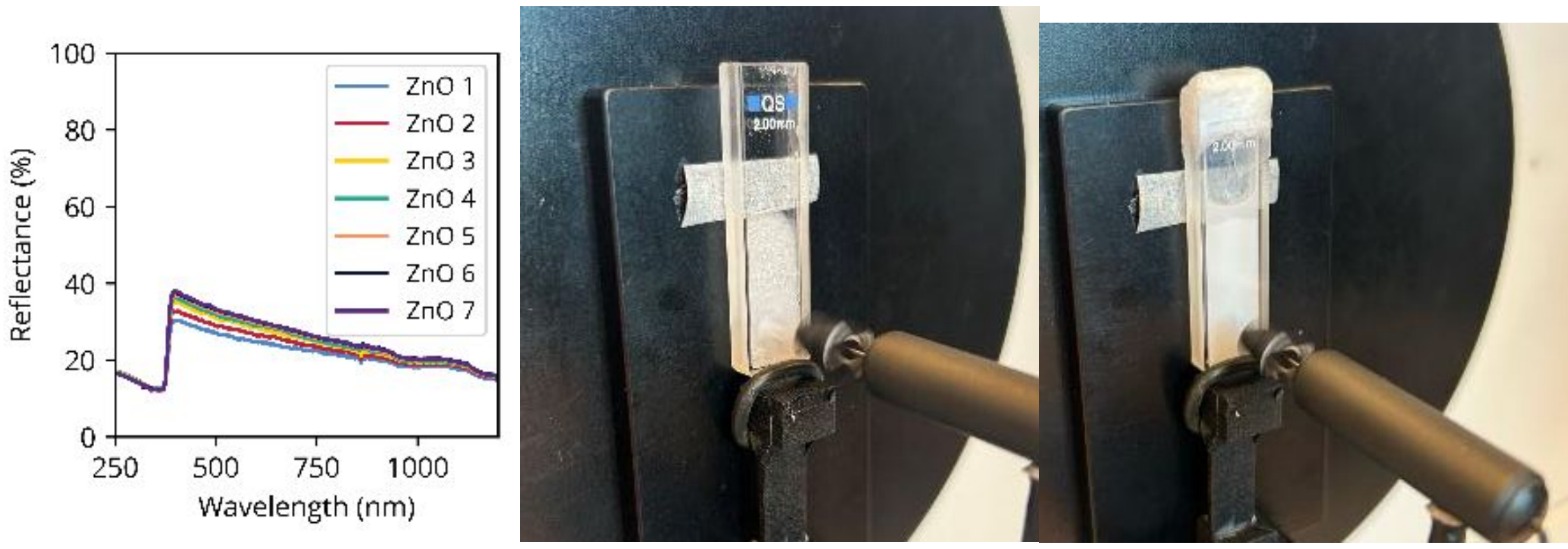

Figure S19: Effect of stirring-induced homogenization on the total reflectance of a ZnO suspension. Seven consecutive total reflectance spectra of a ZnO suspension, recorded upon the onset of stirring. Photographs of the cuvette taken (left) within the first few seconds of stirring, showing larger, unevenly dispersed ZnO clumps/aggregates, and (right) after the equilibrium period, showing a visibly more opaque and uniformly dispersed suspension.

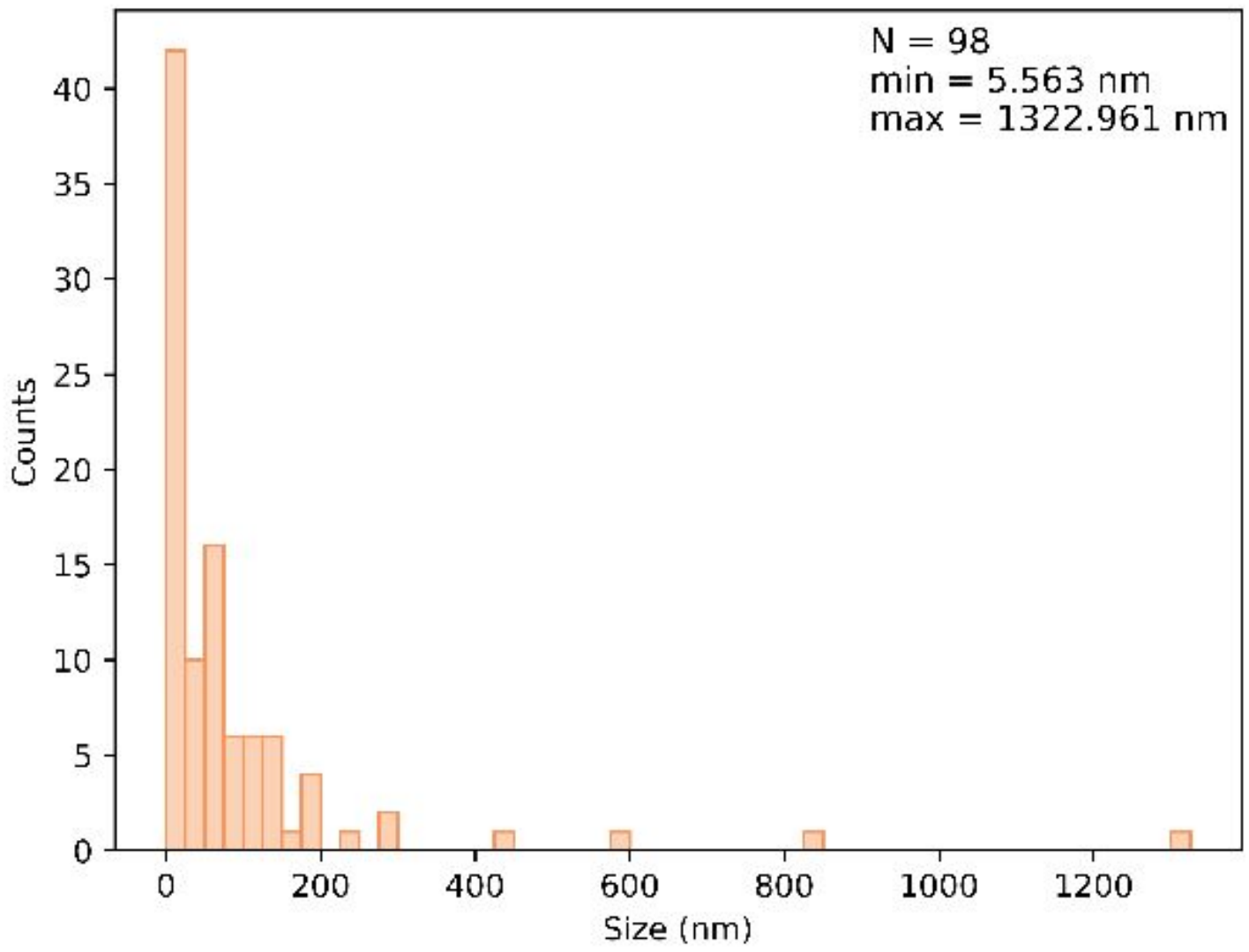

Figure S20: Particle size distribution of ZnO particles.

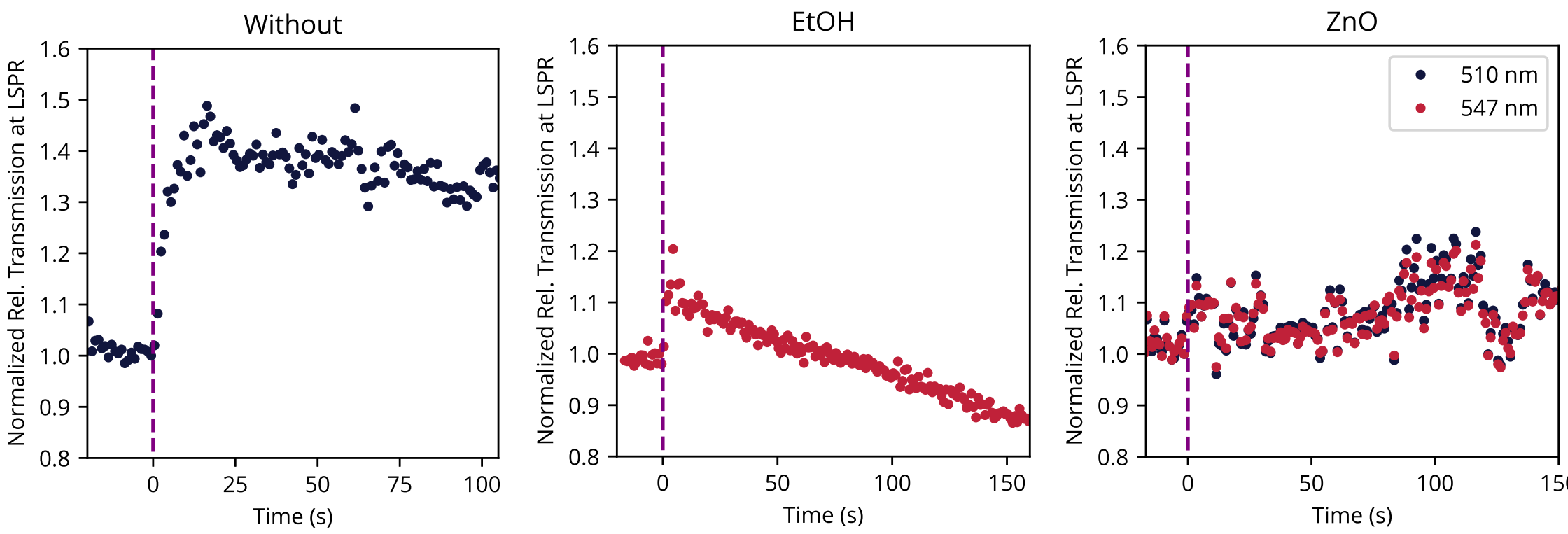

Figure S21: Normalized relative transmission at Localized Surface Plasmon Resonance (LSPR) during photo-deposition of Au on ZnO with and without ethanol as hole scavenger or the illumination of a ZnO suspension. In this experiment He was used instead of N_2 to remove O_2 from the suspension. This may not be the best gas to remove O_2 as it is very light, therefore later experiments were done with N_2 to imitate *ex-situ* syntheses.

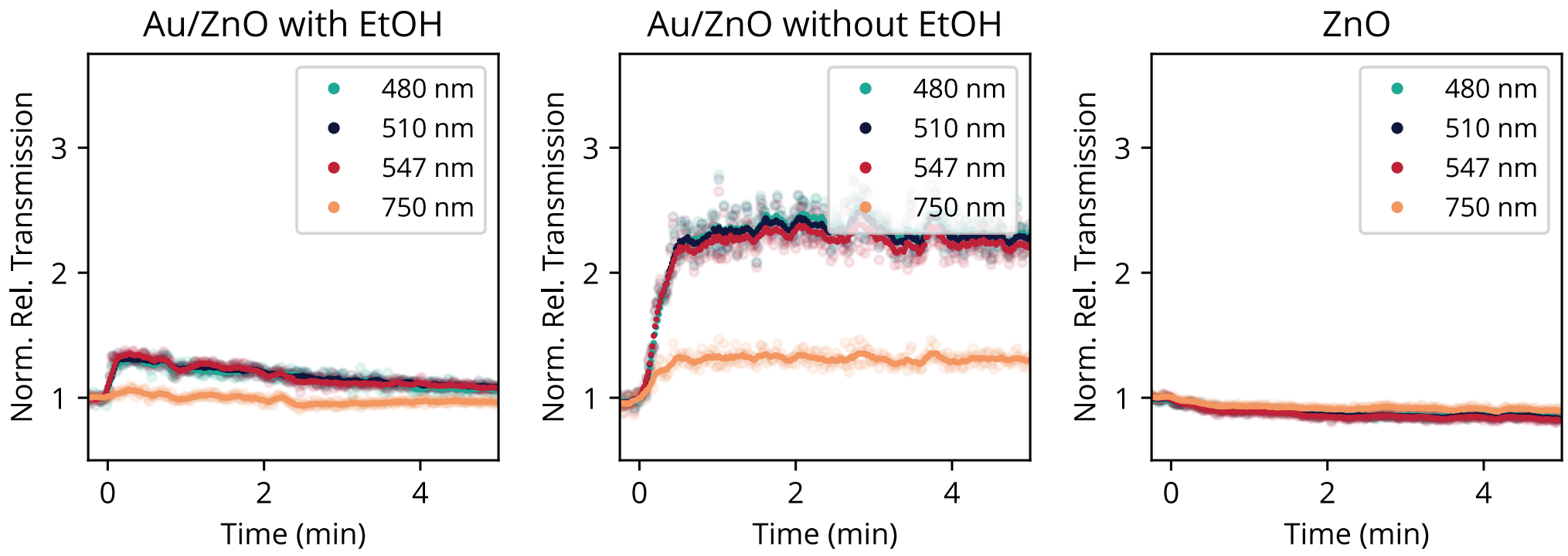

Figure S22: Normalized relative transmission at different wavelengths during photodeposition of Au on ZnO with (left) and without ethanol (middle) as hole scavenger, and illumination of a ZnO suspension (right). Lighter data points are all data points, whereas darker data points are a running average with a Δt = 10 s.

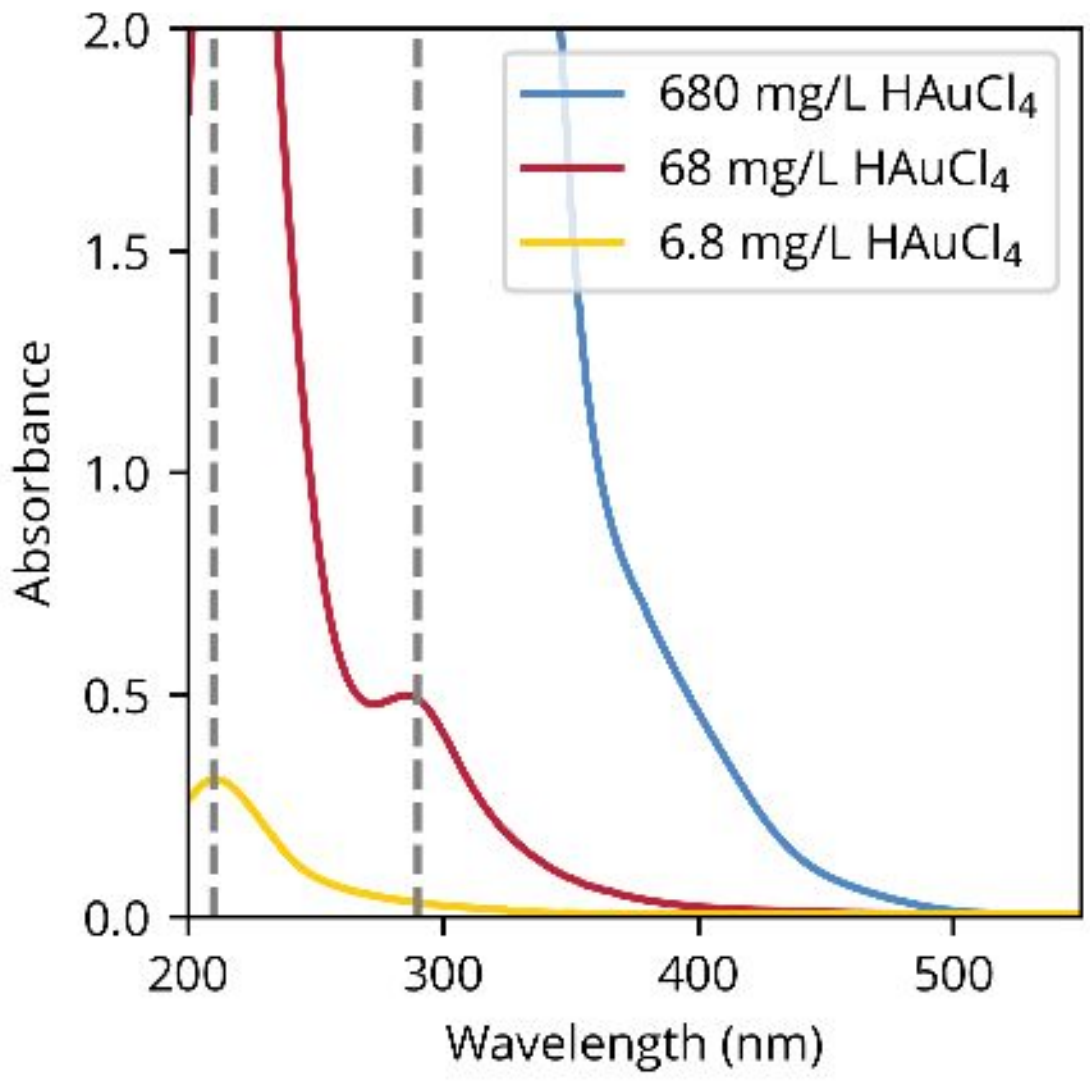

Figure S23: Absorbance of Au precursor solution measured in 10 mm pathlength cuvette in transmission mode.

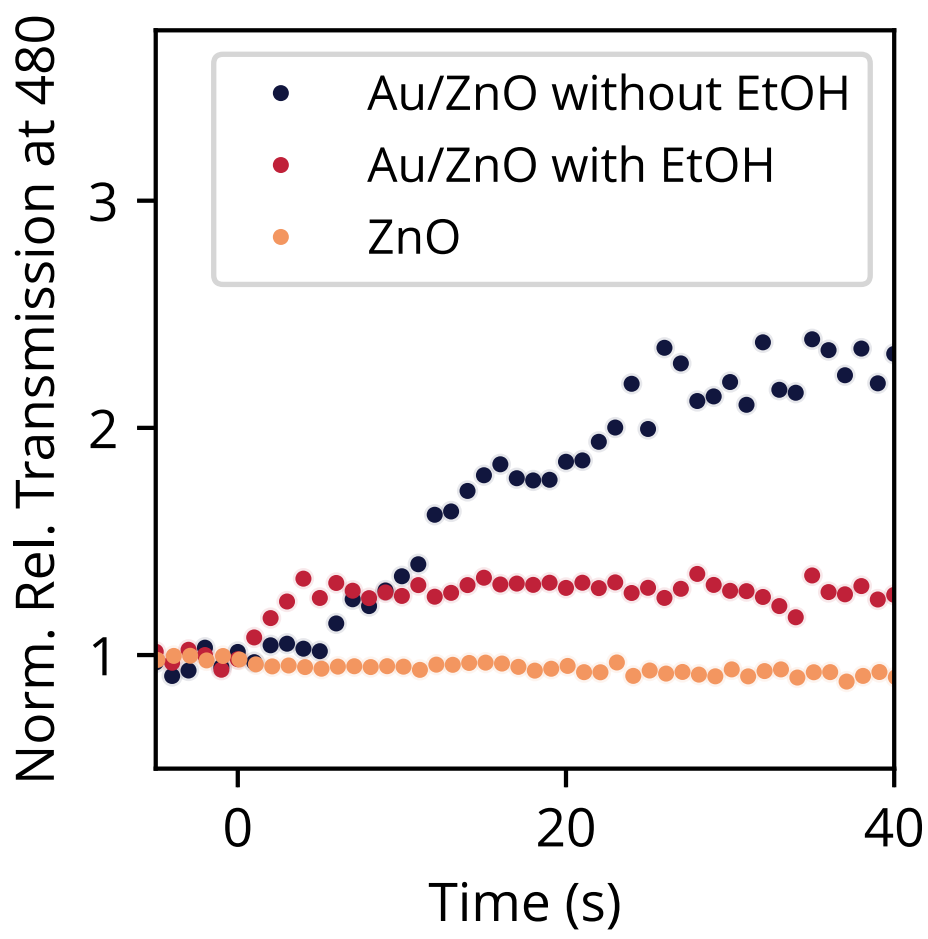

Figure S24: Normalized relative transmission at 480 nm during photodeposition of Au on ZnO with and without ethanol as hole scavenger, and illumination of a ZnO suspension.

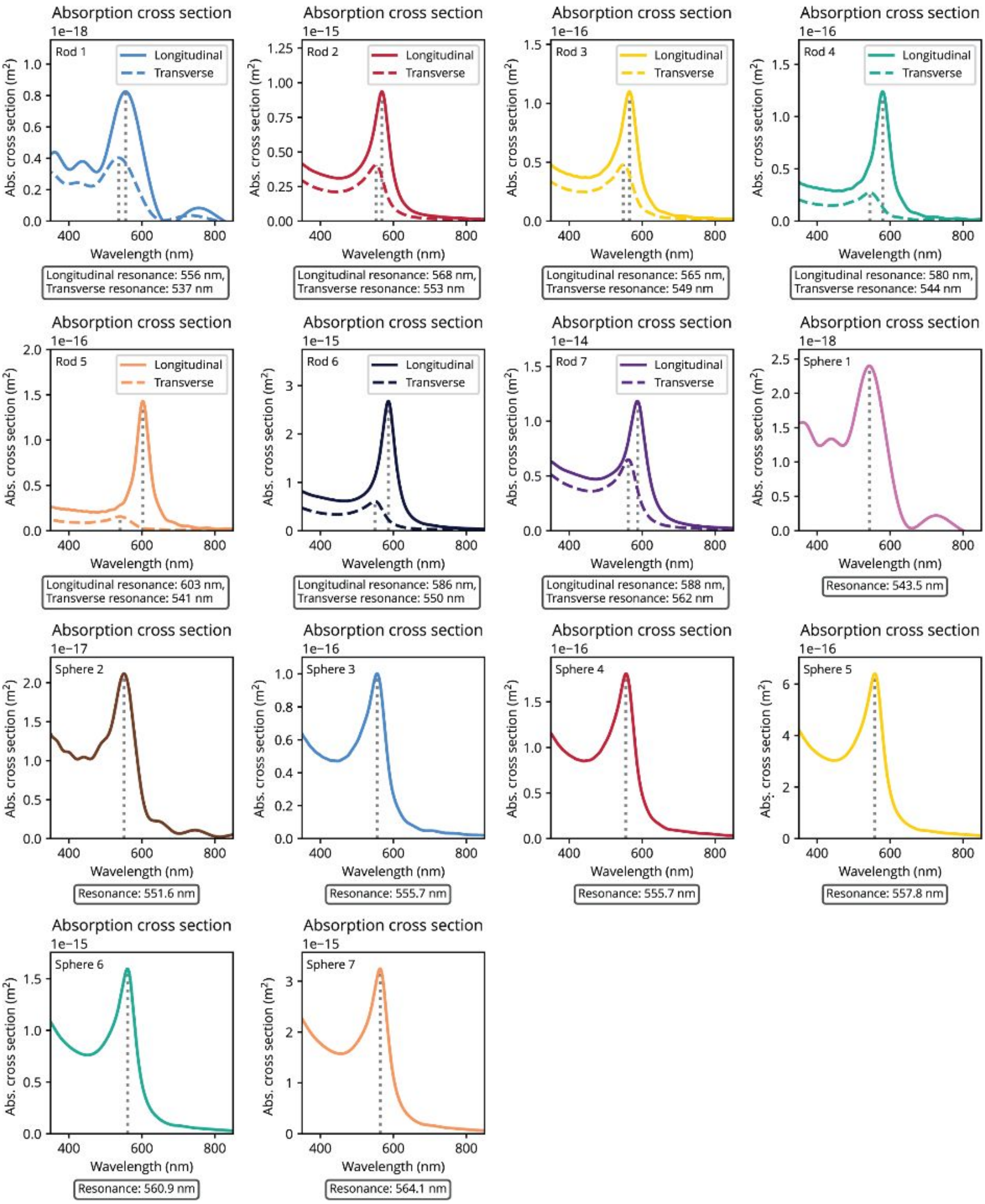

Figure S25: Electromagnetic simulations with different polarization to excite the longitunidal and transverse mode for each Au nanoparticle (NP) (length, width, aspect ratio (AR) and the modeling input radii of the simulated Au NPs can be found in Table 1 in the main text).

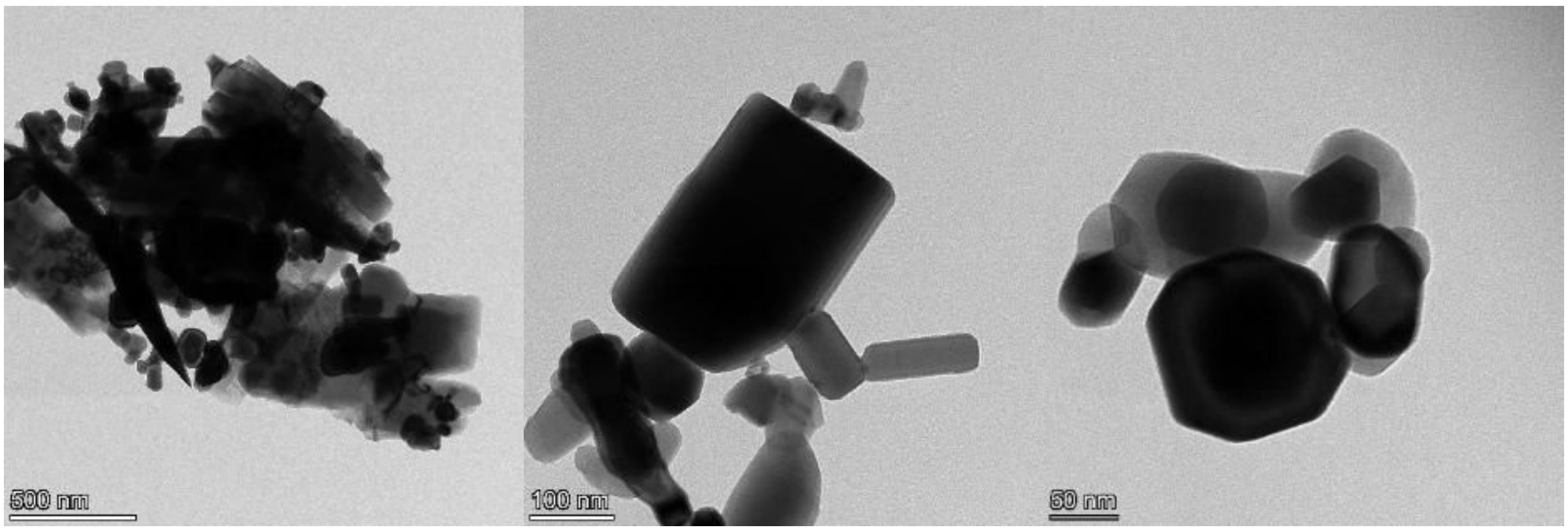

Figure S26: Bright-field transmission electron Microscopy (TEM) images of bare ZnO.

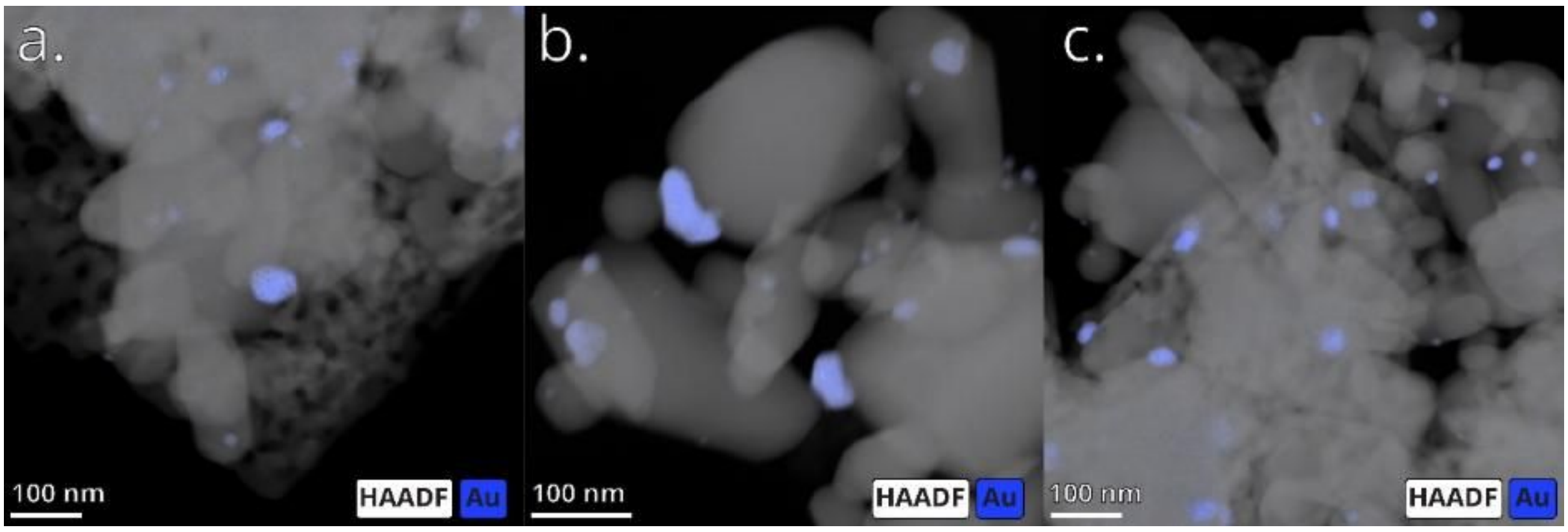

Figure S27: Dark-field scanning transmission electron microscopy–energy dispersive X-ray (STEM-EDX) analysis images of Au/ZnO prepared without a hole scavenger **0.6 wt.% Au**; gold elemental map displayed in blue.

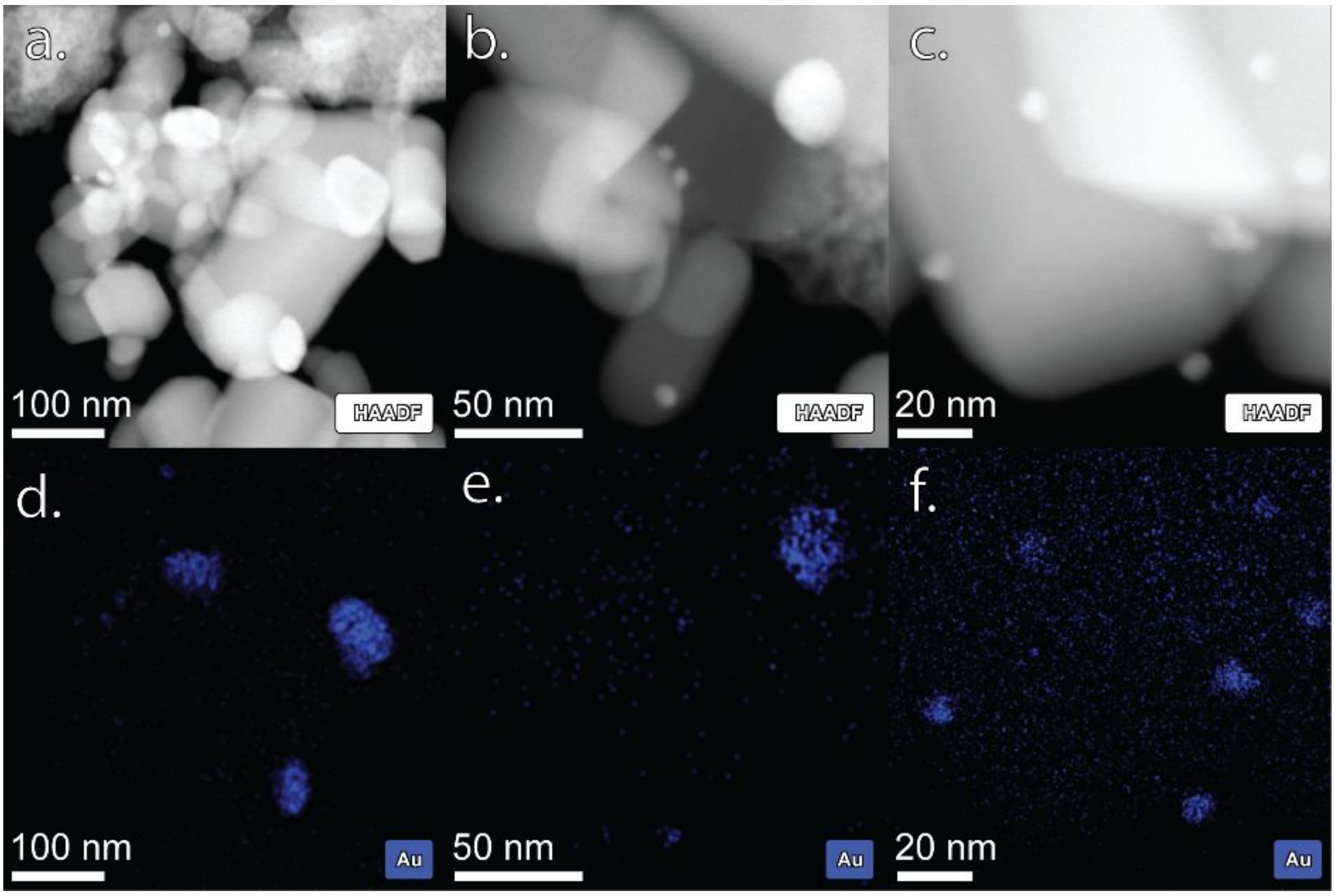

Figure S28: Dark-field scanning transmission electron microscopy–energy dispersive X-ray (STEM-EDX) analysis images of Au/ZnO prepared without a hole scavenger **0.9 wt.% Au**; gold elemental map displayed in blue.

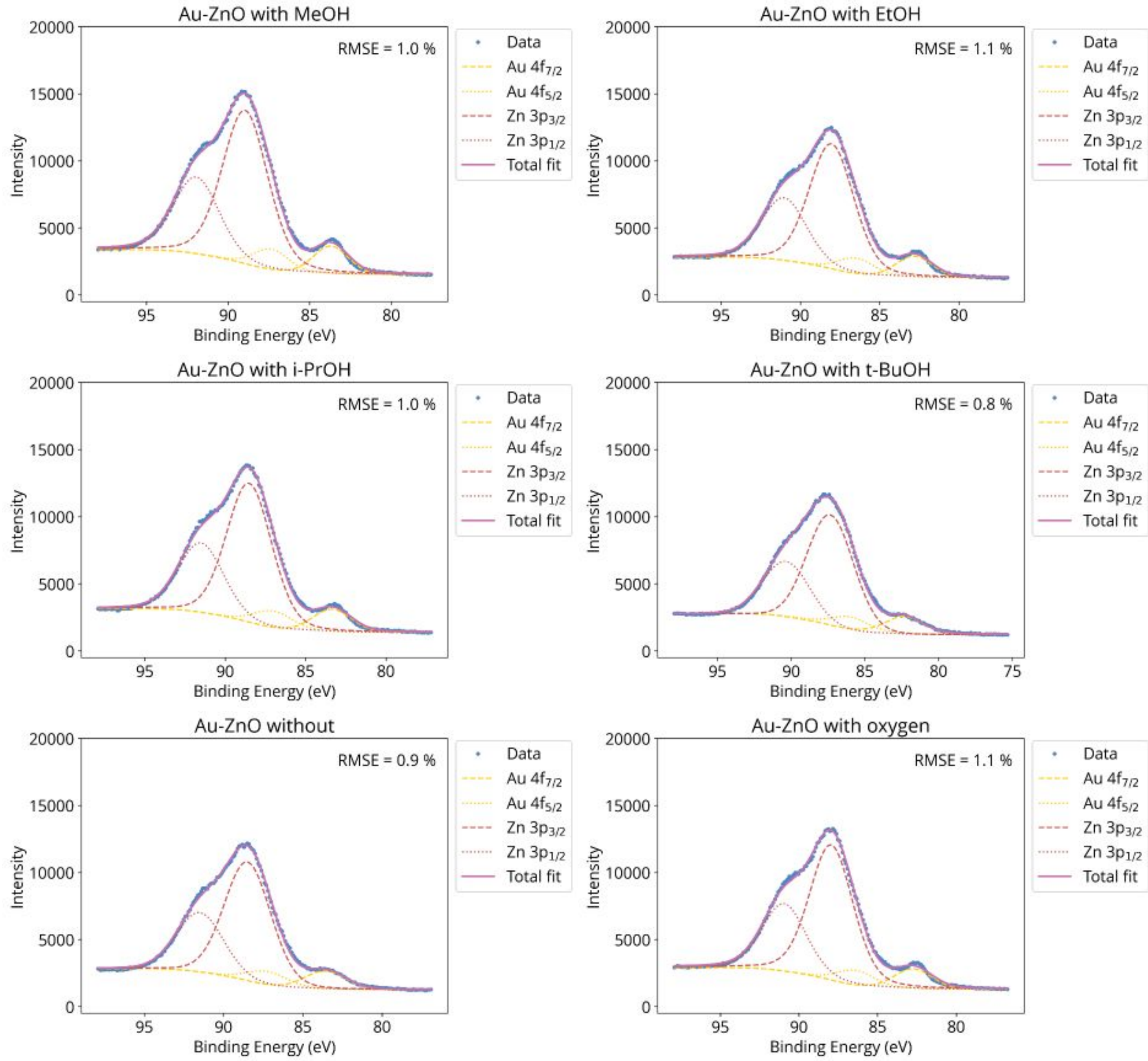

Figure S29: X-ray photoelectron spectroscopy (XPS) data of the Au 4f binding energy region for Au/ZnO samples prepared with different hole scavengers added, without a hole scavenger present, and with oxygen. The Au $4f_{7/2}$, Au $4f_{5/2}$, Zn $3p_{3/2}$, and Zn $3p_{1/2}$ were fitted with four pseudo-Voigt profiles, using fixed spin-orbit splitting, fixed Full-Width at Half Maximum (FWHM) and fixed area ratios. The Root Mean Square Error (RMSE) of the total fit are displayed for each preparation condition.

Table S1: Binding energies (BE) of the fitted Au 4f and Zn 3p components (as shown in Figure S29).

Preparation condition	***Au 4f$_{7/2}$ (eV)***	***Au 4f$_{5/2}$ (eV)***	***Zn 3p$_{3/2}$ (eV)***	***Zn 3p$_{1/2}$ (eV)***
Methanol	83.7	87.4	89.0	92.0
Ethanol	82.8	86.5	88.0	91.0
Iso-propanol	83.4	87.0	88.5	91.5
Tert-butanol	82.4	86.0	87.3	90.3
Without a hole scavenger (0.9 wt.% Au aim)	83.6	87.3	88.5	91.5
With oxygen	82.7	86.4	87.9	90.9

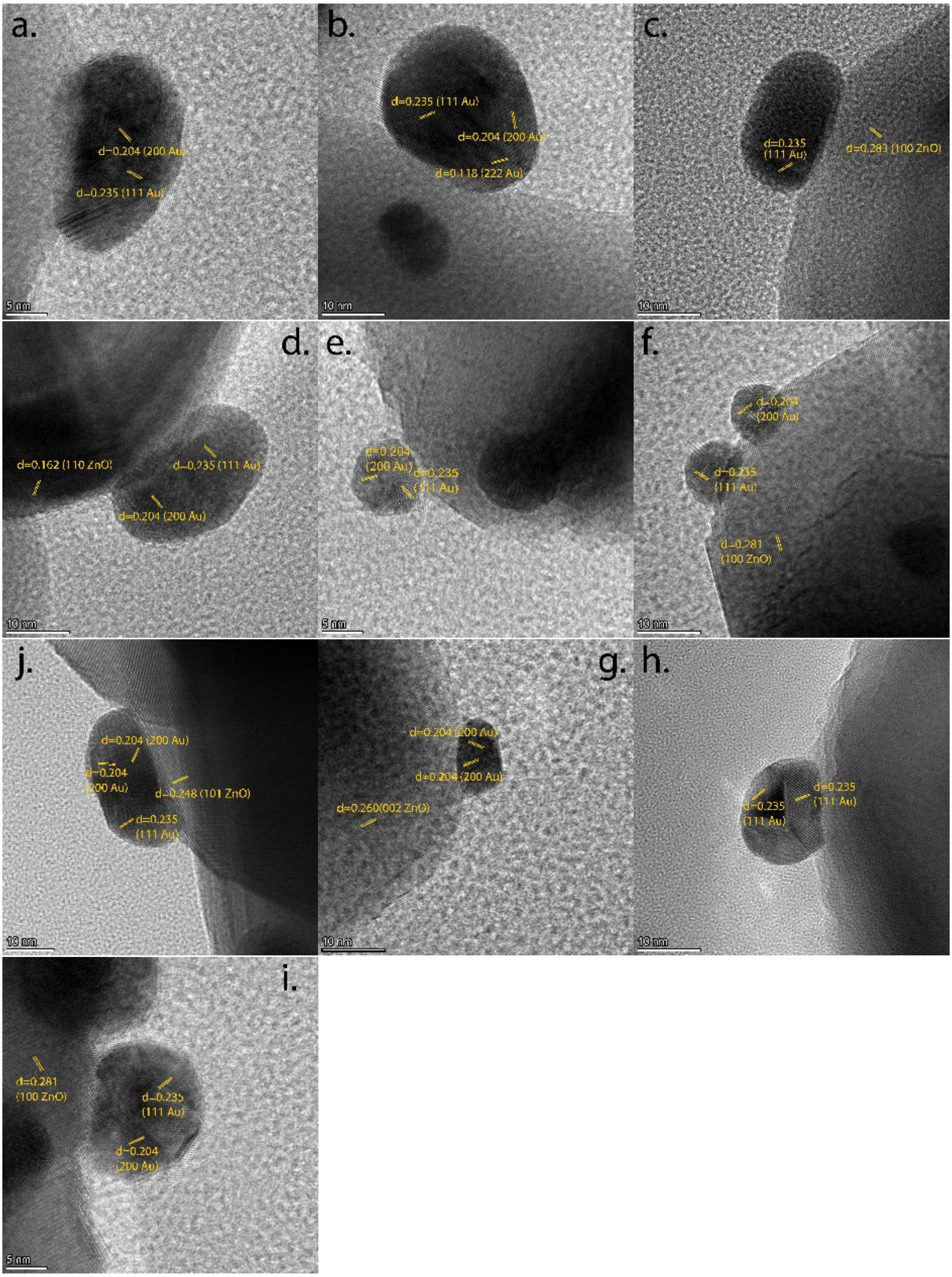

Figure S30: High resolution-transmission electron microscopy (HR-TEM)/fast Fourier transform (FFT)-derived lattice d-spacings for Au/ZnO samples prepared with different conditions. Prepared with methanol (a, b), ethanol (c), *iso*-propanol (d), *tert*-butanol (e, f) as a hole scavenger, without a hole scavenger (g, h), and with oxygen bubbling during photodeposition (i, j). An overview of the d-spacings can be found in Table S2.

Table S2: High resolution-transmission electron microscopy (HR-TEM)/fast Fourier transform (FFT)-derived lattice d-spacings for Au/ZnO samples with different hole scavengers added, without a hole scavenger present, and with oxygen. Compared to reference values from PDF5 cards for Au (00-004-0784), A_2O_3 (00-043-1039), and ZnO (Wurtzite, 00-036-1451).

Sample	*Letter in Figure S30*	*Measured d (nm)*	*Assigned phase*	*Assigned plane*	*Reference d (nm)*	*Δ (%)*
Au/ZnO MeOH	a	0.233	Au	111	0.235	0.9
		0.227	Au	111	0.235	3.4
		0.208	Au	200	0.204	2.0
		0.147	Au	220	0.144	2.1
Au/ZnO MeOH	b	0.238	Au	111	0.235	1.3
		0.222	Au	111	0.235	5.5
		0.204	Au	200	0.204	0.0
		0.118	Au	222	0.118	0.0
		0.161	ZnO	110	0.162	0.6
Au/ZnO EtOH	c	0.286	ZnO	100	0.281	1.8
		0.233	Au	111	0.235	0.9
		0.189	ZnO	102	0.191	1.0
		0.143	Au	220	0.144	0.7
		0.118	Au	222	0.118	0.0
Au/ZnO i-PrOH	d	0.278	ZnO	100	0.281	1.1
		0.238	Au	111	0.235	1.3
		0.204	Au	200	0.204	0.0
		0.164	ZnO	110	0.163	0.6
Au/ZnO t-BuOH	e	0.232	Au	111	0.235	1.3
		0.202	Au	200	0.204	1.0
Au/ZnO t-BuOH	f	0.278	ZnO	100	0.281	1.1
		0.238	Au	111	0.235	1.3
		0.204	Au	200	0.204	0.0
Au/ZnO without	g	0.258	ZnO	002	0.261	1.3
		0.209	Au	200	0.204	2.7
		0.201	Au	200	0.204	1.5
		0.130	ZnO	004	0.131	0.6
Au/ZnO without	h	0.147	Au	220	0.144	2.0
		0.117	Au	222	0.118	0.9
		0.234	Au	111	0.235	0.6
Au/ZnO with oxygen	i	0.286	ZnO	100	0.281	1.7
		0.230	Au	111	0.235	2.2
		0.206	Au	200	0.204	0.9
Au/ZnO with oxygen	j	0.248	ZnO	101	0.248	0.1
		0.205	Au	200	0.204	0.5
		0.234	Au	111	0.235	0.3
		0.191	ZnO	102	0.191	0.1

Table S3: Gold weight loading photodeposited on ZnO with different hole scavengers added, without a hole scavenger present, and with oxygen.

Preparation conditions	*Aimed weight percentage (wt. %)*	*Au loading (wt. %)*
Methanol	0.9	0.93
Ethanol	0.9	0.92
Iso-propanol	0.9	0.90
Tert-butanol	0.9	0.94
Without a hole scavenger	0.9	0.91
Without a hole scavenger	0.7	0.61
With oxygen	0.9	0.93

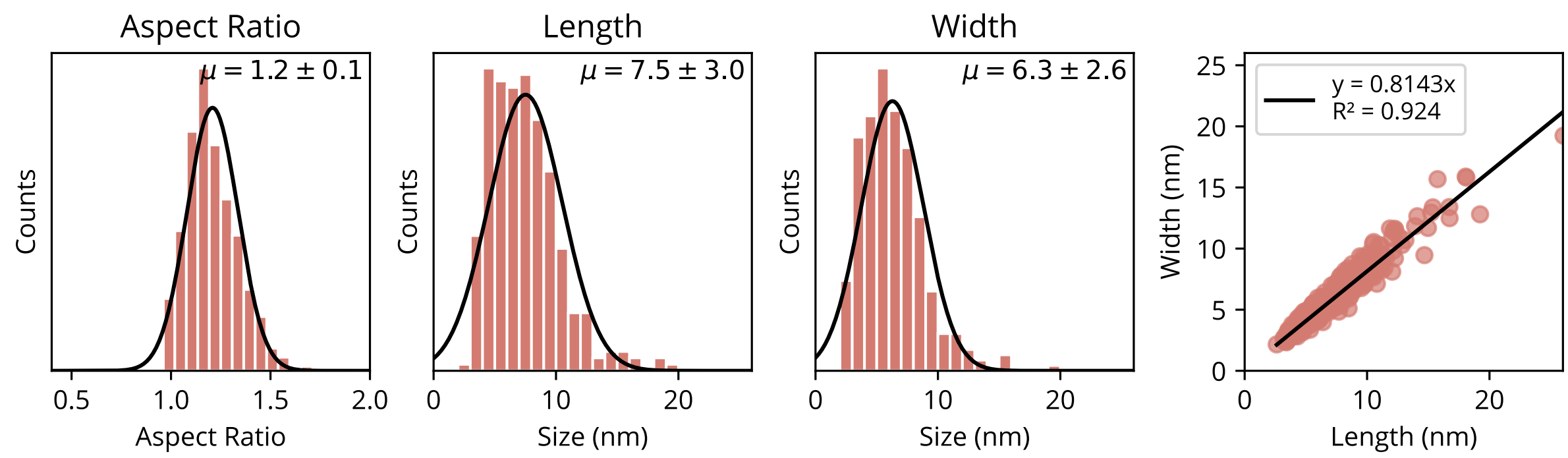

Figure S31: Aspect ratio, length, and width distributions, scattering plot of width as a function of length of Au/ZnO samples prepared with **ethanol** as hole scavenger.

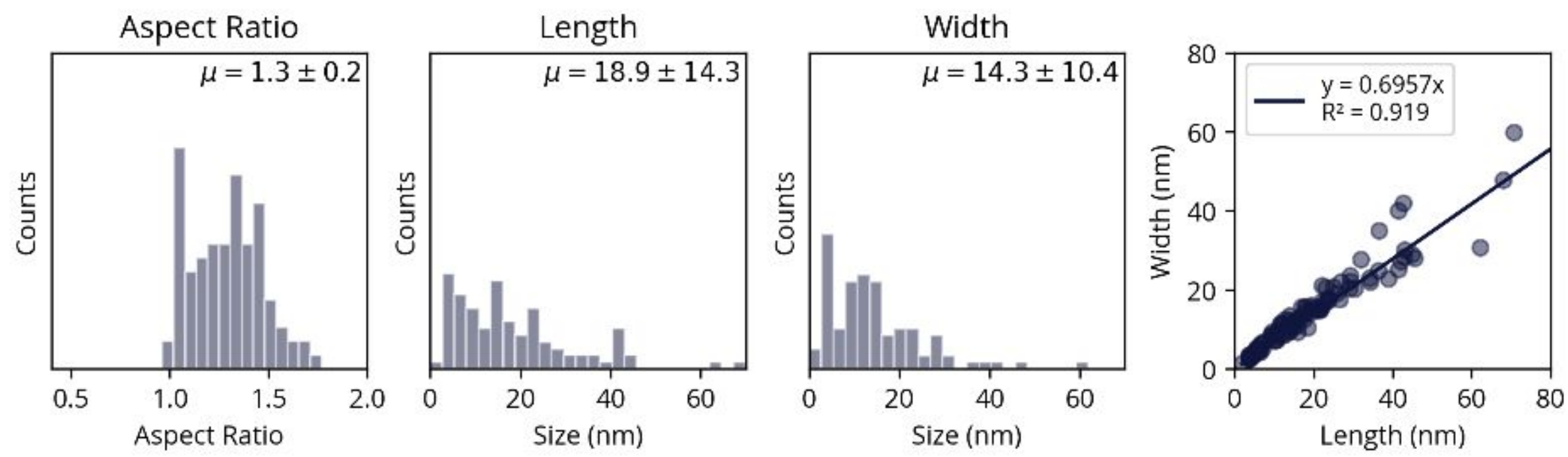

Figure S32: Aspect ratio, length, and width distributions, scattering plot of width as a function of length of Au/ZnO prepared **without** a hole scavenger with **0.6 wt.% Au/ZnO**.

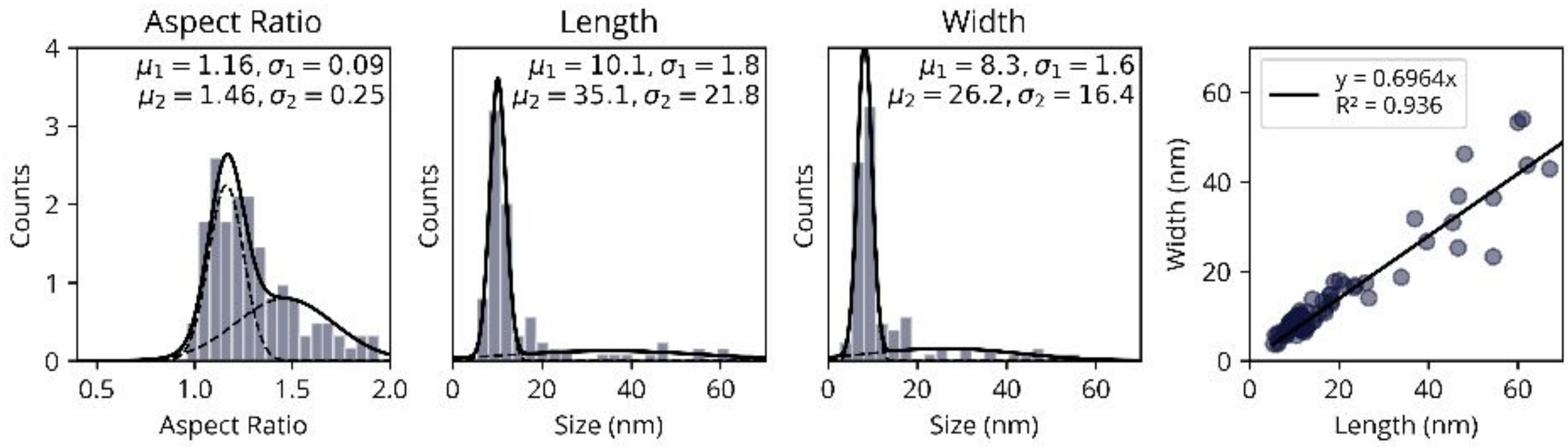

Figure S33: Aspect ratio, length, and width distributions, scattering plot of width as a function of length of Au/ZnO prepared **without** a hole scavenger with **0.9 wt.% Au/ZnO**.

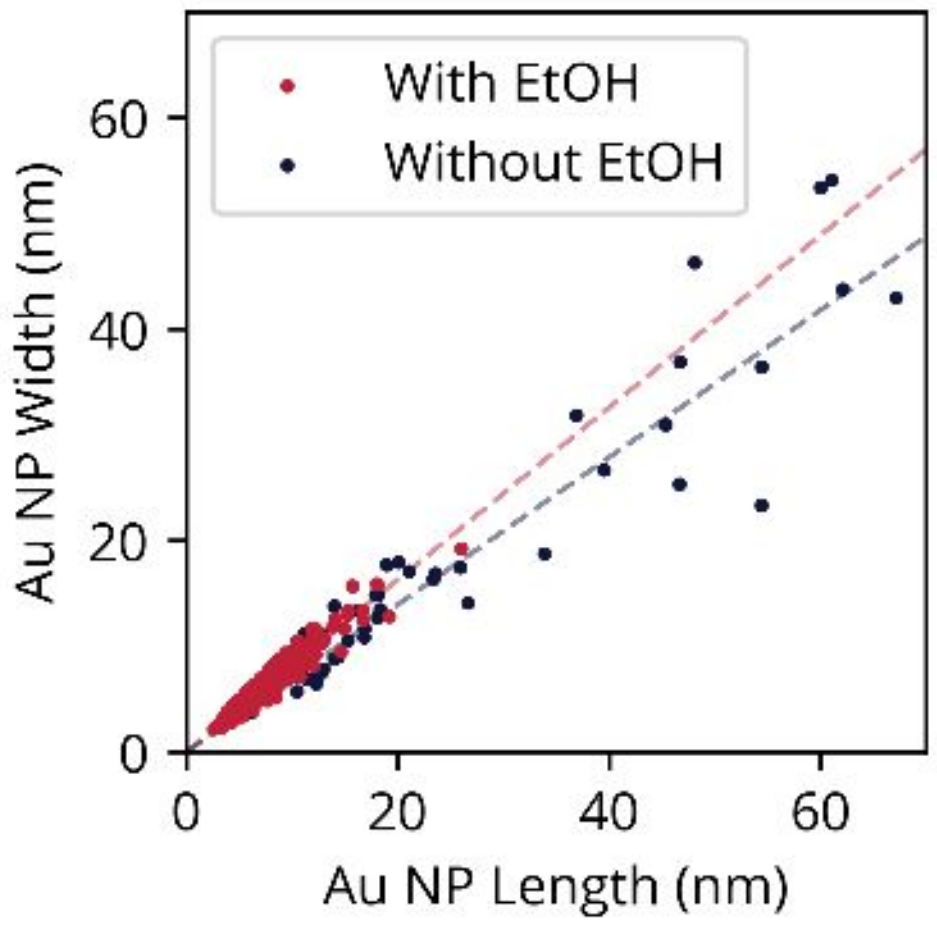

Figure S34: The relationship between Au nanoparticle (NP) width and length and their linear fit for Au/ZnO prepared with and without ethanol.

Table S4: Gold nanoparticle (Au NP) number and volume fractions for samples prepared with and without a hole scavenger (0.6 wt.% Au and 0.9 wt.% Au). By approximating the rod-shaped particle as the sum of a sphere and a cylinder. Small spheres are categorized as Au NP with a length < 30nm and AR < 1.15, small rods with length < 30 nm and AR > 1.15, large Au rods with a length > 30 nm and AR > 1.15 and large Au spheres with length > 30 nm and AR < 1.15.

	*0.9 wt.% Au-ZnO **without** ethanol*		*0.9 wt.% Au-ZnO **with** ethanol*		*0.6 wt.% Au-ZnO **without** ethanol*	
Species	Number fraction (%)	Volume fraction (%)	Number fraction (%)	Volume fraction (%)	Number fraction (%)	Volume fraction (%)
Small spheres	26.9	1.8	28.2	43.8	23.0	3.5
Small rods	59.3	4.9	61.8	56.2	59.0	16.0
Large spheres	2.8	23	0.0	0.0	4.0	16.0
Large rods	11.1	70.3	0.0	0.0	14.0	64.6

Transmission electron microscopy-weighted reconstruction of the experimental absorption spectra

To quantitatively test whether the particle size and shape distributions measured by TEM can account for the observed spectral features, we have reconstructed the ensemble absorption spectra directly from the TEM-derived particle distributions. Each measured Au nanoparticle was classified according to its aspect ratio (AR) and length. Particles with AR < 1.15 were represented as spheres, while anisotropic particles were assigned to ellipsoidal simulations with AR = 1.3, 1.6, or 2.0. Within each shape class, the simulated absorption spectrum corresponding to the particle size closest to the experimentally measured value was selected. The contribution of each particle class was then weighted by its experimentally determined population fraction, $w = \frac{N_i}{N_{total}}$, and the ensemble spectrum was calculated as

$$A_{ensemble}(\lambda) = \sum_i w_i A_i(\lambda) \quad \text{(Eq. S. 14)}$$

Because the simulated absorption magnitude is retained for each particle size rather than normalizing the individual spectra, differences in optical contribution arising from particle size are intrinsically included in the reconstruction. This analysis therefore provides a quantitative, TEM-derived estimate of the relative optical contributions of the different particle populations rather than assigning the two experimental features solely based on their expected resonance positions.

The reconstruction shows that the sphere-like and rod-like particle populations contribute at distinct spectral regions, where the sphere-like contribution is blue-shifted relative to the rod-like contribution. Furthermore, the sample prepared without EtOH contains a larger relative contribution from sphere-like particles (left figure), increasing the short-wavelength contribution to the reconstructed spectrum. Thus, the experimentally observed change in particle morphology is consistent with the increased short-wavelength absorption observed experimentally in the absence of the hole scavenger.

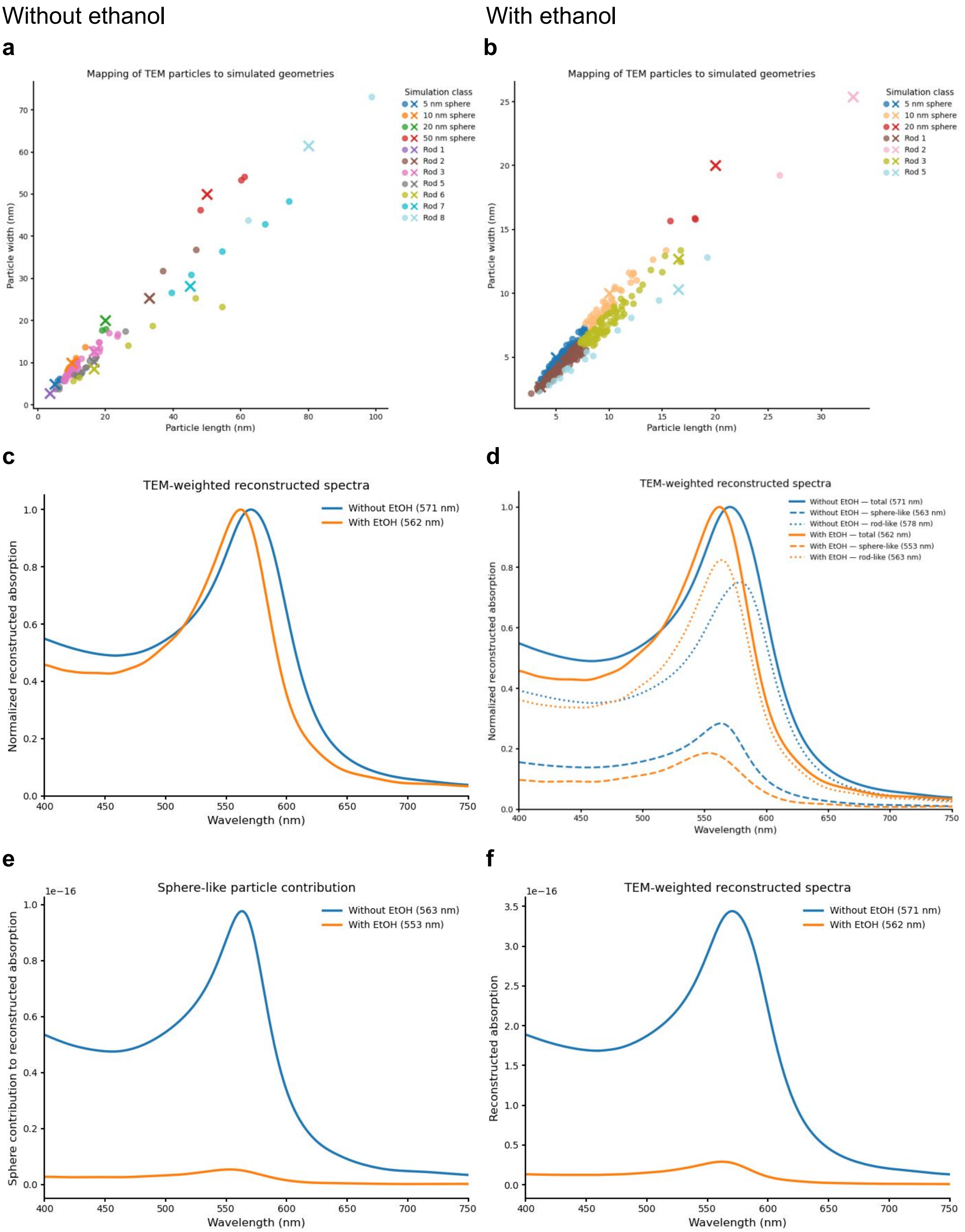

Figure S35: Transmission electron microscopy (TEM)-weighted reconstruction of the simulated Au-ZnO nanoparticle absorption spectra from the experimentally determined particle distributions. TEM-derived particle length and width are shown for samples prepared (a) without EtOH and (b) with EtOH. Each measured particle is assigned to the closest simulated geometry based on aspect ratio and particle size. Colored circles represent experimentally measured particles, while crosses indicate the corresponding simulated particle geometries. (c) Normalized total TEM-weighted reconstructed absorption spectra for samples prepared with and without EtOH. (d) Normalized total reconstructed spectra together with the corresponding sphere-like and rod-like contributions. Each sample is normalized to the maximum of its total reconstructed spectrum, retaining the relative contributions of

the sphere-like and rod-like populations. (e) Absolute sphere-like contribution to the reconstructed absorption, retaining the simulated absorption cross-sections and experimentally determined population weighting. (f) Absolute total TEM-weighted reconstructed absorption spectra. The sphere-like contribution is substantially larger for the sample prepared without EtOH, consistent with the greater abundance and optical contribution of sphere-like particles in this sample. The sphere-like contribution peaks at 563 nm without EtOH and 553 nm with EtOH, while the total reconstructed spectra peak at 571 and 562 nm, respectively.

Interparticle separation and near-field coupling

To assess the potential contribution of interparticle near-field coupling to the optical response, interparticle separations were quantified from TEM images of Au/ZnO samples prepared with and without EtOH. For 25 neighbouring particle pairs measured for each condition, the surface-to-surface interparticle gap, g, was determined directly from the TEM images. The sample prepared with EtOH exhibited smaller interparticle separations, with a median gap of 6.6 nm and a mean gap of 7.9 nm, compared with a median of 29.9 nm and a mean of 41.6 nm for the sample prepared without EtOH. In the sample prepared with EtOH, 79% of measured particle pairs had $g < 10$ nm and 100% had $g < 30$nm, compared with 11% and 50%, respectively, for the sample prepared without EtOH.

Because the strength of plasmonic coupling depends on particle separation relative to particle dimensions, the measured gaps were additionally normalized by particle size. For each particle, an equivalent diameter, D_{eq}, was calculated from the measured particle length, L, and width, W, according to

$$D_{\mathrm{eq}} = \sqrt{LW} \qquad \text{Eq. S.15}$$

For each neighbouring pair, the characteristic particle diameter, D, was taken as the mean equivalent diameter of the two particles:

$$D = \frac{D_{\mathrm{eq},1} + D_{\mathrm{eq},2}}{2} = \frac{\sqrt{L_1 W_1} + \sqrt{L_2 W_2}}{2} \qquad \text{Eq. S. 16}$$

The normalized interparticle separation was then calculated as

$$\frac{g}{D} = \frac{2g}{\sqrt{L_1 W_1} + \sqrt{L_2 W_2}} \qquad \text{Eq. S. 17}$$

Near-field coupling can induce shifts in the LSPR peak wavelength, with the magnitude of the coupling-induced shift decreasing approximately exponentially with increasing normalized interparticle separation, $g/$. Using this normalization, the median g/D was 1.47 for the sample prepared with EtOH and 2.81 for the sample prepared without EtOH. Furthermore, 32% of the particle pairs in the EtOH sample exhibited $g/D < 1$, compared with 14% of the pairs in the sample without EtOH. These measurements indicate that the particles prepared in the presence of EtOH are, on average, more closely spaced both in terms of absolute separation and separation relative to particle size. Previous plasmon-ruler studies show that the largest spectral shifts occur for closely spaced particles, particularly for $g/D < 1$, while the shift rapidly approaches zero as the separation increases relative to the particle diameter.[13] In the present samples, the median g/D values (1.47 with EtOH and 2.81 without EtOH) indicate that the majority of particle pairs lie outside the range in which strong coupling-induced spectral shifts are typically observed. Nevertheless, 32% of particle pairs with EtOH and 14% without EtOH exhibit $g/D < 1$, and near-field coupling may therefore contribute to the spectra for a subset of closely spaced particles.

Furthermore, the magnitude and direction of coupling-induced changes in the LSPR depend not only on interparticle separation but also on the relative particle arrangement and excitation

polarization.[14] Although near-field coupling is expected to be more relevant for the EtOH sample due to the smaller absolute and normalized interparticle gaps, the broad distributions of particle separations, sizes, and relative orientations are expected to result in a heterogeneous distribution of coupling interactions rather than a uniform spectral shift.

Table S5: Overview of the particle sizes and localized surface plasmon resonance (LSPR) of photo-deposited Au/ZnO in the open literature, including the conditions, light source, irradiation time, used solvent and hole scavenger as well as the research domain and application of the materials made. NA = Not available. PD = Photodeposition.

Refe-rence	Research/ application	Conditions	Light source + irradiation time	Solvent + Hole scavenger	Particle size (nm)	LSPR (nm)
[15]	Water content during PD	N_2 purged PD of 145 μM $HAuCl_4.3H_2O$ in 1.9 mM ZnO in 2-propanol	303 ± 10 nm + not defined	Water (0.15% V/V) in 2-propanol	15	530
[16]	Photocatalytic azo dye decolorization	0.156 mM $HAuCl_4$ and 2 mg/mL ZnO/thiourea in water, 60 min stirring	Black light fluorescent tube (25 W), UV-A + 10 min	Water	22.4	Broadband with max. at 570
[17,18]	Photocatalytic MB degradation	0.1 mM $HAuCl_4.3H_2O$ on ZnO NRs on glass substrate	UV (18W) + 10 min	Water	7 to 23	525
[19]	CO oxidation	$HAuCl_4.3H_2O$ and ZnO sonicated 30 min before irradiation	Heraeus TNN 15/32 253.7 nm (3 W) + 2 h	water and methanol (300 mL, 15:1 ratio)	12.2 ± 0.7	NA
[20]	Photocatalytic Nox degradation	Semiconductor slurry sonicated for 1 h, HS added, $HAuCl_4.3H_2O$, pH adjusted to 7, Ar-purged for 30 min	125 W high-pressure Hg lamp (Ushio) operated at 25 mW cm^2 and 313 K + 30 min	1:1 (v/v) water/methanol	30 ± 13	700
[21]	Charge redistribution in Au/ZnO	ZnO (0.20 mg/mL), gold(III)-chloride (0.01 mg/mL), in chloroform	UV 254 nm, 2−10 min	Chloroform	3−4	530
[22]	Modified PD of Au on ZnO	ZnO/Si substrate immersed in 0.25 mM $HAuCl_4$, citrate, MeOH	UV lamp (100 W) + 30 min	Water, citrate (citrate/Au^{3+} = 20, 0.5 ml MeOH	7.5 ± 3	NA
[23]	Facet-specific PD for TB degradation	6 min pre-irradiation of $HAuCl_4.3H_2O$ in EtOH (0.1375 mM), ZnO NPs (0.4 mg/mL) 10 min	UV diode (35 mW/cm^2) 370 ± 10 nm + not defined	EtOH<br>EtOH/toluene	Plates<br>7 ± 2<br>17 ± 7	NA
					Cones<br>6 ± 2<br>11 ± 3	

		sonicated, Ar-purged for 30 min, 5 s on – 30 s off pulsed light			Bullet<br>6 ± 2<br>6 ± 2<br>Rods<br>6 ± 2<br>8 ± 3	
[24]	H_2 sensors	$HAuCl_4.3H_2O$ and ZnO in water:MeOH	Not defined + 2h	Water: MeOH = 7:3	-	NA
[25]	CO oxidation in H_2-rich gas	0.005 M $HAuCl_4.3H_2O$ and ZnO in water, pH adjusted to 8 with Na_2CO_3	Philips TUV PL-S 11W 2pin G23 Entkeimungslampe, 253.7 nm + 240 min	Water, Na_2CO_3	2.25	Broadband centered at 550
11	Controlling gold PD	6 min pre-irradiation of $HAuCl_4.3H_2O$ in EtOH (0.1375 mM), ZnO NPs (0.4 mg/mL) 10 min sonicated, Ar-purged for 30 min, 5 s on – 30 s off pulsed light	UV diode (35 mW/cm^2) 370 ± 10 nm + 20 s	EtOH<br>EtOH/toluene<br>EtOH, acetonitrile<br>EtOH/butanol<br>EtOH/t-butanol<br>EtOH/cyclohexane<br>EtOH/MeOH	14 ± 2<br>6 ± 2<br>22 ± 2<br>10 ± 2<br>12 ± 2<br>12 ± 4<br>18 ± 2	NA
[26]	ROS in ZnO and Au/ZnO	ZnO suspension and $HAuCl_4$ sonicated for 3 min	450 W Xe lamp + 15 min	water	0.77 ± 0.2<br>1.4 ± 0.2<br>2.3 ± 0.4<br>2.4 ± 0.3	Grey color, so broad band absorption
[27]	Preferential CO oxidation	$HAuCl_4.3H_2O$ and ZnO in water, pH adjusted to 8 with Na_2CO_3	UV–vis lamp (11 W) + 1 h, 3 h, and 5 h	water	4.4 ± 0.68<br>3.2 ± 0.58<br>4.0 ± 0.80	550
[28]	PD Au on ZnO	ZnO/Si substrate immersed in $HAuCl_4$	16 W UV lamp (254 nm) + 3h	Water	30 nm	550
[29]	PD Au on ZnO	ZnO/Si substrate immersed in $HAuCl_4$	365 nm with 150 mW/cm^2 + 10 min	Ethanol	30 nm<br>15 nm<br>5 nm	NA
5	Photoelectrochemical Water Splitting	ZnO NRs on Zn foil immersed in $HAuCl_4$	300 W xenon lamp + 4 h	Water	6.5 ± 0.5	Broadband with a peak at 480
[30]	PD Au on ZnO nano ires	50 mg ZnO in 9 ml $HAuCl_4$ (540 mM) sonicated for 10 s, 1 ml MeOH added, Ar-purged for 30 min while stirring	2 UV diodes λ = 365 nm, I = 120 mW cm^2, HLV-24UV365-4WNRBTNJ + 20 s and 10 min	Water + methanol (2.47 M)	5.9 ± 1.6<br>12.0 ± 3.0	NA

31	PD Au on ZnO nanopyramids	0.5 ml ZnO (41 g/L) in EtOH, 12 ml toluene, 0.1-0.5 ml 10 mM $HAuCl_4.3H_2O$	Philips HPR, 125WUV mercury vapor high-pressure lamp, 360 nm, 1-10 min	Ethanol, toluene	8, 10, 20	550
32	Degradation of benzene	1 ml sodium citrate, 1 ml ascorbic acid, 1 ml $HAuCl_4$ (all 1 mM) in water droplet on ZnO nanowires on ZnO foil	UV lamp 8W, 30 min	Water, sodium citrate, ascorbic acid	NA	550
33	Photoelectrochemical water splitting	ZnO NR@NP immersed in 1 mL $HAuCl_4$ (0.3 mM) in 100 mL of DI water	150 W Xe lamp, 2-6 h	Water	5–7	540

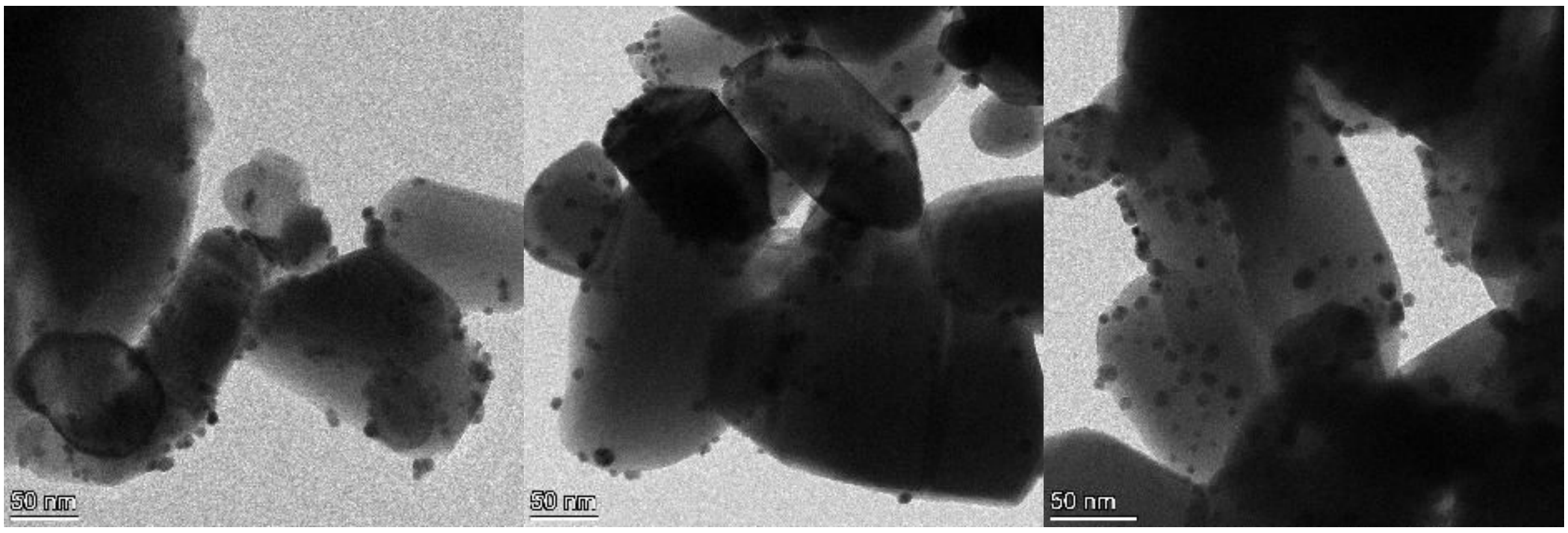

Figure S36: Transmission electron microscopy (TEM) images of Au/ZnO prepared with **methanol** as hole scavenger.

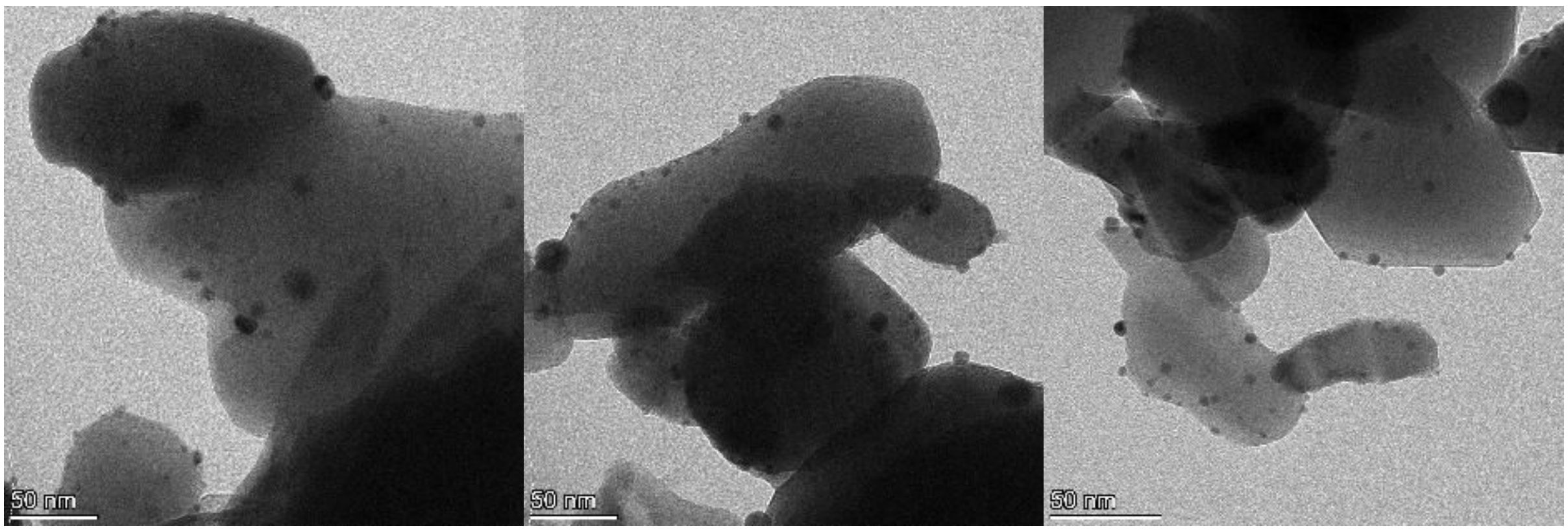

Figure S37: Transmission electron microscopy (TEM) images of Au/ZnO prepared with **ethanol** as hole scavenger.

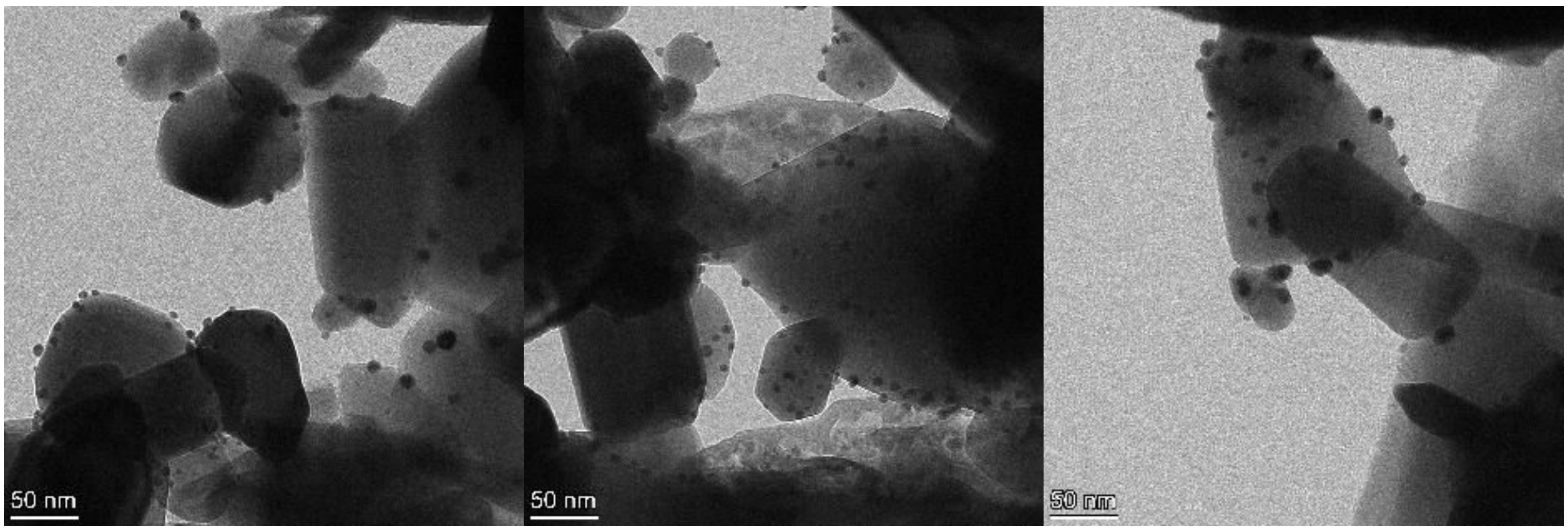

Figure S38: Transmission electron microscopy (TEM) images of Au/ZnO prepared with ***iso*-propanol** as hole scavenger.

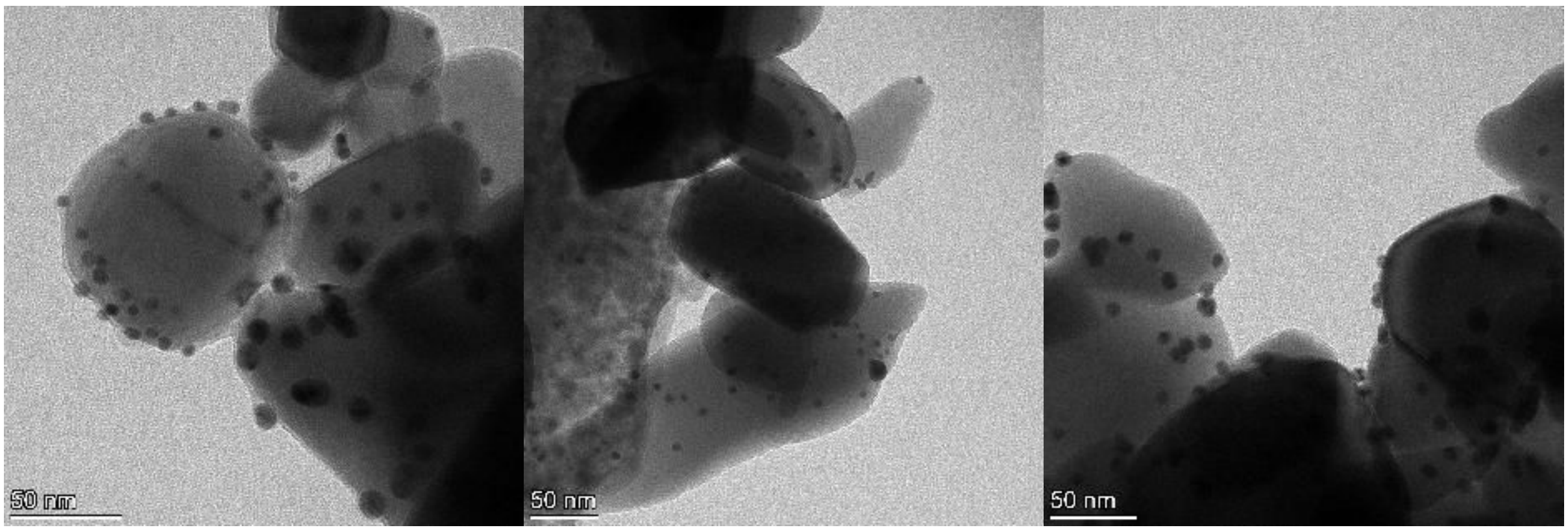

Figure S39: Transmission Electron Microscopy (TEM) images of Au/ZnO prepared with ***tert*-butanol** as hole scavenger.

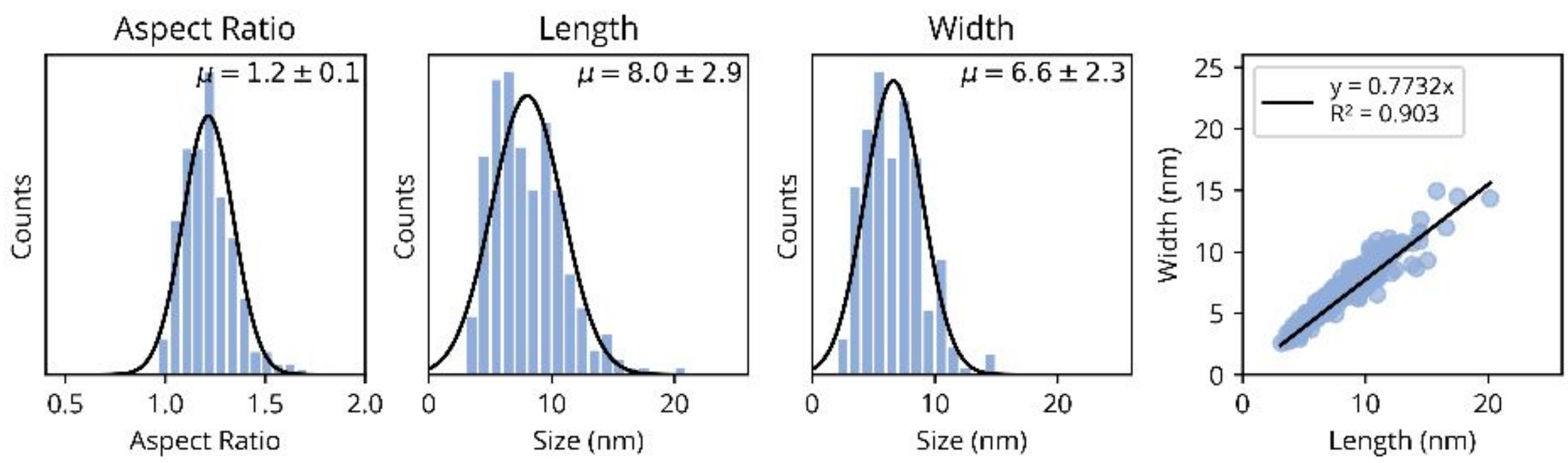

Figure S40: Aspect ratio, length, and width distributions, scattering plot of width as a function of length of Au/ZnO samples prepared with **methanol** as hole scavenger.

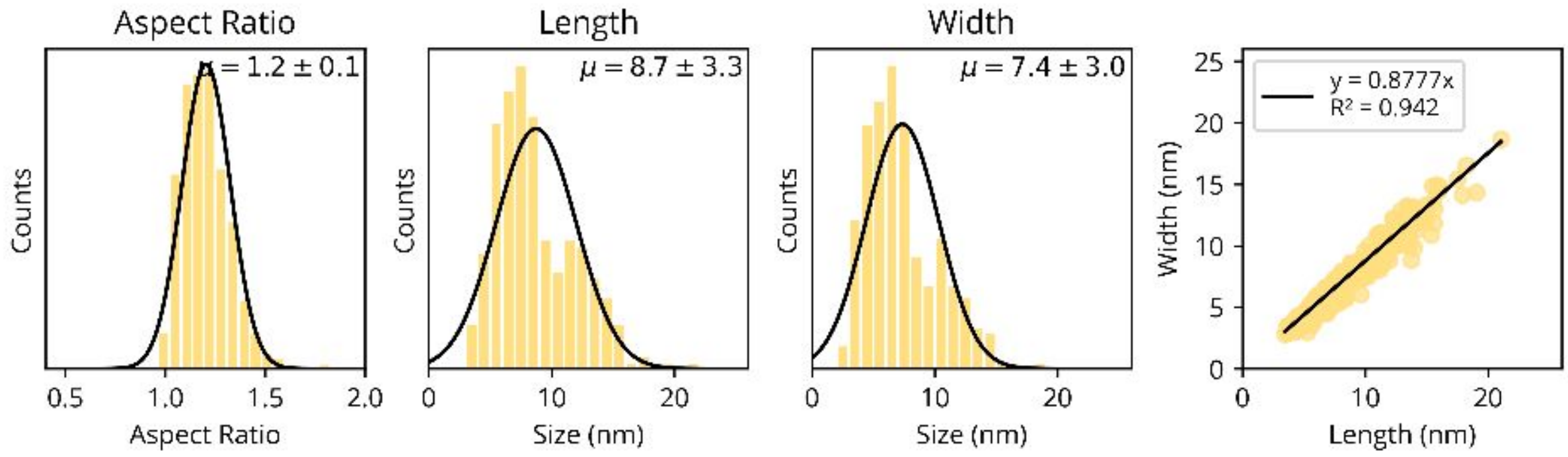

Figure S41: Aspect ratio, length, and width distributions, scattering plot of width as a function of length of Au/ZnO samples prepared with ***iso*-propanol** as hole scavenger.

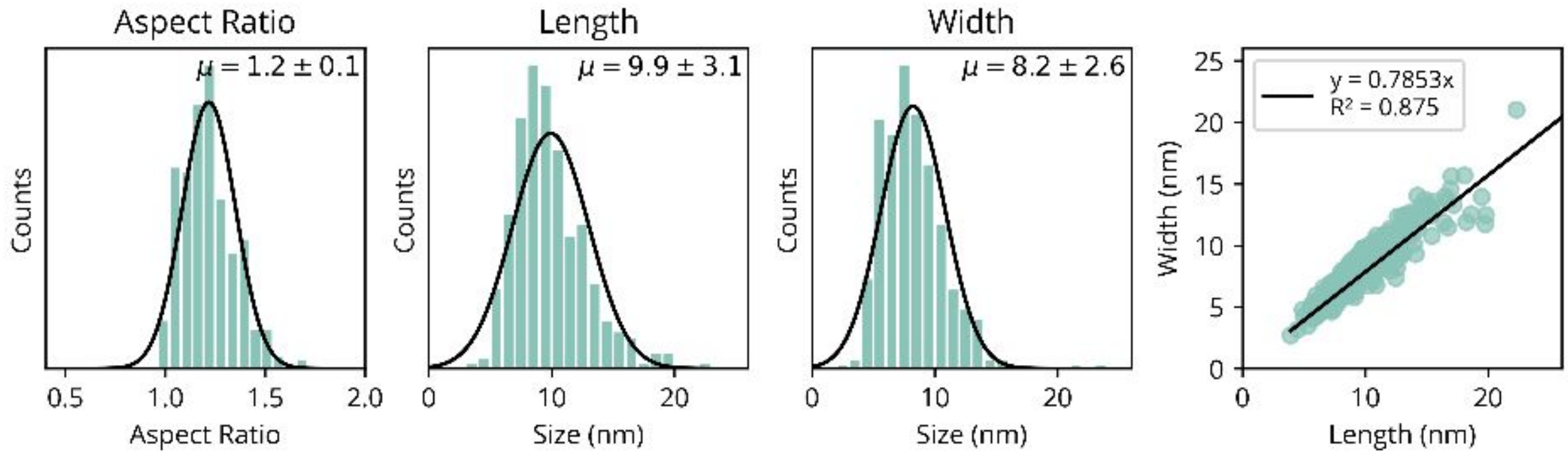

Figure S42: Aspect ratio, length, and width distributions, scattering plot of width as a function of length of Au/ZnO samples prepared with ***tert*-butanol** as hole scavenger.

Table S6: Batch-to-batch reproducibility of Au nanoparticle dimensions for each preparation condition (with different hole scavengers added, without a hole scavenger present, and with oxygen conditions), determined by transmission electron microscopy (TEM). For each hole scavenger, the reaction was performed in 2–3 independent batches. Values are reported as mean ± standard deviation per batch. N denotes the number of particles analyzed. The combined statistics for each hole scavenger (bold rows), correspond to the combined dataset shown in Figure 4e-g and histograms shown in Figures S29-30.

**Preparation condition**	**Batch**	**N (# particles)**	**Length (nm)**	**Width (nm)**	**Aspect ratio**
Methanol	1	119	8.1 ± 2.6	6.9 ± 2.2	1.2 ± 0.1
Methanol	2	119	7.9 ± 3.1	6.4 ± 2.5	1.2 ± 0.1
**Methanol**	**Combined**	**238**	**8.0 ± 2.9**	**6.6 ± 2.3**	**1.2 ± 0.1**
Ethanol	1	106	7.8 ± 2.8	6.6 ± 2.5	1.2 ± 0.1
Ethanol	2	113	5.8 ± 2.1	4.8 ± 1.8	1.2 ± 0.1
Ethanol	3	106	9.0 ± 3.1	7.5 ±2.6	1.2 ± 0.1
**Ethanol**	**Combined**	**325**	**7.5 ± 3.0**	**6.3 ± 2.6**	**1.2 ± 0.1**
Iso-propanol	1	102	9.9 ± 3.3	8.6 ± 2.9	1.2 ± 0.1
Iso-propanol	2	100	8.4 ± 3.4	7.0 ±2.9	1.2 ± 0.1
Iso-propanol	3	102	7.9 ± 2.8	6.5 ± 2.6	1.2 ± 0.1
***Iso*-propanol**	**Combined**	**304**	**8.7 ± 3.3**	**7.4 ± 3.0**	**1.2 ± 0.1**
Tert-butanol	1	121	11.1 ± 3.5	9.4 ± 2.9	1.2 ± 0.1
Tert-butanol	2	101	9.0 ± 2.7	7.4 ± 2.3	1.2 ± 0.1
Tert-butanol	3	122	9.5 ± 2.6	7.8 ± 2.2	1.2 ± 0.1
***Tert*-butanol**	**Combined**	**344**	**9.9 ± 3.1**	**8.2 ± 2.6**	**1.2 ± 0.1**
Without (0.6 wt.% Au)	1	90	19.5 ± 14.8	14.8 ± 10.8	1.3 ± 0.2
Without (0.6 wt.% Au)	2	10	13.5 ± 8.4	10.4 ± 5.3	1.3 ± 0.2
Without (0.6 wt.% Au)	**Combined**	**100**	**18.9 ± 14.3**	**14.3 ± 10.4**	**1.3 ± 0.2**
**Without (0.9 wt.% Au)**	**1**	**108**	**17.7 ± 16.8**	**13.4 ± 12.1**	**1.3 ± 0.2**
**With oxygen**	**1**	**96**	**18.9 ± 7.1**	**14.9 ± 4.8**	**1.3 ± 0.2**

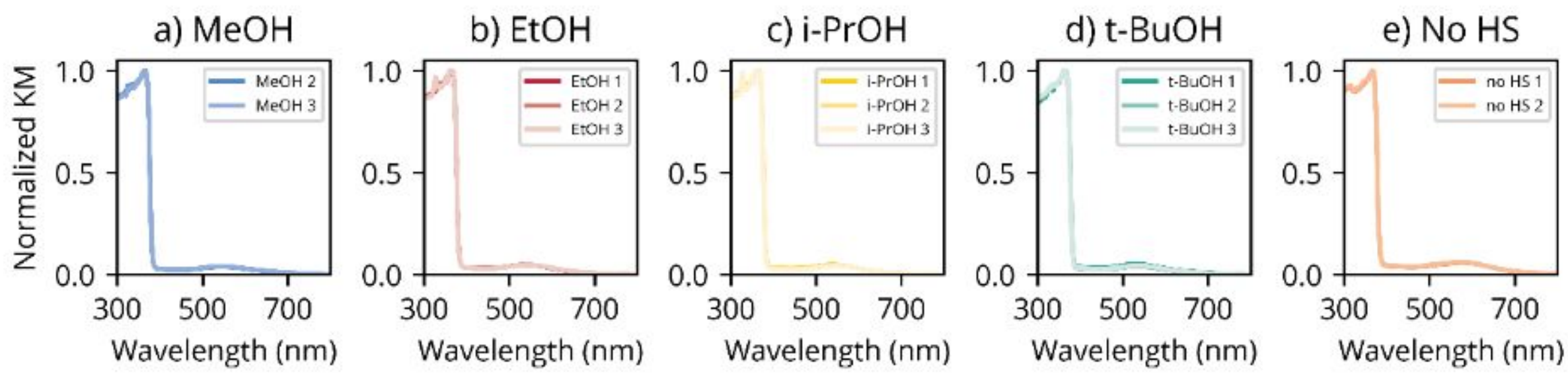

Figure S43: Kubelka-Munk (K-M) function applied to Diffuse Reflectance (DR) UV-Vis spectroscopy results normalized to the ZnO absorption band of dried Au/ZnO samples photo-deposited with a) methanol, b) ethanol, c) *iso*-propanol, d) *tert*-butanol, and e) without a hole scavenger.

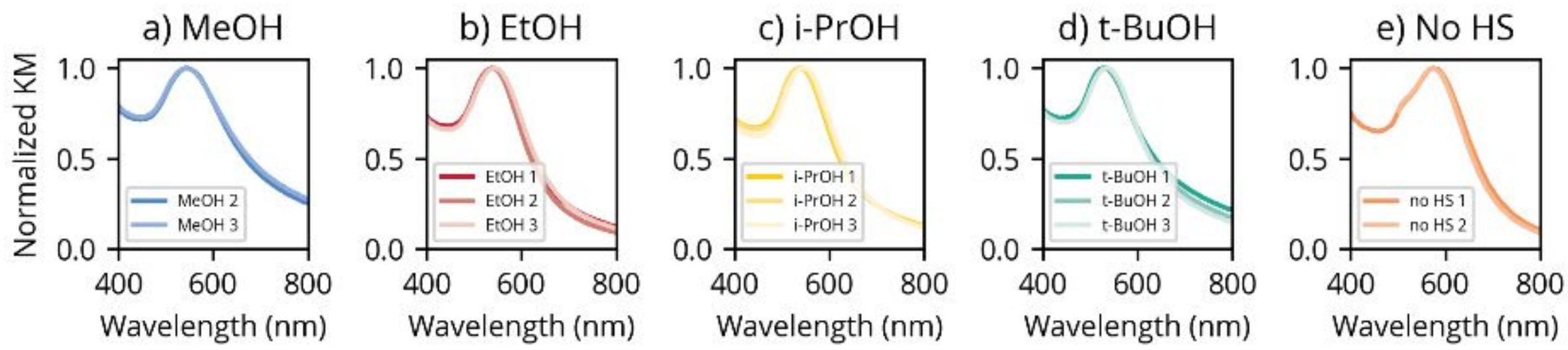

Figure S44: Kubelka-Munk (KM) function applied to Diffuse Reflectance (DR) UV-Vis spectroscopy results normalized to the Au absorption band of dried Au/ZnO samples photo-deposited with a) methanol, b) ethanol, c) iso-propanol, d) tert-butanol, and e) without a hole scavenger.

References

(1) Kavitha, R.; Kumar, S. G. A Review on Plasmonic Au-ZnO Heterojunction Photocatalysts: Preparation, Modifications and Related Charge Carrier Dynamics. *Materials Science in Semiconductor Processing* **2019**, *93*, 59-91.
(2) Hattori, Y.; Gutiérrez Álvarez, S.; Meng, J.; Zheng, K.; Sá, J. Role of the Metal Oxide Electron Acceptor on Gold–Plasmon Hot-Carrier Dynamics and Its Implication to Photocatalysis and Photovoltaics. *ACS Applied Nano Materials* **2021**, *4*, 2052-2060.
(3) He, W.; Jia, H.; Cai, J.; Han, X.; Zheng, Z.; Wamer, W. G.; Yin, J.-J. Production of Reactive Oxygen Species and Electrons from Photoexcited ZnO and ZnS Nanoparticles: A Comparative Study for Unraveling their Distinct Photocatalytic Activities. *The Journal of Physical Chemistry C* **2016**, *120*, 3187-3195.
(4) Al-Azri, Z. H. N.; Chen, W.-T.; Chan, A.; Jovic, V.; Ina, T.; Idriss, H.; Waterhouse, G. I. N. The roles of metal co-catalysts and reaction media in photocatalytic hydrogen production: Performance evaluation of M/TiO_2 photocatalysts (M = Pd, Pt, Au) in different alcohol–water mixtures. *Journal of Catalysis* **2015**, *329*, 355-367.
(5) Wu, M.; Chen, W. J.; Shen, Y. H.; Huang, F. Z.; Li, C. H.; Li, S. K. *In-Situ* Growth of Matchlike ZnO/Au Plasmonic Heterostructure for Enhanced Photoelectrochemical Water Splitting. *ACS Applied Materials and Interfaces* **2014**, *6*, 15052-15060.
(6) Gómez-Marín, A. M.; Feliu, J. M. Oxygen Reduction on Platinum Single Crystal Electrodes. In *Encyclopedia of Interfacial Chemistry*, Wandelt, K. Ed.; Elsevier, 2018; pp. 820-830.
(7) Yang, L.; Zhou, H.; Fan, T.; Zhang, D. Semiconductor photocatalysts for water oxidation: current status and challenges. *Physical Chemistry Chemical Physics* **2014**, *16*, 6810-6826.
(8) Burek, B. O.; Timm, J.; Bahnemann, D. W.; Bloh, J. Z. Kinetic Effects and Oxidation Pathways of Sacrificial Electron Donors on the Example of the Photocatalytic Reduction of Molecular Oxygen to Hydrogen Peroxide over Illuminated Titanium Dioxide. *Catalysis Today* **2019**, *335*, 354-364.
(9) Dimitropoulos, M.; Aggelopoulos, C. A.; Sygellou, L.; Tsantis, S. T.; Koutsoukos, P. G.; Yannopoulos, S. N. Unveiling the photocorrosion mechanism of zinc oxide photocatalyst: Interplay between surface corrosion and regeneration. *Journal of Environmental Chemical Engineering* **2024**, *12*, 112102-112112.
(10) Domenech, J.; Prieto, A. Stability of zinc oxide particles in aqueous suspensions under UV illumination. *The Journal of Physical Chemistry* **1986**, *90*, 1123-1126.
(11) Fernando, J. F. S.; Shortell, M. P.; Noble, C. J.; Harmer, J. R.; Jaatinen, E. A.; Waclawik, E. R. Controlling Au Photodeposition on Large ZnO Nanoparticles. *ACS Applied Materials & Interfaces* **2016**, *8*, 14271-14283.
(12) McGilvray, K. L.; Granger, J.; Correia, M.; Banks, J. T.; Scaiano, J. C. Opportunistic Use of Tetrachloroaurate Photolysis in the Generation of Reductive Species for the Production of Gold Nanostructures. *Physical Chemistry Chemical Physics* **2011**, *13*, 11914–11918.

(13) Jain, P. K.; Huang, W.; El-Sayed, M. A. On the Universal Scaling Behavior of the Distance Decay of Plasmon Coupling in Metal Nanoparticle Pairs: A Plasmon Ruler Equation. *Nano Letters* **2007**, *7*, 2080-2088.
(14) Rechberger, W.; Hohenau, A.; Leitner, A.; Krenn, J. R.; Lamprecht, B.; Aussenegg, F. R. Optical properties of two interacting gold nanoparticles. *Optics Communications* **2003**, *220*, 137-141.
(15) Aguirre, M. E.; Custo, G.; Goes, M. S.; Bueno, P. R.; Zampieri, G.; Grela, M. A. Critical Water Effect on the Plasmon Band and Visible Light Activity of Au/ZnO Nanocomposites. *The Journal of Physical Chemistry C* **2014**, *118*, 2018-2027.
(16) Andrade, G. R. S.; Nascimento, C. C.; Silva Júnior, E. C.; Mendes, D. T. S. L.; Gimenez, I. F. ZnO/Au Nanocatalysts for Enhanced Decolorization of an Azo Dye Under Solar, UV-A and Dark Conditions. *Journal of Alloys and Compounds* **2017**, *710*, 557-566.
(17) Bora, T.; Myint, M. T. Z.; Al-Harthi, S. H.; Dutta, J. Role of surface defects on visible light enabled plasmonic photocatalysis in Au–ZnO nanocatalysts. *RSC Advances* **2015**, *5*, 96670-96680.
(18) Bora, T.; Zoepfl, D.; Dutta, J. Importance of Plasmonic Heating on Visible Light Driven Photocatalysis of Gold Nanoparticle Decorated Zinc Oxide Nanorods. *Scientific Reports* **2016**, *6*, 26913-26923.
(19) Carabineiro, S. A. C.; MacHado, B. F.; Bacsa, R. R.; Serp, P.; Draić, G.; Faria, J. L.; Figueiredo, J. L. Catalytic Performance of Au/ZnO Nanocatalysts for CO Oxidation. *Journal of Catalysis* **2010**, *273*, 191-198.
(20) Castelló Lux, K.; Hot, J.; Fau, P.; Bertron, A.; Kahn, M. L.; Ringot, E.; Fajerwerg, K. Nano-gold Decorated ZnO: An Alternative Photocatalyst Promising for NO_x Degradation. *Chemical Engineering Science* **2023**, *267*, 118377-118390.
(21) Chanaewa, A.; Schmitt, J.; Meyns, M.; Volkmann, M.; Klinke, C.; Von Hauff, E. Charge Redistribution and Extraction in Photocatalytically Synthesized Au–ZnO Nanohybrids. *The Journal of Physical Chemistry C* **2015**, *119*, 21704-21710.
(22) Cure, J.; Assi, H.; Cocq, K.; Marìn, L.; Fajerwerg, K.; Fau, P.; Bêche, E.; Chabal, Y. J.; Estève, A.; Rossi, C. Controlled Growth and Grafting of High-Density Au Nanoparticles on Zinc Oxide Thin Films by Photo-Deposition. *Langmuir* **2018**, *34*, 1932-1940.
(23) De Corrado, J. M.; Fernando, J. F. S.; Shortell, M. P.; Poad, B. L. J.; Blanksby, S. J.; Waclawik, E. R. ZnO Colloid Crystal Facet-Type Determines both Au Photodeposition and Photocatalytic Activity. *ACS Applied Nano Materials* **2019**, *2*, 7856-7869.
(24) Dong, H.-D.; Zhao, J.-P.; Peng, M.-X.; Zhang, Y.-H.; Xu, P.-Y. Au-modified Spindle ZnO for High Efficiency H_2 Sensors. *Vacuum* **2023**, *207*, 111597-111605.
(25) Dulnee, S.; Luengnaruemitchai, A.; Wanchanthuek, R. Activity of Au/ZnO catalysts prepared by photo-deposition for the preferential CO oxidation in a H_2-rich gas. *International Journal of Hydrogen Energy* **2014**, *39*, 6443-6453.
(26) He, W.; Kim, H. K.; Wamer, W. G.; Melka, D.; Callahan, J. H.; Yin, J. J. Photogenerated Charge Carriers and Reactive Oxygen Species in ZnO/Au Hybrid Nanostructures with Enhanced Photocatalytic and Antibacterial Activity. *Journal of the American Chemical Society* **2014**, *136*, 750-757.
(27) Naknam, P.; Luengnaruemitchai, A.; Wongkasemjit, S. Preferential CO oxidation over Au/ZnO and Au/ZnO–Fe_2O_3 catalysts prepared by photodeposition. *International Journal of Hydrogen Energy* **2009**, *34*, 9838-9846.
(28) Su, L.; Qin, N. A facile method for fabricating Au-nanoparticles-decorated ZnO nanorods with greatly enhanced near-band-edge emission. *Ceramics International* **2015**, *41*, 2673-2679.
(29) Wu, J.-J.; Tseng, C.-H. Photocatalytic properties of nc-Au/ZnO nanorod composites. *Applied Catalysis B: Environmental* **2006**, *66*, 51-57.
(30) Yan, Y.; Kobayashi, H.; Tada, H.; Soejima, T. Site-Selective and High-Density Gold Nanoparticle Photodeposition on the Edges of ZnO Nanowires. *Journal of Physical Chemistry Letters* **2025**, *16*, 1409-1414.
(31) Yao, K. X.; Liu, X.; Zhao, L.; Zeng, H. C.; Han, Y. Site-specific growth of Au particles on ZnO nanopyramids under ultraviolet illumination. *Nanoscale* **2011**, *3*, 4195-4200.

(32) Yu, H.; Ming, H.; Zhang, H.; Li, H.; Pan, K.; Liu, Y.; Wang, F.; Gong, J.; Kang, Z. Au/ZnO Nanocomposites: Facile Fabrication and Enhanced Photocatalytic Activity for Degradation of Benzene. *Materials Chemistry and Physics* **2012**, *137*, 113-117.
(33) Zhang, C.; Shao, M.; Ning, F.; Xu, S.; Li, Z.; Wei, M.; Evans, D. G.; Duan, X. Au nanoparticles sensitized ZnO nanorod@nanoplatelet core–shell arrays for enhanced photoelectrochemical water splitting. *Nano Energy* **2015**, *12*, 231-239.
